# Enantioselective Synthesis of Chromanones Bearing an α,α-Disubstituted α-Amino Acid Moiety via Decarboxylative Michael Reaction

**DOI:** 10.3390/molecules24142565

**Published:** 2019-07-15

**Authors:** Jan Bojanowski, Lesław Sieroń, Anna Albrecht

**Affiliations:** 1 Institute of Organic Chemistry, Faculty of Chemistry, Lodz University of Technology, Żeromskiego 116, 90-924 Łódź, Poland; 2 Institute of General and Ecological Chemistry, Faculty of Chemistry, Lodz University of Technology, Żeromskiego 116, 90-924 Łódź, Poland

**Keywords:** organocatalysis, Michael addition, azlactones, α,α-disubstituted α-amino acids, decarboxylation

## Abstract

In this manuscript, a novel, decarboxylative Michael reaction between α-substituted azlactones and chromone-3-carboxylic acids is described. The reaction proceeds in a sequence Michael addition followed by decarboxylative deprotonation, and it results in the formation of chromanones bearing an azlactone structural unit. The possibility of transforming an azlactone moiety into a protected α,α-disubstituted α-amino acid derivative is also demonstrated.

## 1. Introduction

**Scheme 1:**
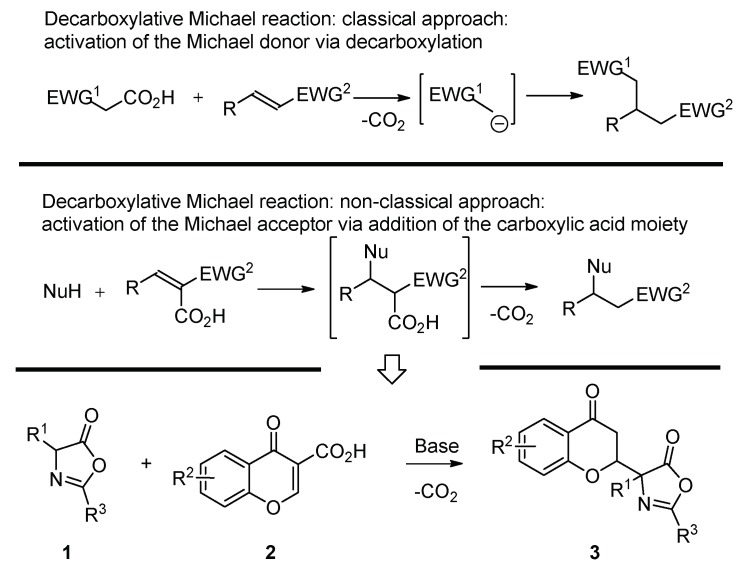
Decarboxylative Michael reactions.

The Michael reaction constitutes one of the most fundamental C–C and C–X bond-forming reactions, allowing access to various useful building blocks for organic synthesis [1,2,3,4,5,6,7,8,9,10]. Its decarboxylative [11] variant has also been described in literature [12,13,14,15,16,17,18,19,20,21,22,23,24,25,26,27,28,29,30,31,32,33,34,35,36,37,38]. The most common strategy utilized to realize the decarboxylative Michael reaction relies on the activation of Michael donors via decarboxylation (Scheme 1, top) [12,13,14,15,16,17,18,19,20,21,22,23,24,25,26,27,28,29,30] with malonic acid half-thioesters (MAHT) and related systems [16,17,18,19,20,21,22,23]. The decarboxylation of these molecules leads to the generation of stabilized carbanions readily participating in the subsequent Michael addition. An alternative strategy relies on the activation of the Michael acceptor via the addition of the carboxylic acid group in its α-position (Scheme 1, middle) [31,32,33,34,35,36,37,38]. In such a manner, the electrophilic property of the Michael acceptor is enhanced with the carboxylic acid moiety being readily removed via the decarboxylation of the originally formed Michael adduct. Surprisingly, such decarboxylative Michael reactions [31,32,33,34,35,36,37,38] are very unique in literature, with their enantioselective variant reported, to the best of our knowledge, only in the case of doubly decarboxylative reactions involving MAHT as a Michael donor [23,38].

## 2. Results

**Scheme 2:**
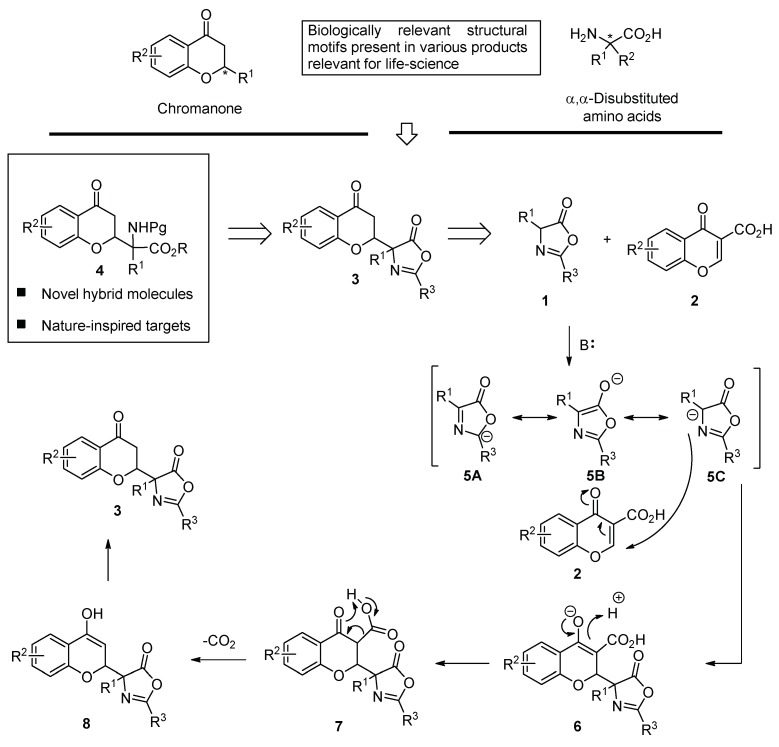
The relevance of a chromanone, α,α-disubstituted amino acid structural motifs, and new hybrid molecules being the objectives of this work.

Chromanones and their related heterocyclic ring system (Scheme 2, top to the left) are widely distributed in natural products and biologically active molecules [39,40,41,42,43,44,45,46,47,48,49,50,51,52,53,54,55,56,57,58]. Similarly, compounds with an incorporated α,α-disubstituted amino acid moiety exhibit diverse biological activity [59,60,61,62,63,64]. Therefore, given the importance of chromanones and α,α-disubstituted amino acids, the incorporation of both structural motifs into one hybrid molecule seemed like a very attractive synthetic task (Scheme 2, top). It was envisioned that **3** bearing an azlactone moiety will serve as a direct precursor of **4** due to the well-established ability of the oxazol-5-(4*H*)-one ring to be transformed into the corresponding α,α-disubstituted amino acid moiety [65,66,67,68,69,70,71,72]. It was anticipated that the products **3** should be accessible from α-substituted azlactone **1** and chromone-3-carboxylic acid **2** via a decarboxylative Michael reaction. The mechanism of this transformation is shown in the bottom of Scheme 2. It is initiated through the deprotonation of **1** to give an aromatic anion that is stabilized through a mesomeric effect. The subsequent addition of **5** to **2**, acting as a Michael acceptor yields **6**, which undergoes protonation to afford **7**. The decarboxylation of **7** is the key step of the reaction, allowing for the removal of the activating group. The protonation of the enolate **8** thus obtained yields of the desired chromanone **3** bearing an α,α-disubstituted azlactone moiety. It was anticipated that the use of a chiral Brønsted base **9** as a catalyst of such decarboxylative Michael reaction should afford access to enantio- and diastereomerically enriched products [73,74,75,76,77]. 

Herein, we present our studies on the application of the decarboxylative Michael reaction for the enantioselective synthesis of biologically relevant chromanones bearing an α,α-disubstituted azlactone moiety. The possibility to transform the azlactone ring into a protected α,α-disubstituted amino acid has also been demonstrated.

Initially the Michael reaction between azlactone **1a** and 4-chromone **2a** was performed (Table 1, entry 1). However, no reaction was observed. To our delight, the incorporation of a carboxylic acid moiety into the structure of the Michael acceptor **2b** resulted in the formation of the desired product **3a** when quinine **9a** was employed as a catalyst (Table 1, Entry 2). The reaction proceeded in a cascade manner, and the initial Michael addition was accompanied by the decarboxylative protonation. Disappointingly, while the diastereoselectivity of the process was good, its enantioselectivity was low. Therefore, a catalyst screening was performed using chromone-3-carboxylic acid **2b** as a model Michael acceptor (Table 1, Entries 2–7). Interestingly, the introduction of double H-bonding units into the structure of the cinchona alkaloid (catalysts **9b**–**f**) led to the improvement of reaction stereoselectivity. The best results were obtained when catalyst **9e** was used (Table 1, Entry 6) [78]. With the best catalyst identified, the solvent screening was initiated (Table 1, Entries 8–13). However, inferior results were obtained. Subsequently, the effect of concentration (Table 1, Entries 14,15), the relative ratio of reactants (Table 1, Entries 16,17) and temperature (Table 1, Entry 18) on the reaction outcome was evaluated. Disappointingly, no further improvement of the results was observed. Notably, the reaction proved readily scalable with comperable results obtained when 1 g of **2b** was used (Table 1, Entry 19).

**Scheme 3:**
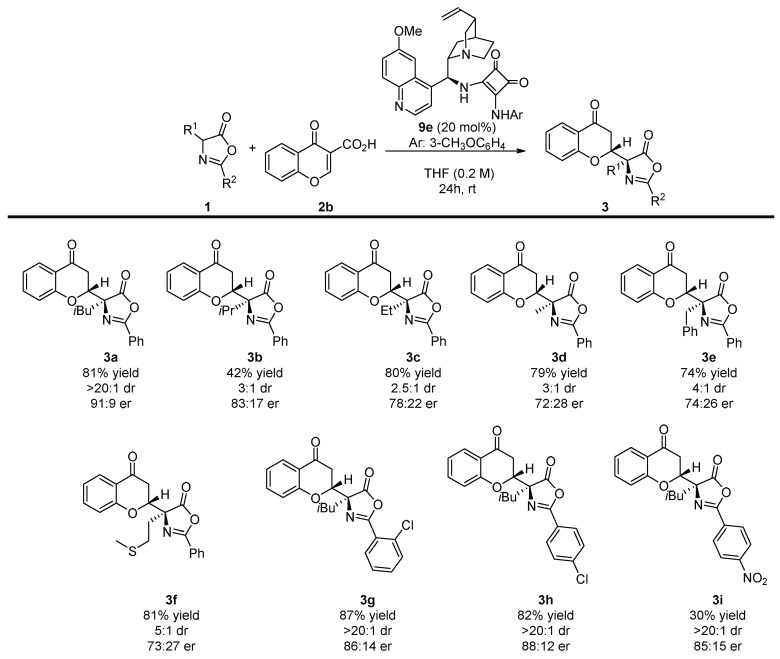
Decarboxylative enantioselective synthesis of chromanones **3** bearing an azlactone unit—α-substituted azlactones **1** scope. Reactions performed on a 0.1 mmol scale using **1** (1 equivalent) and **2b** (1 equivalent) in 0.2 mL of the solvent. For details see Supplementary Materials.

With the optimal reaction conditions established, the scope of the methodology was tested. Initially, various α-substituted azlactones **1** were employed in the developed decarboxylative Michael reaction (Scheme 3). In all of cases, the reaction proceeded with moderate-to-high yields. Disappointingly, regardless the size of the substituent in the α-position of the azlactone ring, lower diastereoselectivities were observed. Similarly, products **3b**–**f** were obtained with a deteriorated enantiomeric enrichment when compared with the model product **3a**. Interestingly, the reaction was successfully realized for azlactones **1g–i** bearing various R^2^ groups.

**Scheme 4:**
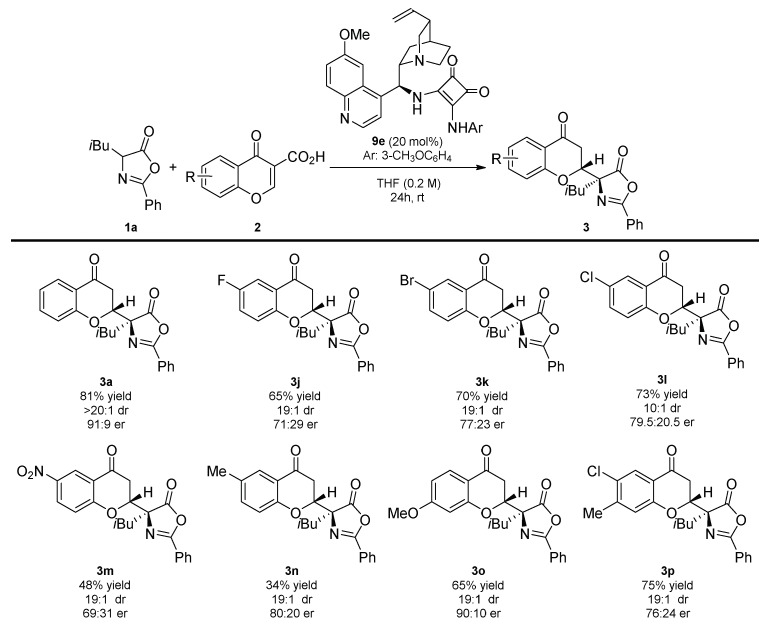
Decarboxylative enantioselective synthesis of chromanones **3** bearing an azlactone unit—chromone-3-carboxylic acids **4** scope. Reactions performed on a 0.1 mmol scale using **1a** (1 equivalent) and **2** (1 equivalent) in 0.2 mL of the solvent. For details see Supplementary Materials.

In the second part of the scope studies, the usefulness of various chromone-3-carboxylic acids **2** in the developed reaction was evaluated (Scheme 4). It was found that the diastereoselectivity of the decarboxylative Michael reaction was unbiased towards the electron properties and the position of the substituents on the aromatic ring in the corresponding chromone-3-carboxylic acid **2**. Both electron-poor and electron-rich substituents were possibly present in **2**, providing products **3** in good-to-high yields and excellent diastereoselectivity. Furthermore, the disubstitution pattern in **2h** was also well-tolerated, as shown in the decarboxylative Michael reaction leading to **3p**. In all of cases, the enantioselectivity of the process was lower than for the model reaction. 

**Scheme 5:**
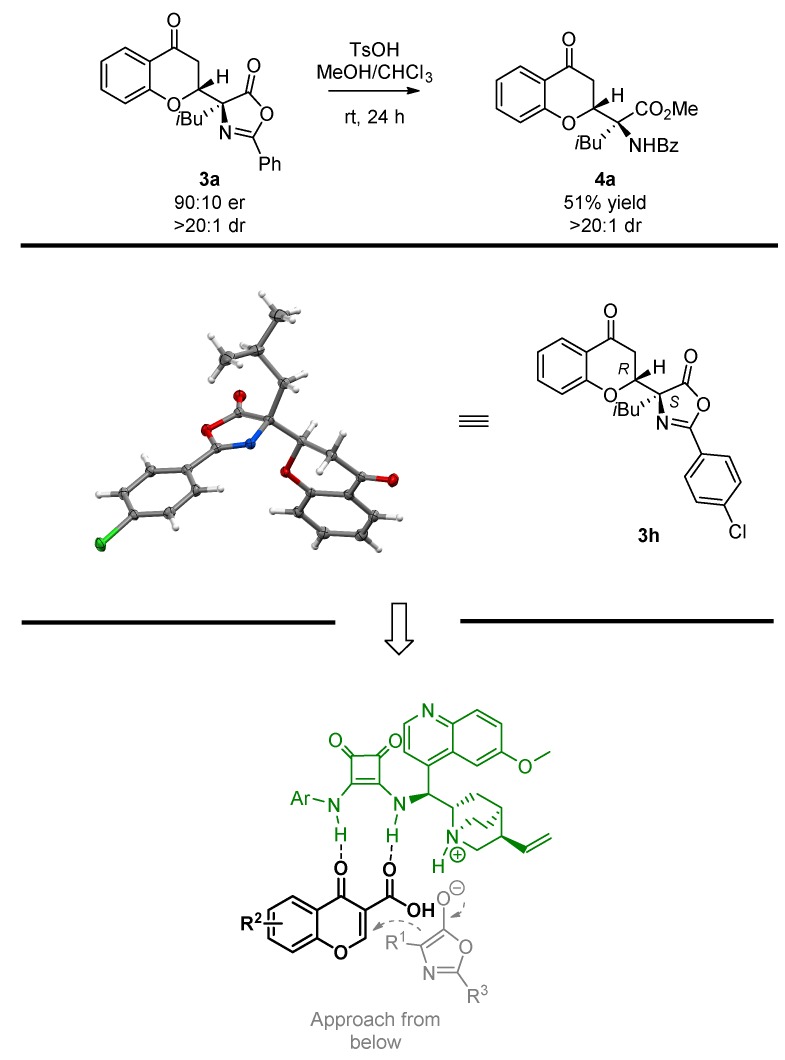
Azlactone-ring opening in **3a**, relative configuration assignment, and transition state proposal.

To demonstrate the usefulness of the Michael adduct **3a** obtained for the synthesis of α,α-disubstituted amino acids, the azlactone-ring-opening was attempted (Scheme 5, top). It was found that under acidic conditions, product **4a** bearing an α,α-disubstituted amino acid moiety was obtained in a 51% yield. Notably, the reaction proceeded with the full preservation of the stereochemical information introduced at the decarboxylative Michael addition step, as **4a** was obtained as single diastereoisomer. In the course of further studies, the absolute configuration of the Michael adduct **3h** was unambiguously established through single crystal X-ray analysis (Scheme 5, middle) [79]. Notably, the absolute configuration of the remaining products was established by analogy. Given the assignment performed, a transition state model rationalizing the observed stereochemistry was proposed (Scheme 5, bottom). It is postulated that the corresponding chromone-3-carboxylic acid **2** was recognized by the catalyst **9e** through the H-bonding interaction with its squaramide moiety. At the same time, azlactone **1** was deprotonated by the tertiary amine moiety present in the quinuclidine ring of **9e**. As the result of the ion pair formation between the protonated catalyst and the enolate obtained, the Michael addition occurred in a stereoselective fashion.

## 3. Conclusion

In conclusion, we have developed a novel, decarboxylative reaction between α-substituted azlactones and chromone-3-carboxylic acids leading to biologically relevant chromanones bearing an azlactone moiety. Its ring-opening realized under acidic conditions constitutes a facile route to protected α,α-disubstituted α-amino acid derivatives. The activation of the Michael acceptor through the introduction of a carboxylic acid moiety proved both necessary and a very convenient means to achieve the desired reactivity pathway.

## 4. Materials and Methods 

### 4.1. General Methods 

NMR spectra were acquired on a Bruker Ultra Shield 700 instrument (Bruker Corporation, Billerica, MA, USA), running at 700 MHz for ^1^H and 176 MHz for ^13^C, respectively. Chemical shifts (δ) were reported in ppm relative to residual solvent signals (CDCl_3_: 7.26 ppm for ^1^H NMR, 77.16 ppm for ^13^C NMR). Mass spectra were recorded on a Bruker Maxis Impact spectrometer using electrospray (ES+) ionization (referenced to the mass of the charged species). Analytical thin layer chromatography (TLC) was performed using pre-coated aluminum-backed plates (Merck Kieselgel 60 F254) and visualized by the ultraviolet irradiation or I_2_ stain. Unless otherwise noted, analytical grade solvents and commercially available reagents were used without further purification. For flash chromatography (FC), silica gel (Silica gel 60, 230–400 mesh, Merck, Darmstadt, Germany) was used. The enantiomeric ratio (er) of the products were determined either by ultra performance convergence chromatography (UPC2) using Daicel Chiralpak IA and IG columns as chiral stationary phases or by chiral stationary phase HPLC (Daicel Chiralpak IF column). Azlactones **1** were synthetized according to the literature procedure [80]. Chromone-3-carboxylic acids **2** were prepared from the corresponding 2-hydroxyacetophenones following the literature procedure [81]. 

### 4.2. General Procedure 

An ordinary screw-cap vial was charged with a magnetic stirring bar, the corresponding chromone-3-carboxylic acid **2** (0.1 mmol, 1 equivalent), THF (0.2 mL), catalyst **9e** (0.02 mmol, 0.2 equivalent), and the corresponding azlactone **1** (0.1 mmol, 1 equivalent). The reaction mixture was stirred at room temperature and monitored by ^1^H NMR spectroscopy. After the complete consumption of the carboxylic acid **2**, the mixture was directly subjected to FC on silica gel (hexane:ethyl acetate 15:1 or 10:1) to afford pure product **3**.

**(*S*)*-*4-Isobutyl-((*R*)*-*4-oxochroman-2-yl)-2-phenyl-1,3-oxazol-5(4*H*)-one** (**3a**) pure product was isolated by flash chromatography on silica gel (hexane:ethyl acetate 10:1) as yellow crystals (m.p. 124–126 °C) in an 81% yield (29.8 mg), dr > 20:1. Major diastereoisomer: IR (film): 3072, 1813, 1691, 1652, 1603, 1463, 1307, 1223, 995, 884, 760 cm^−1^. ^1^H NMR (700 MHz, CDCl_3_) δ 8.03 (d, *J* = 7.8 Hz, 2H), 7.84 (t, *J* = 10.2 Hz, 1H), 7.59 (t, *J* = 7.4 Hz, 1H), 7.49 (t, *J* = 7.6 Hz, 2H), 7.39 (t, *J* = 7.7 Hz, 1H), 6.97 (t, *J* = 7.5 Hz, 1H), 6.87 (t, *J* = 8.4 Hz, 1H), 4.74 (dd, *J* = 13.0, 2.9 Hz, 1H), 3.22 (dd, *J* = 16.9, 13.0 Hz, 1H), 2.92 (dd, *J* = 16.9, 2.9 Hz, 1H), 1.93 (dd, *J* = 13.8, 6.3 Hz, 1H), 1.84 (dd, *J* = 13.8, 6.5 Hz, 1H), 1.66–1.60 (m, 1H), 0.91 (d, *J* = 6.7 Hz, 3H), 0.90 (d, *J* = 6.6 Hz, 3H). ^13^C NMR (176 MHz, CDCl_3_) δ 191.1, 178.6, 161.7, 160.6, 136.3, 133.2, 129.0 (2C), 128.4 (2C), 126.9, 125.7, 122.0, 121.0, 118.0, 80.7, 75.9, 41.1, 38.1, 24.8, 24.0, 23.5. HRMS: Calculated for [C_22_H_21_NO_4_+H^+^]: 364.1543, found: 364.154. The er was determined by HPLC using a Chiralpak IF column [hexane/i-PrOH (80:20)]; flow rate 1.0 mL/min; τmajor = 6.3 min; τminor = 10.0 min, (91:9 er).

**((*R*)*-*4-Oxochroman-2-yl)-2-phenyl-(*S*)*-*4-isopropan-2-yl-1,3-oxazol-5(4*H*)-one** (**3b**) pure product was isolated by flash chromatography on silica gel (hexane:ethyl acetate 10:1) as yellow crystals (m.p. 121–122 °C) in a 42% yield (14.7 mg), dr = 3:1. IR (film): 2922, 1813, 1691, 1653, 1605, 1463, 1229, 1180, 993, 881, 763, 700 cm^−1^. ^1^H NMR (700 MHz, CDCl_3_) Major diastereoisomer: δ 8.09 (d, *J* = 7.3 Hz, 2H), 7.86 (t, *J* = 7.9 Hz, 1H), 7.63 (t, *J* = 7.5 Hz, 1H), 7.53 (t, *J* = 7.8 Hz, 2H), 7.41 (t, *J* = 8.7 Hz, 1H), 7.02 (t, *J* = 7.5 Hz, 1H), 6.84 (d, *J* = 8.4 Hz, 1H), 4.90 (dd, *J* = 14.0, 2.6 Hz, 1H), 3.31 (dd, *J* = 16.8, 14.0 Hz, 1H), 2.77 (dd, *J* = 16.8, 2.6 Hz, 1H), 2.44 (hept, *J* = 6.9 Hz, 1H), 1.13 (d, *J* = 6.8 Hz, 3H), 0.96 (d, *J* = 6.6 Hz, 3H). Minor diastereoisomer: δ 8.03 (d, *J* = 7.3 Hz, 2H), 7.86 (t, *J* = 7.9 Hz, 1H), 7.59 (t, *J* = 7.5 Hz, 1H), 7.49 (t, *J* = 7.8 Hz, 2H), 7.40 (t, *J* = 7.6 Hz, 1H), 6.99 (t, *J* = 7.4 Hz, 1H), 6.88 (d, *J* = 8.4 Hz, 1H), 5.03 (dd, *J* = 13.2, 2.8 Hz, 1H), 3.21 (dd, *J* = 16.9, 13.3 Hz, 1H), 2.86 (dd, *J* = 16.9, 2.9 Hz, 1H), 2.38 (hept, *J* = 6.9 Hz, 1H), 1.18 (d, *J* = 7.0 Hz, 3H), 0.95 (d, *J* = 6.5 Hz, 3H). ^13^C NMR (176 MHz, CDCl_3_) Major diastereoisomer: δ 191.1, 177.6, 162.2, 160.7, 136.2, 133.2, 129.0 (2C), 128.4 (2C), 127.0, 125.7, 122.2, 121.1, 118.2, 78.6, 78.1, 37.0, 31.6, 17.2, 16.7. Minor diastereoisomer: δ 191.1, 177.0, 161.9, 160.9, 136.3, 133.1, 128.9 (2C), 128.3 (2C), 127.0, 125.6, 122.0, 121.1, 118.1, 78.6, 78.4, 37.9, 31.3, 17.3, 15.7. HRMS: Calculated for [C_21_H_19_NO_4_+H^+^]: 350.1387, found: 350.1380. The er was determined by UPC2 using a chiral Chiralpack IA column gradient from 100% CO_2_ up to 40%; i-PrOH, 2.5 mL/min; detection wavelength = 245 nm; τ_major_ = 2.52 min, τ_minor_ = 2.60 min, (83:17 er).

**(*S*)-4-Ethyl-(*R*)-4-oxochroman-2-yl-2-phenyl-1,3-oxazol-5(4*H*)-one** (**3c**) pure product was isolated by flash chromatography on silica gel (hexane:ethyl acetate 15:1) as yellow crystals (m.p. 122–124 °C) in an 80% yield (26.8 mg), dr = 2.5:1. IR (film): 2960, 1816, 1691, 1654, 1604, 1463, 1227, 1152, 994, 882, 761 cm^−1^. ^1^H NMR (700 MHz, CDCl_3_) Major diastereoisomer: δ 8.04 (d, *J* = 7.7 Hz, 2H), 7.85 (d, *J* = 8.1 Hz, 1H), 7.60 (t, *J* = 7.4 Hz, 1H), 7.50 (t, *J* = 7.7 Hz, 2H), 7.40 (t, *J* = 7.2 Hz, 1H), 6.99 (t, *J* = 7.5 Hz, 1H), 6.87 (d, *J* = 8.4 Hz, 1H), 4.83 (dd, *J* = 13.2, 2.8 Hz, 1H), 3.25 (dd, *J* = 16.8, 13.2 Hz, 1H), 2.90 (dd, *J* = 16.9, 2.8 Hz, 1H), 1.97 (q, *J* = 7.3 Hz, 2H), 0.90 (t, *J* = 7.4 Hz, 3H). Minor diastereoisomer: ^1^H NMR (700 MHz, CDCl_3_) δ 8.07 (d, *J* = 7.7 Hz, 2H), 7.87 (d, *J* = 8.0 Hz, 1H), 7.63 (t, *J* = 7.5 Hz, 1H), 7.54 (t, *J* = 7.7 Hz, 2H), 7.44 (t, *J* = 7.2 Hz, 1H), 7.03 (t, *J* = 7.5 Hz, 1H), 6.90 (d, *J* = 8.4 Hz, 1H), 4.78 (dd, *J* = 13.8, 2.6 Hz, 1H), 3.21 (dd, *J* = 16.7, 13.8 Hz, 1H), 2.75 (dd, *J* = 16.7, 2.6 Hz, 1H), 2.13 (dq, *J* = 14.6, 7.4 Hz, 1H), 2.05 (dq, *J* = 14.4, 7.3 Hz, 1H), 0.94 (t, *J* = 7.4 Hz, 3H). ^13^C NMR (176 MHz, CDCl_3_) Major diastereoisomer: δ 191.0, 177.9, 162.0, 160.6, 136.2, 133.2, 128.9 (2C), 128.4 (2C), 126.9, 125.6, 122.0, 121.0, 118.0, 80.0, 76.8, 38.0, 26.0, 7.7. Minor diastereoisomer: δ 190.8, 177.3, 162.0, 160.6, 136.2, 133.2, 129.0 (2C), 128.4 (2C), 127.0, 125.5, 122.2, 121.0, 118.2, 79.1, 76.0, 37.5, 26.4, 8.0. HRMS: Calculated for [C_20_H_17_NO_4_+H^+^]: 336.1230, found: 336.1239. The er was determined by UPC2 using a chiral Chiralpack IG column gradient from 100% CO_2_ up to 40%; i-PrOH, 2.5 mL/min; detection wavelength = 245 nm; τ_major_ = 2.66 min, τ_minor_ = 3.40 min, (78:22 er).

**(*S*)*-*4-Methyl-2-((*R*)*-*4-oxochroman-2-yl)-2-phenyl-1,3-oxazol-5(4*H*)-one** (**3d**) pure product was isolated by flash chromatography on silica gel (hexane:ethyl acetate 15:1) as yellow crystals (m.p. 112–113 °C) in a 79% yield (25.4 mg) dr = 3:1. IR (film): 3058, 1817, 1692, 1650, 1607, 1464, 1307, 1225, 1154, 993, 880, 762 cm^−1^. ^1^H NMR (700 MHz, CDCl_3_) Major diastereoisomer: δ 8.03 (d, *J* = 7.8 Hz, 2H), 7.86 (d, *J* = 7.9 Hz, 1H), 7.59 (t, *J* = 7.4 Hz, 1H), 7.49 (t, *J* = 7.7 Hz, 2H), 7.40 (t, *J* = 7.3 Hz, 1H), 6.99 (t, *J* = 7.5 Hz, 1H), 6.87 (d, *J* = 8.4 Hz, 1H), 4.80 (dd, *J* = 13.1, 2.8 Hz, 1H), 3.25 (dd, *J* = 16.8, 13.2 Hz, 1H), 2.93 (dd, *J* = 16.8, 2.9 Hz, 1H), 1.56 (s, 3H). Minor diastereoisomer: δ 8.05 (d, *J* = 7.7 Hz, 2H), 7.86 (d, *J* = 8.8 Hz, 1H), 7.62 (t, *J* = 7.4 Hz, 1H), 7.52 (t, *J* = 7.7 Hz, 2H), 7.43 (t, *J* = 7.8 Hz, 1H), 7.02 (t, *J* = 7.5 Hz, 1H), 6.90 (d, *J* = 8.4 Hz, 1H), 4.73 (dd, *J* = 13.9, 2.3 Hz, 1H), 3.21 (dd, *J* = 16.5, 14.1 Hz, 1H), 2.75 (dd, *J* = 16.7, 2.4 Hz, 1H), 1.64 (s, 3H). ^13^C NMR (176 MHz, CDCl_3_) Major diastereoisomer: δ 190.9, 178.5, 161.7, 160.6, 136.3, 133.2, 128.9 (2C), 128.3 (2C), 126.9, 125.7, 122.1, 121.0, 118.0, 80.2, 72.0, 37.6, 19.5. Minor diastereoisomer: δ 190.8, 177.6, 161.9, 160.5, 136.3, 133.3, 129.0 (2C), 128.3 (2C), 127.0, 125.6, 122.2, 120.9, 118.2, 79.5, 71.3, 37.4, 19.9. HRMS: Calculated for [C_19_H_15_NO_4_+H^+^]: 322.1074, found: 322.1077. The er was determined by UPC2 using a chiral Chiralpack IA column gradient from 100% CO_2_ up to 40%; i-PrOH, 2.5 mL/min; detection wavelength = 245 nm; τ_major_ =2.58 min, τ_mino_r = 2.79 min, (72:28 er).

**(*S*)*-*4-Benzyl-(*R*)*-*4-oxochroman-2-yl-2-phenyl-1,3-oxazol-5(4*H*)-one** (**3e**) pure product was isolated by flash chromatography on silica gel (hexane:ethyl acetate 15:1) as yellow crystals (m.p. 124–126 °C) in a 74% yield (29.4 mg), dr = 4:1. Major diastereoisomer: IR (film): 3033, 1817, 1688, 1652, 1603, 1459, 1299, 1225, 1106, 993, 764, 696 cm^−1^. ^1^H NMR (700 MHz, CDCl_3_) δ 7.88–7.86 (m, 3H), 7.54 (t, *J* = 7.5 Hz, 1H), 7.44–7.40 (m, 3H), 7.20–7.15 (m, 5H), 7.01 (t, *J* = 7.3 Hz, 1H), 6.90 (d, *J* = 8.3 Hz, 1H), 4.96 (dd, *J* = 13.2, 2.9 Hz, 1H), 3.33 (dd, *J* = 16.7, 13.2 Hz, 1H), 3.26 (d, *J* = 13.2 Hz, 1H), 3.16 (d, *J* = 13.2 Hz, 1H), 3.00 (dd, *J* = 16.8, 2.9 Hz, 1H). ^13^C NMR (176 MHz, CDCl_3_) δ 190.8, 177.0, 161.8, 160.6, 136.3, 133.0, 132.8, 130.4 (2C), 128.8 (2C), 128.5 (2C), 128.2 (2C), 127.8, 127.0, 125.5, 122.1, 121.1, 118.1, 79.9, 77.4, 39.0, 38.2. HRMS: calculated for [C_25_H_19_NO_4_+H^+^]: 398.1387, found: 398.1381. The er was determined by UPC2 using a chiral Chiralpack IA column gradient from 100% CO_2_ up to 40%; i-PrOH, 2.5 mL/min; detection wavelength = 245 nm; τ_major_ = 3.29 min, τ_minor_ = 4.04 min, (74:26 er).

**(*S*)*-*4-(2-(Methylthio)ethyl)-2-((*R*)*-*4-oxochroman-2-yl)-2-phenyl-1,3-oxazol-5(4*H*)-one** (**3f**) pure product was isolated by flash chromatography on silica gel (hexane:ethyl acetate 15:1) as colorless solid (m.p. 146–148 °C) in an 81% yield (30.8 mg), dr = 5:1. Major diastereoisomer: IR (film): 2957, 1818, 32 4 1651, 1603, 1459, 1297, 1225, 993, 893, 762, 696 cm^−1^. ^1^H NMR (700 MHz, CDCl_3_) δ 8.03 (d, *J* = 7.8 Hz, 2H), 7.86 (d, *J* = 7.7 Hz, 1H), 7.60 (t, *J* = 7.4 Hz, 1H), 7.50 (t, *J* = 7.7 Hz, 2H), 7.40 (t, *J* = 7.8 Hz, 1H), 6.99 (t, *J* = 7.5 Hz, 1H), 6.89 (d, *J* = 8.4 Hz, 1H), 4.81 (dd, *J* = 13.0, 2.9 Hz, 1H), 3.22 (dd, *J* = 16.8, 13.0 Hz, 1H), 2.91 (dd, *J* = 16.8, 3.0 Hz, 1H), 2.48 (ddd, *J* = 13.1, 9.6, 4.8 Hz, 1H), 2.39 (ddd, *J* = 13.1, 10.0, 6.7 Hz, 1H), 2.30–2.21 (m, 2H), 2.08 (s, 3H). ^13^C NMR (176 MHz, CDCl_3_) δ 190.7, 177.8, 162.6, 160.5, 136.3, 133.3, 128.9 (2C), 128.4 (2C), 127.0, 125.5, 122.2, 121.0, 118.0, 80.0, 75.3, 38.1, 32.0, 28.2, 15.4. HRMS: calculated for [C_21_H_19_NO_4_S+H^+^]: 382.1108, found: 382.1109. The er was determined by UPC2 using a chiral Chiralpack IA column gradient from 100% CO_2_ up to 40%; i-PrOH, 2.5 mL/min; detection wavelength = 245 nm; τ_major_ = 2.95 min, τ_minor_ = 3.22 min, (73:27 er).

**(*S*)*-*4-Isobutyl-(2-chlorophenyl)-((*R*)*-*4-oxochroman-2-yl)-2-1,3-oxazol-5(4*H*)-one** (**3g**) pure product was isolated by flash chromatography on silica gel (hexane:ethyl acetate 20:1) as yellow oil in an 87% yield (34.5 mg), dr = >20:1. Major diastereoisomer: IR (film): 3074, 1815, 1690, 1652, 1605, 1579, 1467, 1256, 1228, 995, 884, 762, 735 cm^−1^. ^1^H NMR (700 MHz, CDCl_3_) δ 7.85 (dt, *J* = 7.8, 1.6 Hz, 1H), 7.79 (dd, *J* = 7.8, 1.8 Hz, 1H), 7.55–7.49 (m, 1H), 7.47 (tt, *J* = 8.0, 1.4 Hz, 1H), 7.42 (ddd, *J* = 8.7, 5.2, 1.8 Hz, 1H), 7.37 (t, *J* = 7.6 Hz, 1H), 7.00 (t, *J* = 7.5 Hz, 1H), 6.91 (dd, *J* = 8.5, 2.9 Hz, 1H), 4.75 (dd, *J* = 13.2, 2.9 Hz, 1H), 3.29–3.15 (m, 1H), 2.93 (dd, *J* = 16.9, 2.9 Hz, 1H), 1.97 (dd, *J* = 13.8, 5.9 Hz, 1H), 1.85 (dd, *J* = 13.8, 6.9 Hz, 1H), 1.72 (dt, *J* = 13.1, 6.6 Hz, 1H), 0.95 (ddd, *J* = 19.0, 6.7, 1.7 Hz, 6H). ^13^C NMR (176 MHz, CDCl_3_) δ 190.6, 178.0, 160.4, 160.4, 136.2, 134.0, 133.0, 131.4, 131.2, 126.8, 126.8, 125.3, 122.0, 120.8, 117.8, 80.4, 75.8, 40.7, 38.0, 24.6, 23.9, 23.1. HRMS: Calculated for [C_22_H_20_ClNO_4_+H^+^]: 398, 1154, found: 398.1135. The er was determined by UPC2 using a chiral Chiralpack IA column gradient from 100% CO2 up to 40%; i-PrOH, 2.5 mL/min; detection wavelength = 245 nm; τ_major_ = 2.70 min, τ_minor_ = 3.20 min, (86:14 er).

**(*S*)*-*4-Isobutyl-(4-chlorophenyl)-((*R*)*-*4-oxochroman-2-yl)-2-1,3-oxazol-5(4*H*)-one** (**3h**) pure product was isolated by flash chromatography on silica gel (hexane:ethyl acetate 20:1) as colorless crystals (m.p. 188–190 °C) in an 82% yield (32.6 mg), dr = >20:1. Major diastereoisomer: IR (film): 3076, 1816, 1691, 1652, 1605, 1579, 1463, 1278, 1227, 994, 897, 761, 734 cm^−1^. ^1^H NMR (700 MHz, CDCl_3_) δ 7.95 (d, *J* = 8.7 Hz, 2H), 7.83 (dd, *J* = 7.8, 1.8 Hz, 1H), 7.46 (d, *J* = 8.7 Hz, 2H), 7.38 (ddd, *J* = 8.7, 7.2, 1.8 Hz, 1H), 6.97 (ddd, *J* = 8.0, 7.2, 1.0 Hz, 1H), 6.86 (dd, *J* = 8.4, 0.9 Hz, 1H), 4.75 (dd, *J* = 12.7, 3.0 Hz, 1H), 3.19 (dd, *J* = 16.8, 12.7 Hz, 1H), 2.92 (dd, *J* = 16.9, 3.0 Hz, 1H), 1.87 (ddd, *J* = 57.0, 13.9, 6.4 Hz, 2H), 1.61 (dt, *J* = 13.1, 6.6 Hz, 1H), 0.90 (t, *J* = 6.5 Hz, 6H). ^13^C NMR (176 MHz, CDCl_3_) δ 190.6, 178.1, 160.8, 160.3, 139.5, 136.1, 129.5 (2C), 129.2 (2C), 126.8, 124.0, 121.9, 120.9, 117.8, 80.5, 76.0, 40.9, 37.9, 24.7, 23.8, 23.3. HRMS: Calculated for [C_22_H_20_ClNO_4_+H^+^]: 398.1154, found: 398.1165. The er was determined by UPC2 using a chiral Chiralpack IG column gradient from 100% CO_2_ up to 40%; i-PrOH, 2.5 mL/min; detection wavelength = 245 nm; τ_major_ = 2.57 min, τ_minor_ = 3.40 min, (88:12 er).

**(*S*)*-*4-Isobutyl-2-(4-nitrophenyl)((*R*)*-*4-oxochroman-2-yl)-1,3-oxazol-5(4*H*)-one** (**3i**) pure product was isolated by flash chromatography on silica gel (hexane:ethyl acetate 15:1) as yellow oil in a 30% yield (12.2 mg), dr = >20:1. Major diastereoisomer: IR (film): 3074, 1815, 1690, 1652, 1605, 1552, 1467, 1256, 1228, 995, 762, 736 cm^−1^. ^1^H NMR (700 MHz, CDCl_3_) δ 8.34 (d, *J* = 8.9 Hz, 2H), 8.31–8.13 (m, 2H), 7.85 (dd, *J* = 7.9, 1.7 Hz, 1H), 7.39 (ddd, *J* = 8.8, 7.2, 1.8 Hz, 1H), 6.99 (ddd, *J* = 8.0, 7.1, 1.0 Hz, 1H), 6.86 (dd, *J* = 8.4, 1.0 Hz, 1H), 4.79 (dd, *J* = 12.5, 3.1 Hz, 1H), 3.21 (dd, *J* = 16.9, 12.5 Hz, 1H), 2.96 (dd, *J* = 16.9, 3.1 Hz, 1H), 1.96 (dd, *J* = 13.9, 6.3 Hz, 1H), 1.87 (dd, *J* = 14.0, 6.4 Hz, 1H), 1.62 (dt, *J* = 13.1, 6.5 Hz, 1H), 0.91 (dd, *J* = 6.6, 0.9 Hz, 6H). ^13^C NMR (176 MHz, CDCl_3_) δ 190.32, 177.43, 160.16, 160.07, 150.48, 136.17, 130.96, 129.25, 126.82, 123.96, 122.11, 120.91, 117.71, 80.50, 76.42, 40.90, 37.88, 24.72, 23.78, 23.34. HRMS: Calculated for [C_22_H_20_N_2_O_6_+H^+^]: 409.1394, found: 409.1402. The er was determined by UPC2 using a chiral Chiralpack IG column gradient from 100% CO_2_ up to 40%; i-PrOH, 2.5 mL/min; detection wavelength = 245 nm; τ_major_ = 3.01 min, τ_minor_ = 4.00 min, (85:15 er).

**(*S*)*-*4-Isobutyl-(6-fluoro-(*R*)*-*4-oxochroman-2-yl)-2-phenyl-1,3-oxazol-5(4*H*)-one** (**3j**) pure product was isolated by flash chromatography on silica gel (hexane:ethyl acetate 15:1) as yellow oil in a 65% yield (24.8 mg), dr = 19:1. Major diastereoisomer: IR (film): 3073, 1818, 1702, 1648, 1478, 1218, 878, 773, 699 cm^−1^. ^1^H NMR (700 MHz, CDCl^3^) δ 8.03 (d, *J* = 7.7 Hz, 2H), 7.60 (t, *J* = 7.4 Hz, 1H), 7.52–7.46 (m, 3H), 7.10 (ddd, *J* = 9.1, 7.7, 3.2 Hz, 1H), 6.86 (dd, *J* = 9.1, 4.1 Hz, 1H), 4.72 (dt, *J* = 8.6, 4.3 Hz, 1H), 3.20 (dd, *J* = 17.0, 12.9 Hz, 1H), 2.93 (dd, *J* = 17.0, 2.9 Hz, 1H), 1.91 (dd, *J* = 13.8, 6.2 Hz, 1H), 1.83 (dd, *J* = 13.8, 6.5 Hz, 1H), 1.66–1.59 (m, 1H), 0.91 (d, *J* = 6.7 Hz, 3H), 0.89 (d, *J* = 6.6 Hz, 3H). ^13^C NMR (176 MHz, CDCl_3_) δ 190.3 (d, *J* = 1.5 Hz), 178.5, 161.8, 157.6 (d, *J* = 242.9 Hz), 156.8 (d, *J* = 1.6 Hz), 133.3, 129.0 (2C), 128.3 (2C), 125.6, 123.7 (d, *J* = 24.6 Hz), 121.5 (d, *J* = 6.6 Hz), 119.7 (d, *J* = 7.4 Hz), 112.0 (d, *J* = 23.5 Hz), 80.9, 75.9, 41.1, 37.9, 24.8, 24.0, 23.5. HRMS: Calculated for [C_22_H_20_FNO_4_+H^+^]: 382.1449, found: 382.1449. The er was determined by UPC2 using a chiral Chiralpack IA column gradient from 100% CO_2_ up to 40%; i-PrOH, 2.5 mL/min; detection wavelength = 245 nm; τ_major_ = 2.15 min, τ_minor_ = 2.51 min, (71:29 er).

**(6-Bromo-(*R*)*-*4-oxochroman-2-yl)-(*S*)*-*4-isobutyl-2-phenyl-1,3-oxazol-5(4*H*)-one** (**3k**) pure product was isolated by flash chromatography on silica gel (hexane:ethyl acetate 15:1) as yellow solid (m.p. 142–144 °C) in a 70% yield (30.9 mg), dr = 19:1. Major diastereoisomer: IR (film): 2958, 1817, 1696, 1651, 1598, 1464, 1415, 1270, 1221, 884, 753, 702 cm^−1^. ^1^H NMR (700 MHz, CDCl_3_) δ 8.01 (d, *J* = 7.7 Hz, 2H), 7.94 (d, *J* = 2.4 Hz, 1H), 7.60 (t, *J* = 7.4 Hz, 1H), 7.49 (t, *J* = 7.7 Hz, 2H), 7.44 (dd, *J* = 8.8, 2.4 Hz, 1H), 6.77 (d, *J* = 8.8 Hz, 1H), 4.74 (dd, *J* = 12.5, 3.0 Hz, 1H), 3.20 (dd, *J* = 17.0, 12.6 Hz, 1H), 2.94 (dd, *J* = 17.0, 3.0 Hz, 1H), 1.91 (dd, *J* = 13.8, 6.2 Hz, 1H), 1.83 (dd, *J* = 13.8, 6.5 Hz, 1H), 1.63 (hept, *J* = 6.5 Hz, 1H), 0.91 (d, *J* = 6.7 Hz, 3H), 0.89 (d, *J* = 6.7 Hz, 3H). ^13^C NMR (176 MHz, CDCl_3_) δ 189.7, 178.4, 161.9, 159.4, 138.8, 133.3, 129.4, 129.0 (2C), 128.4 (2C), 125.5, 122.3, 120.0, 114.8, 80.9, 75.9, 41.1, 37.8, 24.8, 24.0, 23.5. HRMS: Calculated for [C_22_H_20_BrNO^4^+H^+^]: 442.0648, found: 442.0644. The er was determined by UPC2 using a chiral Chiralpack IG column gradient from 100% CO_2_ up to 40%; i-PrOH, 2.5 mL/min; detection wavelength = 245 nm; τ_major_ = 2.76 min, τ_minor_ = 3.25 min, (77:23 er).

**6-Chloro-((*S*)-4-isobutyl-(*R*)-4-oxochroman-2-yl)-2-phenyl-1,3-oxazol-5(4*H*)-one** (**3l**) pure product was isolated by flash chromatography on silica gel (hexane:ethyl acetate 15:1) as yellow solid (m.p. 118–120 °C) in a 73% yield (29.0 mg), dr = 10:1. Major diastereoisomer: IR (film): 3070, 1816, 1702, 1648, 1478, 1212, 878, 773, 699 cm^−1^. ^1^H NMR (700 MHz, CDCl_3_) δ 8.01 (dd, *J* = 8.4, 1.3 Hz, 2H), 7.94 (d, *J* = 2.5 Hz, 1H), 7.63–7.56 (m, 1H), 7.54–7.47 (m, 2H), 7.44 (dd, *J* = 8.8, 2.5 Hz, 1H), 6.78 (d, *J* = 8.8 Hz, 1H), 4.74 (dd, *J* = 12.6, 3.1 Hz, 1H), 3.20 (dd, *J* = 17.0, 12.5 Hz, 1H), 2.94 (dd, *J* = 17.0, 3.1 Hz, 1H), 1.91 (dd, *J* = 13.8, 6.3 Hz, 1H), 1.83 (dd, *J* = 13.8, 6.5 Hz, 1H), 1.63 (dt, *J* = 13.1, 6.6 Hz, 1H), 0.90 (dd, *J* = 9.5, 6.7 Hz, 6H). ^13^C NMR (176 MHz, CDCl_3_) δ 189.5, 178.2, 161.7, 159.3, 138.7, 133.2, 129.2, 128.8 (2C), 128.2 (2C), 125.4, 122.2, 119.9, 114.7, 80.8, 75.8, 41.0, 37.6, 24.7, 23.8, 23.4. HRMS: Calculated for [C_22_H_20_ClNO_4_+H^+^]: 398.1154, found: 398.1163. The er was determined by UPC2 using a chiral Chiralpack IA column gradient from 100% CO_2_ up to 40%; i-PrOH, 2.5 mL/min; detection wavelength = 245 nm; τmajor = 2.81 min, τminor = 3.26 min, (79.5:20.5 er).

**(*S*)*-*4-Isobutyl-(6-nitro-(*R*)*-*4-oxochroman-2-yl)-2-phenyl-1,3-oxazol-5(4*H*)-one** (**3m**) pure product was isolated by flash chromatography on silica gel (hexane:ethyl acetate 15:1) as yellow solid (m.p. 188–190 °C) in a 48% yield (19.6 mg), dr = 19:1. Major diastereoisomer: IR (film): 2922, 1819, 1710, 1605, 1585, 1469, 1275, 1233, 1183,1043, 906, 778, 665 cm^−1^. ^1^H NMR (700 MHz, CDCl_3_) δ 8.72 (d, *J* = 2.8 Hz, 1H), 8.20 (dd, *J* = 9.2, 2.8 Hz, 1H), 8.09–7.94 (m, 2H), 7.59 (d, *J* = 7.5 Hz, 1H), 7.51–7.42 (m, 2H), 7.00 (d, *J* = 9.1 Hz, 1H), 4.89 (dd, *J* = 11.5, 3.5 Hz, 1H), 3.28 (dd, *J* = 17.1, 11.5 Hz, 1H), 3.07 (dd, *J* = 17.1, 3.5 Hz, 1H), 1.97–1.78 (m, 2H), 1.67–1.57 (m, 1H), 0.91 (dd, *J* = 7.8, 6.6 Hz, 6H). ^13^C NMR (176 MHz, CDCl_3_) δ 188.4, 177.9, 164.0, 162.0, 133.4, 130.3, 128.9, 128.7, 128.4, 128.2, 125.1, 123.1, 120.6, 119.1, 81.4, 75.8, 41.0, 37.4, 24.7, 23.8, 23.33, 22.4. HRMS: Calculated for [C_22_H_20_N_2_O_6_+H^+^]: 409.1394, found: 409.1382. The er was determined by UPC2 using a chiral Chiralpack IG column gradient from 100% CO_2_ up to 40%; i-PrOH, 2.5 mL/min; detection wavelength = 245 nm; τ_major_ = 3.07 min, τ_minor_ = 3.34 min, (69:31 er).

**(*S*)*-*4-Isobutyl-(6-methyl-(*R*)*-*4-oxochroman-2-yl)-2-phenyl-1,3-oxazol-5(4*H*)-one** (**3n**) pure product was isolated by flash chromatography on silica gel (hexane:ethyl acetate 15:1) as yellow solid (m.p. 102–106 °C) in a 34% yield (12.8 mg), dr = 19:1. Major diastereoisomer: IR (film): 3067, 1819, 1725, 1688, 1651,1558, 1450, 1076, 955, 778, 753 cm^−1^. ^1^H NMR (700 MHz, CDCl_3_) δ 8.03 (d, *J* = 7.6 Hz, 2H), 7.63 (d, *J* = 6.0 Hz, 1H), 7.59 (t, *J* = 7.4 Hz, 1H), 7.49 (t, *J* = 7.7 Hz, 2H), 7.20 (dd, *J* = 8.5, 1.9 Hz, 1H), 6.77 (t, *J* = 8.4 Hz, 1H), 4.70 (dd, *J* = 13.0, 2.8 Hz, 1H), 3.19 (dd, *J* = 16.8, 13.0 Hz, 1H), 2.89 (dd, *J* = 16.9, 2.8 Hz, 1H), 2.25 (s, 3H), 1.92 (dd, *J* = 13.9, 6.2 Hz, 1H), 1.83 (dd, *J* = 13.9, 6.5 Hz, 1H), 1.63 (hept, *J* = 6.5 Hz, 1H), 0.91 (d, *J* = 6.7 Hz, 3H), 0.89 (d, *J* = 6.6 Hz, 3H). ^13^C NMR (176 MHz, CDCl_3_) δ 191.4, 178.7, 161.6, 158.7, 137.3, 133.2, 131.6, 128.9 (2C), 128.4 (2C), 126.5, 125.7, 120.6, 117.8, 80.7, 76.0, 41.1, 38.2, 24.8, 24.0, 23.5, 20.5. HRMS: Calculated for [C_23_H_23_NO_4_+H+]: 378.1700, found: 378.1698. The er was determined by UPC2 using a chiral Chiralpack IA column gradient from 100% CO_2_ up to 40%; i-PrOH, 2.5 mL/min; detection wavelength = 245 nm; τ_major_ = 2.43 min, τ_minor_ = 3.11 min, (80:20 er).

**(*S*)*-*4-Isobutyl-(7-methoxy-(*R*)*-*4-oxochroman-2-yl)-2-phenyl-1,3-oxazol-5(4*H*)-one** (**3o**) pure product was isolated by flash chromatography on silica gel (hexane:ethyl acetate 15:1) as yellow solid (m.p. 160–161 °C) in a 65% yield (25.5 mg), dr = 19:1. Major diastereoisomer: IR (film): 3071, 1819, 1684, 1651,1582, 1486, 1281, 1214, 1099, 883, 700, 561 cm^−1^. ^1^H NMR (700 MHz, CDCl_3_) δ 8.04 (d, *J* = 7.3 Hz, 2H), 7.59 (t, *J* = 7.4 Hz, 1H), 7.49 (t, *J* = 7.8 Hz, 2H), 7.26 (d, *J* = 3.3 Hz, 1H), 6.99 (dd, *J* = 9.1, 3.2 Hz, 1H), 6.81 (d, *J* = 9.1 Hz, 1H), 4.69 (dd, *J* = 13.1, 2.8 Hz, 1H), 3.75 (s, 3H), 3.19 (dd, *J* = 16.9, 13.1 Hz, 1H), 2.90 (dd, *J* = 16.9, 2.9 Hz, 1H), 1.92 (dd, *J* = 13.9, 6.2 Hz, 1H), 1.83 (dd, *J* = 13.9, 6.5 Hz, 1H), 1.66–1.60 (m, 1H), 0.91 (d, *J* = 6.7 Hz, 2H), 0.89 (d, *J* = 6.6 Hz, 2H). ^13^C NMR (176 MHz, CDCl_3_) δ 191.2, 178.6, 161.6, 155.3, 154.6, 133.1, 128.9 (2C), 128.4 (2C), 125.8, 125.3, 120.9, 119.3, 107.4, 80.8, 75.9, 55.9, 41.2, 38.1, 24.8, 24.0, 23.5. HRMS: Calculated for [C_23_H_23_NO_5_+H^+^]: 394.1649, found: 394.1645. The er was determined by UPC2 using a chiral Chiralpack IA column gradient from 100% CO_2_ up to 40%; i-PrOH, 2.5 mL/min; detection wavelength = 245 nm; τ_major_ = 2.58 min, τ_minor_ = 3.11 min, (90:10 er).

**(*S*)*-*4-Isobutyl-(6-chloro-7-methyl-(*R*)*-*4-oxochroman-2-yl)-2-phenyl-1,3-oxazol-5(4*H*)-one** (**3p**) pure product was isolated by flash chromatography on silica gel (hexane:ethyl acetate 15:1) as yellow oil in a 75% yield (30.8 mg), dr = 19:1. Major diastereoisomer: IR (film): 3065, 1819, 1691, 1652, 1611, 1408, 1319, 1154, 873, 703 cm^–1^. ^1^H NMR (700 MHz, CDCl_3_) δ 8.02 (d, *J* = 7.6 Hz, 2H), 7.78 (s, 1H), 7.59 (t, *J* = 7.4 Hz, 1H), 7.49 (t, *J* = 7.8 Hz, 2H), 6.78 (s, 1H), 4.71 (dd, *J* = 12.7, 3.0 Hz, 1H), 3.17 (dd, *J* = 17.0, 12.7 Hz, 1H), 2.90 (dd, *J* = 17.0, 3.0 Hz, 1H), 2.27 (s, 2H), 1.90 (dd, *J* = 13.9, 6.2 Hz, 1H), 1.82 (dd, *J* = 13.9, 6.5 Hz, 1H), 1.63 (hept, *J* = 6.6 Hz, 1H), 0.91 (d, *J* = 6.7 Hz, 3H), 0.89 (d, *J* = 6.6 Hz, 3H). ^13^C NMR (176 MHz, CDCl_3_) δ 189.7, 178.5, 161.8, 158.8, 145.4, 133.2, 129.0 (2C), 128.4 (2C), 128.3, 126.6, 125.6, 120.1, 120.0, 81.0, 75.9, 41.1, 37.8, 24.8, 24.0, 23.5, 20.8. HRMS: Calculated for [C_23_H_22_ClNO_4_+H^+^]: 412.1310, found: 412.1319. The er was determined by UPC2 using a chiral Chiralpack IA column gradient from 100% CO_2_ up to 40%; i-PrOH, 2.5 mL/min; detection wavelength = 245 nm; τ_major_ = 2.65 min, τ_mino_r = 2.87 min, (76:24 er).

### 4.3. Synthesis of Methyl 2-Benzamido-4-Methyl-2-(4-Oxochroman-2-yl)Pentanoate (**4a**) 

An ordinary screw-cap vial was charged with a magnetic stirring bar, the chromone **3a** (0.05 mmol, 17 mg), MeOH (200 μL), and CHCl_3_ (100 μL). Then toluenesulphonic acid monohydrate (0.1 mmol, 19 mg) was added, and the reaction mixture was stirred for 1.5 h at 40 °C. The product was isolated using flash chromatography in an eluent gradient (starting from hexane:ethyl acetate—10:1 to hexane:ethyl acetate—5:1), giving **4a** as a yellow oil in a 51% yield (10.0 mg), dr = >20:1 dr. Major diastereoisomer: IR (film): 3405, 3064, 1819, 1738, 1691, 1669, 1579, 1464, 1442, 1304, 1224, 1030, 765, 710 cm^−1^. ^1^H NMR (700 MHz, CDCl_3_) δ 7.85 (d, *J* = 7.9 Hz, 1H), 7.82 (d, *J* = 7.9 Hz, 2H), 7.53 (t, *J* = 7.5 Hz, 1H), 7.49 (bs, 1H), 7.46 (t, *J* = 7.8 Hz, 3H), 7.01 (t, *J* = 7.5 Hz, 1H), 6.95 (d, *J* = 7.9 Hz, 1H), 5.03 (dd, *J* = 14.0, 2.4 Hz, 1H), 3.87 (s, 3H), 3.05 (dd, *J* = 14.1, 5.0 Hz, 1H), 3.02 (dd, *J* = 16.9, 2.5 Hz, 1H), 2.85 (dd, *J* = 16.9, 14.1 Hz, 1H), 1.95 (dd, *J* = 14.6, 7.3 Hz, 1H), 1.71–1.63 (m, 1H), 0.96 (d, *J* = 7.3 Hz, 3H), 0.86 (d, *J* = 7.2 Hz, 3H). ^13^C NMR (176 MHz, CDCl_3_) δ 191.5, 173.2, 166.8, 161.0, 135.9, 134.8, 131.8, 128.7 (2C), 127.0 (2C), 127.0, 121.8, 121.0, 117.7, 81.4, 67.2, 53.3, 39.3, 37.6, 24.7, 23.7, 22.3. HRMS: Calculated for [C_23_H_25_NO_5_+H^+^]: 396.1805, found: 396.1812.

## Figures and Tables

**Table 1:**
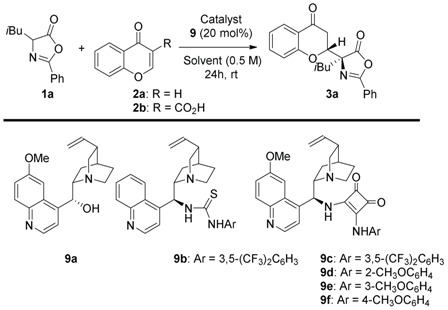
Decarboxylative enantioselective synthesis of chromanone **3** bearing an azlactone unit—optimization studies ^a^.

	Solvent	2/9	Conv. (Yield) [%] ^b^	dr ^c^	er ^d^	ee ^d^ [%]
1	THF	**2a/9a**	<5	n.d.	n.d.	n.d.
2	THF	**2b/9a**	>95	3:1	55:45	10
3	THF	**2b/9b**	>95	5:1	75:25	50
4	THF	**2b/9c**	>95	3:1	84:16	68
5	THF	**2b/9d**	>95	10:1	84:16	68
6	THF	**2b/9e**	>95 (81)	>20:1	91:9	82
7	THF	**2b/9f**	>95	>20:1	79:21	58
8	CH_2_Cl_2_	**2b/9e**	>95	5:1	81:19	62
9	Toluene	**2b/9e**	>95	5:1	91:9	82
10	1,4-Dioxane	**2b/9e**	>95	10:1	87:13	74
11	CPME	**2b/9e**	>95	4:1	84:16	68
12	Et_2_O	**2b/9e**	>95	6:1	70:30	40
13	2-MeTHF	**2b/9e**	>95	15:1	89:11	78
14 ^e^	THF	**2b/9e**	>95	20:1	90:10	80
15 ^f^	THF	**2b/9e**	>95	20:1	90:10	80
16 ^g^	THF	**2b/9e**	>95	10:1	70:30	40
17 ^h^	THF	**2b/9e**	>95	20:1	84:16	68
18 ^i^	THF	**2b/9e**	>95	20:1	84:16	68
19 ^j^	THF	**2b/9e**	>95 (76)	>20:1	91:9	82

^a^ Reactions performed on a 0.1 mmol scale using **1a** (1 equivalent) and **2** (1 equivalent) in 0.2 mL of the solvent. ^b^ Determined by ^1^H NMR of a crude reaction mixture. In parenthesis isolated yields are given. ^c^ Determined by ^1^H NMR of a crude reaction mixture. ^d^ Determined by a chiral stationary phase UPC2. ^e^ Reaction performed in 0.1 mL of THF. ^f^ Reaction performed in 1.0 mL of THF. ^g^ Reaction performed using **1a** (1.5 equivalent). ^h^ Reaction performed using 1.5 equivalent of **2b**. ^i^ Reaction performed at 0 °C. ^j^ Reaction performed using 1 gram of **2b**.

## Supplementary Materials

The following are available online. Copies of ^1^H and ^13^C spectra of all obtained compounds. Copies of HPLC and UPC data. Crystal structure details.
