# Supplementary material for: Enantioselective Synthesis of Chromanones Bearing an α,α-Disubstituted α-Amino Acid Moiety via Decarboxylative Michael Reaction
Source: Molecules. 2019 Jul 15;24(14):2565. doi: 10.3390/molecules24142565 (PMC6681191; doi:10.3390/molecules24142565)
Supplement: Supplementary file 1 [file molecules-24-02565-s001.pdf]

Enantioselective synthesis of chromanones bearing an α,α-disubstituted α-amino acid moiety via decarboxylative Michael reaction

Jan Bojanowski[a], Lesław Sieroń [b] Anna Albrecht*,[a]

[a] Institute of Organic Chemistry
Łódź University of Technology
90-924 Łódź, Poland

[b] Institute of General and Ecological Chemistry
Łódź University of Technology
90-924 Łódź, Poland

e-mail: anna.albrecht@p.lodz.pl

Contents

1.	General methods	S2
2.	Decarboxylative enantioselective synthesis of chromanones **3** bearing an azlactone unit – general procedure	S3
3.	Synthesis of methyl 2-benzamido-4-methyl-2-(4-oxochroman-2-yl)pentanoate (**4a**)	S10
4.	Crystal and X-ray data for (*S*)-4-Isobutyl-(4-chlorophenyl)-((*R*)-4-oxochroman-2-yl)-2-1,3-oxazol-5(4*H*)-one (**3h**)	S11
5.	References	S 13
6.	NMR data	S 14
7.	HPLC traces	S 31

1. General methods

NMR spectra were acquired on a Bruker Ultra Shield 700 instrument, running at 700 MHz for ^{1}H and 176 MHz for ^{13}C, respectively. Chemical shifts (δ) are reported in ppm relative to residual solvent signals ($CDCl_3$: 7.26 ppm for ^{1}H NMR, 77.16 ppm for ^{13}C NMR). Mass spectra were recorded on a Bruker Maxis Impact spectrometer using electrospray (ES+) ionization (referenced to the mass of the charged species). Analytical thin layer chromatography (TLC) was performed using pre-coated aluminum-backed plates (Merck Kieselgel 60 F254) and visualized by ultraviolet irradiation or I_2 stain. Unless otherwise noted, analytical grade solvents and commercially available reagents were used without further purification. For flash chromatography (FC) silica gel (Silica gel 60, 230-400 mesh, Fluka). The enantiomeric ratio (er) of the products were determined either by Ultra Performance Convergence Chromatography (UPC2) using Daicel Chiralpak IA and IG columns as chiral stationary phases or by chiral stationary phase HPLC (Daicel Chiralpak IF column). Azlactones **1** were synthetized according to the literature procedure[1]. Chromone-3-carboxylic acids **2** were prepared from the corresponding 2-hydroxyacetophenones following the literature procedure.[2]

2. General procedure

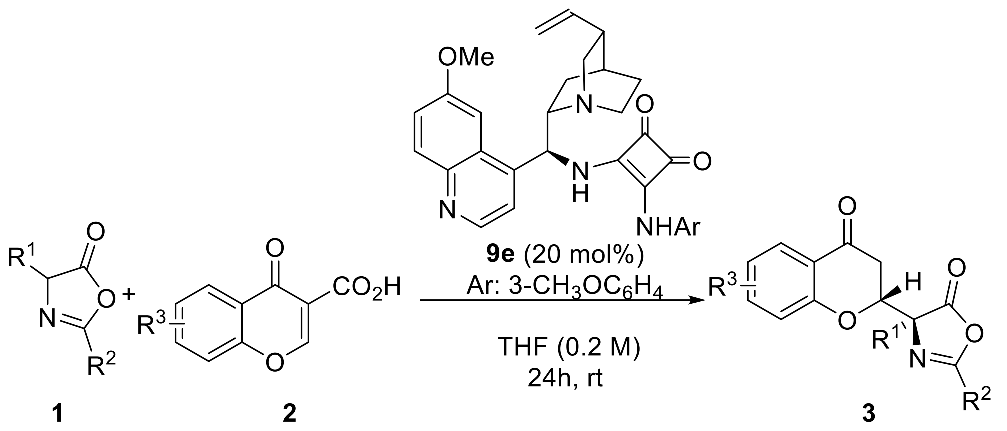

An ordinary screw-cap vial was charged with a magnetic stirring bar, the corresponding chromone-3-carboxylic acid **2** (0.1 mmol, 1 equiv), THF (0.2 mL), catalyst **9e** (0.02mmol, 0.2 equiv) and the corresponding azlactone **1** (0.1 mmol, 1 equiv). The reaction mixture was stirred at room temperature and monitored by 1H NMR spectroscopy. After complete consumption of the carboxylic acid **2** the mixture was directly subjected to FC on silica gel (hexane:ethyl acetate 15:1 or 10:1) to afford pure product **3**.

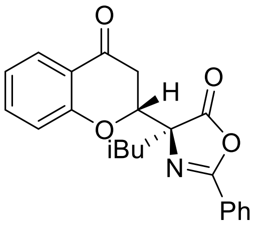

(*S*)-4-Isobutyl-((*R*)-4-oxochroman-2-yl)-2-phenyl-1,3-oxazol-5(4*H*)-one (**3a**) pure product was isolated by flash chromatography on silica gel (hexane:ethyl acetate 10:1) as yellow crystals (m.p. 124-126 °C) in 81% yield (29.8 mg), dr > 20:1. Major diastereoisomer:

IR (film): 3072, 1813, 1691, 1652, 1603, 1463, 1307, 1223, 995, 884, 760 cm^{-1}.

^{1}H NMR (700 MHz, $CDCl_3$) δ 8.03 (d, *J* = 7.8 Hz, 2H), 7.84 (t, *J* = 10.2 Hz, 1H), 7.59 (t, *J* = 7.4 Hz, 1H), 7.49 (t, *J* = 7.6 Hz, 2H), 7.39 (t, *J* = 7.7 Hz, 1H), 6.97 (t, *J* = 7.5 Hz, 1H), 6.87 (t, *J* = 8.4 Hz, 1H), 4.74 (dd, *J* = 13.0, 2.9 Hz, 1H), 3.22 (dd, *J* = 16.9, 13.0 Hz, 1H), 2.92 (dd, *J* = 16.9, 2.9 Hz, 1H), 1.93 (dd, *J* = 13.8, 6.3 Hz, 1H), 1.84 (dd, *J* = 13.8, 6.5 Hz, 1H), 1.66 – 1.60 (m, 1H), 0.91 (d, *J* = 6.7 Hz, 3H), 0.90 (d, *J* = 6.6 Hz, 3H).

^{13}C NMR (176 MHz, $CDCl_3$) δ 191.1, 178.6, 161.7, 160.6, 136.3, 133.2, 129.0 (2C), 128.4 (2C), 126.9, 125.7, 122.0, 121.0, 118.0, 80.7, 75.9, 41.1, 38.1, 24.8, 24.0, 23.5.

HRMS: calculated for [$C_{22}H_{21}NO_4$+H^+]: 364.1543, found: 364.154.

The er was determined by The er was determined by HPLC using a Chiralpak IF column [hexane/i-PrOH (80:20)]; flow rate 1.0 mL/min; τmajor =6.3 min; τminor = 10.0 min, (91:9 er).

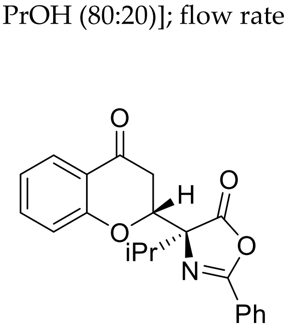

((*R*)-4-Oxochroman-2-yl)-2-phenyl-(*S*)-4-isopropan-2-yl-1,3-oxazol-5(4*H*)-one (**3b**) pure product was isolated by flash chromatography on silica gel (hexane:ethyl acetate 10:1) as yellow crystals (m.p. 121-122 °C) in 42% yield (14.7 mg), dr = 2:1.

IR (film): 2922, 1813, 1691, 1653, 1605, 1463, 1229, 1180, 993, 881, 763, 700 cm^{-1}.

^{1}H NMR (700 MHz, $CDCl_3$) Major diastereoisomer: δ 8.09 (d, *J* = 7.3 Hz, 2H), 7.86 (t, *J* = 7.9 Hz, 1H), 7.63 (t, *J* = 7.5 Hz, 1H), 7.53 (t, *J* = 7.8 Hz, 2H), 7.41 (t, *J* = 8.7 Hz, 1H), 7.02 (t, *J* = 7.5 Hz, 1H), 6.84 (d, *J* = 8.4 Hz, 1H), 4.90 (dd, *J* = 14.0, 2.6 Hz, 1H), 3.31 (dd, *J* = 16.8, 14.0 Hz, 1H), 2.77 (dd, *J* = 16.8, 2.6 Hz, 1H), 2.44 (hept, *J* = 6.9 Hz, 1H), 1.13 (d, *J* = 6.8 Hz, 3H), 0.96 (d, *J* = 6.6 Hz, 3H). Minor

diastereoisomer: δ 8.03 (d, *J* = 7.3 Hz, 2H), 7.86 (t, *J* = 7.9 Hz, 1H), 7.59 (t, *J* = 7.5 Hz, 1H), 7.49 (t, *J* = 7.8 Hz, 2H), 7.40 (t, *J* = 7.6 Hz, 1H), 6.99 (t, *J* = 7.4 Hz, 1H), 6.88 (d, *J* = 8.4 Hz, 1H), 5.03 (dd, *J* = 13.2, 2.8 Hz, 1H), 3.21 (dd, *J* = 16.9, 13.3 Hz, 1H), 2.86 (dd, *J* = 16.9, 2.9 Hz, 1H), 2.38 (hept, *J* = 6.9 Hz, 1H), 1.18 (d, *J* = 7.0 Hz, 3H), 0.95 (d, *J* = 6.5 Hz, 3H).

^{13}C NMR (176 MHz, $CDCl_3$) Major diastereoisomer: δ 191.1, 177.6, 162.2, 160.7, 136.2, 133.2, 129.0 (2C), 128.4 (2C), 127.0, 125.7, 122.2, 121.1, 118.2, 78.6, 78.1, 37.0, 31.6, 17.2, 16.7. Minor diastereoisomer: δ 191.1, 177.0, 161.9, 160.9, 136.3, 133.1, 128.9 (2C), 128.3 (2C), 127.0, 125.6, 122.0, 121.1, 118.1, 78.6, 78.4, 37.9, 31.3, 17.3, 15.7.

HRMS: calculated for [$C_{21}H_{19}NO_4$+H^+]: 350.1387, found: 350.1380.

The er was determined by UPC2 using a chiral Chiralpack IA column gradient from 100% CO_2 up to 40%; i-PrOH, 2.5 mL/min; detection wavelength = 245 nm; τ_{major} = 2.52 min, τ_{minor} = 2.60 min, (83:17 er).

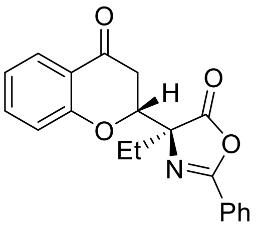

(*S*)-4-Ethyl-(*R*)-4-oxochroman-2-yl-2-phenyl-1,3-oxazol-5(4*H*)-one (**3c**) pure product was isolated by flash chromatography on silica gel (hexane:ethyl acetate 15:1) as yellow crystals (m.p. 122-124 °C) in 80% yield (26.8 mg), dr = 2.5:1.

IR (film): 2960, 1816, 1691, 1654, 1604, 1463, 1227, 1152, 994, 882, 761 cm^{-1}.

^{1}H NMR (700 MHz, $CDCl_3$) Major diastereoisomer: δ 8.04 (d, *J* = 7.7 Hz, 2H), 7.85 (d, *J* = 8.1 Hz, 1H), 7.60 (t, *J* = 7.4 Hz, 1H), 7.50 (t, *J* = 7.7 Hz, 2H), 7.40 (t, *J* = 7.2 Hz, 1H), 6.99 (t, *J* = 7.5 Hz, 1H), 6.87 (d, *J* = 8.4 Hz, 1H), 4.83 (dd, *J* = 13.2, 2.8 Hz, 1H), 3.25 (dd, *J* = 16.8, 13.2 Hz, 1H), 2.90 (dd, *J* = 16.9, 2.8 Hz, 1H), 1.97 (q, *J* = 7.3 Hz, 2H), 0.90 (t, *J* = 7.4 Hz, 3H). Minor diastereoisomer: ^{1}H NMR (700 MHz, $CDCl_3$) δ 8.07 (d, *J* = 7.7 Hz, 2H), 7.87 (d, *J* = 8.0 Hz, 1H), 7.63 (t, *J* = 7.5 Hz, 1H), 7.54 (t, *J* = 7.7 Hz, 2H), 7.44 (t, *J* = 7.2 Hz, 1H), 7.03 (t, *J* = 7.5 Hz, 1H), 6.90 (d, *J* = 8.4 Hz, 1H), 4.78 (dd, *J* = 13.8, 2.6 Hz, 1H), 3.21 (dd, *J* = 16.7, 13.8 Hz, 1H), 2.75 (dd, *J* = 16.7, 2.6 Hz, 1H), 2.13 (dq, *J* = 14.6, 7.4 Hz, 1H) , 2.05 (dq, *J* = 14.4, 7.3 Hz, 1H), 0.94 (t, *J* = 7.4 Hz, 3H).

^{13}C NMR (176 MHz, $CDCl_3$) Major diastereoisomer: δ 191.0, 177.9, 162.0, 160.6, 136.2, 133.2, 128.9 (2C), 128.4 (2C), 126.9, 125.6, 122.0, 121.0, 118.0, 80.0, 76.8, 38.0, 26.0, 7.7. Minor diastereoisomer: δ 190.8, 177.3, 162.0, 160.6, 136.2, 133.2, 129.0 (2C), 128.4 (2C), 127.0, 125.5, 122.2, 121.0, 118.2, 79.1, 76.0, 37.5, 26.4, 8.0.

HRMS: calculated for [$C_{20}H_{17}NO_4$+H^+]: 336.1230, found: 336.1239.

The er was determined by UPC2 using a chiral Chiralpack IG column gradient from 100% CO_2 up to 40%; i-PrOH, 2.5 mL/min; detection wavelength = 245 nm; τ_{major} = 2.66 min, τ_{minor} = 3.40 min, (78:22 er).

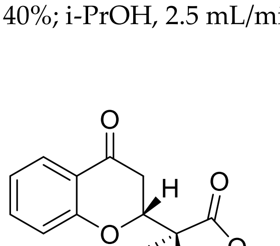

(*S*)-4-Methyl-2-((*R*)-4-oxochroman-2-yl)-2-phenyl-1,3-oxazol-5(4*H*)-one (**3d**) pure product was isolated by flash chromatography on silica gel (hexane : ethyl acetate 15:1) as yellow crystals (m.p. 112-113°C) in 79% yield (25.4 mg) dr = 3:1.

IR (film): 3058, 1817, 1692, 1650, 1607, 1464, 1307, 1225, 1154, 993, 880, 762 cm^{-1}.

^{1}H NMR (700 MHz, $CDCl_3$) Major diastereoisomer: δ 8.03 (d, *J* = 7.8 Hz, 2H), 7.86 (d, *J* = 7.9 Hz, 1H), 7.59 (t, *J* = 7.4 Hz, 1H), 7.49 (t, *J* = 7.7 Hz, 2H), 7.40 (t, *J* = 7.3 Hz, 1H), 6.99 (t, *J* = 7.5 Hz, 1H), 6.87 (d, *J* = 8.4 Hz, 1H), 4.80 (dd, *J* = 13.1, 2.8 Hz, 1H), 3.25 (dd, *J* = 16.8, 13.2 Hz, 1H), 2.93 (dd, *J* = 16.8, 2.9 Hz, 1H), 1.56 (s, 3H). Minor diastereoisomer: δ 8.05 (d, *J* = 7.7 Hz, 2H), 7.86 (d, *J* = 8.8 Hz, 1H), 7.62 (t, *J* = 7.4 Hz, 1H), 7.52 (t, *J* = 7.7 Hz, 2H), 7.43 (t, *J* = 7.8 Hz, 1H), 7.02

(t, *J* = 7.5 Hz, 1H), 6.90 (d, *J* = 8.4 Hz, 1H), 4.73 (dd, *J* = 13.9, 2.3 Hz, 1H), 3.21 (dd, *J* = 16.5, 14.1 Hz, 1H), 2.75 (dd, *J* = 16.7, 2.4 Hz, 1H), 1.64 (s, 3H).

^{13}C NMR (176 MHz, $CDCl_3$) Major diastereoisomer: δ 190.9, 178.5, 161.7, 160.6, 136.3, 133.2, 128.9 (2C), 128.3 (2C), 126.9, 125.7, 122.1, 121.0, 118.0, 80.2, 72.0, 37.6, 19.5. Minor diastereoisomer: δ 190.8, 177.6, 161.9, 160.5, 136.3, 133.3, 129.0 (2C), 128.3 (2C), 127.0, 125.6, 122.2, 120.9, 118.2, 79.5, 71.3, 37.4, 19.9.

HRMS: calculated for [$C_{19}H_{15}NO_4$+H^+]: 322.1074, found: 322.1077.

The er was determined by UPC2 using a chiral Chiralpack IA column gradient from 100% CO_2 up to 40%; i-PrOH, 2.5 mL/min; detection wavelength = 245 nm; τ_{major} =2.58 min, τ_{minor}r = 2.79 min, (72:28 er).

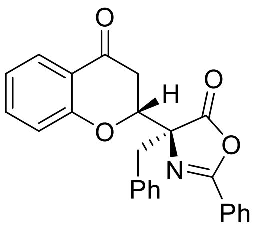

(*S*)-4-Benzyl-(*R*)-4-oxochroman-2-yl-2-phenyl-1,3-oxazol-5(4*H*)-one (**3e**) pure product was isolated by flash chromatography on silica gel (hexane:ethyl acetate 15:1) as yellow crystals (m.p. 124-126 °C) in 74% yield (29.4 mg), dr = 4:1. Major diastereoisomer:

IR (film): 3033, 1817, 1688, 1652, 1603, 1459, 1299, 1225, 1106, 993, 764, 696 cm^{-1}.

^{1}H NMR (700 MHz, $CDCl_3$) δ 7.88 – 7.86 (m, 3H), 7.54 (t, *J* = 7.5 Hz, 1H), 7.44 – 7.40 (m, 3H), 7.20 – 7.15 (m, 5H), 7.01 (t, *J* = 7.3 Hz, 1H), 6.90 (d, *J* = 8.3 Hz, 1H), 4.96 (dd, *J* = 13.2, 2.9 Hz, 1H), 3.33 (dd, *J* = 16.7, 13.2 Hz, 1H), 3.26 (d, *J* = 13.2 Hz, 1H), 3.16 (d, *J* = 13.2 Hz, 1H), 3.00 (dd, *J* = 16.8, 2.9 Hz, 1H).

^{13}C NMR (176 MHz, $CDCl_3$) δ 190.8, 177.0, 161.8, 160.6, 136.3, 133.0, 132.8, 130.4 (2C), 128.8 (2C), 128.5 (2C), 128.2 (2C) , 127.8, 127.0, 125.5, 122.1, 121.1, 118.1, 79.9, 77.4, 39.0, 38.2.

HRMS: calculated for [$C_{25}H_{19}NO_4$+H^+]: 398.1387, found: 398.1381.

The er was determined by UPC2 using a chiral Chiralpack IA column gradient from 100% CO_2 up to 40%; i-PrOH, 2.5 mL/min; detection wavelength = 245 nm; τ_{major} = 3.29 min, τ_{minor} = 4.04 min, (74:26 er).

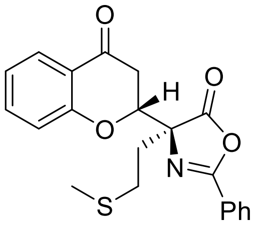

(*S*)-4-(2-(Methylthio)ethyl)-2-((*R*)-4-oxochroman-2-yl)-2-phenyl-1,3-oxazol-5(4*H*)-one (**3f**) pure product was isolated by flash chromatography on silica gel (hexane:ethyl acetate 15:1) as colorless solid (m.p. 146-148 °C) in 81% yield (30.8 mg), dr = 5:1. Major diastereoisomer:

IR (film): 2957, 1818, 32 4 1651, 1603, 1459, 1297, 1225, 993, 893, 762, 696 cm^{-1}.

^{1}H NMR (700 MHz, $CDCl_3$) δ 8.03 (d, *J* = 7.8 Hz, 2H), 7.86 (d, *J* = 7.7 Hz, 1H), 7.60 (t, *J* = 7.4 Hz, 1H), 7.50 (t, *J* = 7.7 Hz, 2H), 7.40 (t, *J* = 7.8 Hz, 1H), 6.99 (t, *J* = 7.5 Hz, 1H), 6.89 (d, *J* = 8.4 Hz, 1H), 4.81 (dd, *J* = 13.0, 2.9 Hz, 1H), 3.22 (dd, *J* = 16.8, 13.0 Hz, 1H), 2.91 (dd, *J* = 16.8, 3.0 Hz, 1H), 2.48 (ddd, *J* = 13.1, 9.6, 4.8 Hz, 1H), 2.39 (ddd, *J* = 13.1, 10.0, 6.7 Hz, 1H), 2.30 – 2.21 (m, 2H), 2.08 (s, 3H).

^{13}C NMR (176 MHz, $CDCl_3$) δ 190.7, 177.8, 162.6, 160.5, 136.3, 133.3, 128.9 (2C), 128.4 (2C), 127.0, 125.5, 122.2, 121.0, 118.0, 80.0, 75.3, 38.1, 32.0, 28.2, 15.4.

HRMS: calculated for [$C_{21}H_{19}NO_4S$+H^+]: 382.1108, found: 382.1109.

The er was determined by UPC2 using a chiral Chiralpack IA column gradient from 100% CO_2 up to 40%; i-PrOH, 2.5 mL/min; detection wavelength = 245 nm; τ_{major} = 2.95 min, τ_{minor} = 3.22 min, (73:27 er).

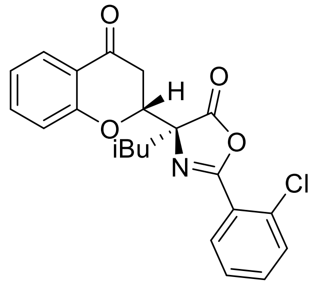

(*S*)-4-Isobutyl-(2-chlorophenyl)-((*R*)-4-oxochroman-2-yl)-2-1,3-oxazol-5(4*H*)-one (**3g**) pure product was isolated by flash chromatography on silica gel (hexane:ethyl acetate 20:1) as yellow oil in 87% yield (34.5 mg), dr = >20:1. Major diastereoisomer:

IR (film): 3074, 1815, 1690, 1652, 1605, 1579, 1467, 1256, 1228, 995, 884, 762, 735 cm^{-1}.

^{1}H NMR (700 MHz, $CDCl_3$) δ 7.85 (dt, *J* = 7.8, 1.6 Hz, 1H), 7.79 (dd, *J* = 7.8, 1.8 Hz, 1H), 7.55 – 7.49 (m, 1H), 7.47 (tt, *J* = 8.0, 1.4 Hz, 1H), 7.42 (ddd, *J* = 8.7, 5.2, 1.8 Hz, 1H), 7.37 (t, *J* = 7.6 Hz, 1H), 7.00 (t, *J* = 7.5 Hz, 1H), 6.91 (dd, *J* = 8.5, 2.9 Hz, 1H), 4.75 (dd, *J* = 13.2, 2.9 Hz, 1H), 3.29 – 3.15 (m, 1H), 2.93 (dd, *J* = 16.9, 2.9 Hz, 1H), 1.97 (dd, *J* = 13.8, 5.9 Hz, 1H), 1.85 (dd, *J* = 13.8, 6.9 Hz, 1H), 1.72 (dt, *J* = 13.1, 6.6 Hz, 1H), 0.95 (ddd, *J* = 19.0, 6.7, 1.7 Hz, 6H).

^{13}C NMR (176 MHz, $CDCl_3$) δ 190.6, 178.0, 160.4, 160.4, 136.2, 134.0, 133.0, 131.4, 131.2, 126.8, 126.8, 125.3, 122.0, 120.8, 117.8, 80.4, 75.8, 40.7, 38.0, 24.6, 23.9, 23.1.

HRMS: calculated for [$C_{22}H_{20}ClNO_4$+H^+]: 398,1154, found: 398.1135.

The er was determined by UPC2 using a chiral Chiralpack IA column gradient from 100% CO2 up to 40%; i-PrOH, 2.5 mL/min; detection wavelength = 245 nm; τ_{major} = 2.70 min, τ_{minor} = 3.20 min, (86:14 er).

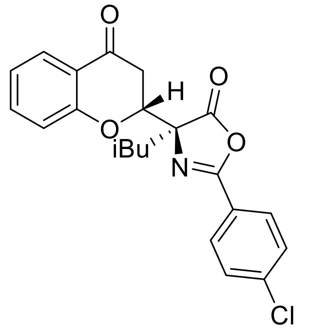

(*S*)-4-Isobutyl-(4-chlorophenyl)-((*R*)-4-oxochroman-2-yl)-2-1,3-oxazol-5(4*H*)-one (**3h**) pure product was isolated by flash chromatography on silica gel (hexane:ethyl acetate 20:1) as colorless crystals (m.p. 188-190 °C) in 82% yield (32.6 mg), dr = >20:1. Major diastereoisomer:

IR (film): 3076, 1816, 1691, 1652, 1605, 1579, 1463, 1278, 1227, 994, 897, 761, 734 cm^{-1}.

^{1}H NMR (700 MHz, $CDCl_3$) δ 7.95 (d, *J* = 8.7 Hz, 2H), 7.83 (dd, *J* = 7.8, 1.8 Hz, 1H), 7.46 (d, *J* = 8.7 Hz, 2H), 7.38 (ddd, *J* = 8.7, 7.2, 1.8 Hz, 1H), 6.97 (ddd, *J* = 8.0, 7.2, 1.0 Hz, 1H), 6.86 (dd, *J* = 8.4, 0.9 Hz, 1H), 4.75 (dd, *J* = 12.7, 3.0 Hz, 1H), 3.19 (dd, *J* = 16.8, 12.7 Hz, 1H), 2.92 (dd, *J* = 16.9, 3.0 Hz, 1H), 1.87 (ddd, *J* = 57.0, 13.9, 6.4 Hz, 2H), 1.61 (dt, *J* = 13.1, 6.6 Hz, 1H), 0.90 (t, *J* = 6.5 Hz, 6H).

^{13}C NMR (176 MHz, $CDCl_3$) δ 190.6, 178.1, 160.8, 160.3, 139.5, 136.1, 129.5 (2C), 129.2 (2C), 126.8, 124.0, 121.9, 120.9, 117.8, 80.5, 76.0, 40.9, 37.9, 24.7, 23.8, 23.3.

HRMS: calculated for [$C_{22}H_{20}ClNO_4$+H^+]: 398.1154, found: 398.1165.

The er was determined by UPC2 using a chiral Chiralpack IG column gradient from 100% CO_2 up to 40%; i-PrOH, 2.5 mL/min; detection wavelength = 245 nm; τ_{major} = 2.57 min, τ_{minor} = 3.40 min, (88:12 er).

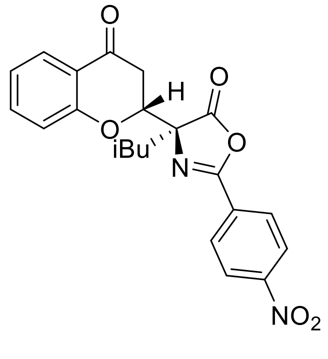

(*S*)-4-Isobutyl-2-(4-nitrophenyl)((*R*)-4-oxochroman-2-yl)-1,3-oxazol-5(4*H*)-one (**3i**) pure product was isolated by flash chromatography on silica gel (hexane:ethyl acetate 15:1) as yellow oil in 30% yield (12.2 mg), dr = >20:1. Major diastereoisomer:

IR (film): 3074, 1815, 1690, 1652, 1605, 1552, 1467, 1256, 1228, 995, 762, 736 cm^{-1}.

^{1}H NMR (700 MHz, $CDCl_3$) δ 8.34 (d, *J* = 8.9 Hz, 2H), 8.31 – 8.13 (m, 2H), 7.85 (dd, *J* = 7.9, 1.7 Hz, 1H), 7.39 (ddd, *J* = 8.8, 7.2, 1.8 Hz, 1H), 6.99 (ddd,

J = 8.0, 7.1, 1.0 Hz, 1H), 6.86 (dd, *J* = 8.4, 1.0 Hz, 1H), 4.79 (dd, *J* = 12.5, 3.1 Hz, 1H), 3.21 (dd, *J* = 16.9, 12.5 Hz, 1H), 2.96 (dd, *J* = 16.9, 3.1 Hz, 1H), 1.96 (dd, *J* = 13.9, 6.3 Hz, 1H), 1.87 (dd, *J* = 14.0, 6.4 Hz, 1H), 1.62 (dt, *J* = 13.1, 6.5 Hz, 1H), 0.91 (dd, *J* = 6.6, 0.9 Hz, 6H).

^{13}C NMR (176 MHz, $CDCl_3$) δ 190.32, 177.43, 160.16, 160.07, 150.48, 136.17, 130.96, 129.25, 126.82, 123.96, 122.11, 120.91, 117.71, 80.50, 76.42, 40.90, 37.88, 24.72, 23.78, 23.34.

HRMS: calculated for [$C_{22}H_{20}N_2O_6$+H^+]: 409.1394, found: 409.1402.

The er was determined by UPC2 using a chiral Chiralpack IG column gradient from 100% CO_2 up to 40%; i-PrOH, 2.5 mL/min; detection wavelength = 245 nm; τ_{major} = 3.01 min, τ_{minor} = 4.00 min, (85:15 er).

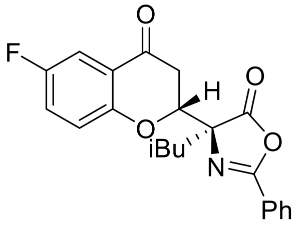

(*S*)-4-Isobutyl-(6-fluoro-(*R*)-4-oxochroman-2-yl)-2-phenyl-1,3-oxazol-5(4*H*)-one (3j) pure product was isolated by flash chromatography on silica gel (hexane:ethyl acetate 15:1) as yellow oil in 65% yield (24.8 mg), dr = 19:1. Major diastereoisomer:

IR (film): 3073, 1818, 1702, 1648, 1478, 1218, 878, 773, 699 cm^{-1}.

^{1}H NMR (700 MHz, CDCl^3) δ 8.03 (d, *J* = 7.7 Hz, 2H), 7.60 (t, *J* = 7.4 Hz, 1H), 7.52 – 7.46 (m, 3H), 7.10 (ddd, *J* = 9.1, 7.7, 3.2 Hz, 1H), 6.86 (dd, *J* = 9.1, 4.1 Hz, 1H), 4.72 (dt, *J* = 8.6, 4.3 Hz, 1H), 3.20 (dd, *J* = 17.0, 12.9 Hz, 1H), 2.93 (dd, *J* = 17.0, 2.9 Hz, 1H), 1.91 (dd, *J* = 13.8, 6.2 Hz, 1H), 1.83 (dd, *J* = 13.8, 6.5 Hz, 1H), 1.66 – 1.59 (m, 1H), 0.91 (d, *J* = 6.7 Hz, 3H), 0.89 (d, *J* = 6.6 Hz, 3H).

^{13}C NMR (176 MHz, $CDCl_3$) δ 190.3 (d, *J* = 1.5 Hz), 178.5, 161.8, 157.6 (d, *J* = 242.9 Hz), 156.8 (d, *J* = 1.6 Hz), 133.3, 129.0 (2C), 128.3 (2C), 125.6, 123.7 (d, *J* = 24.6 Hz), 121.5 (d, *J* = 6.6 Hz), 119.7 (d, *J* = 7.4 Hz), 112.0 (d, *J* = 23.5 Hz), 80.9, 75.9, 41.1, 37.9, 24.8, 24.0, 23.5.

HRMS: calculated for [$C_{22}H_{20}FNO_4$+H^+]: 382.1449, found: 382.1449.

The er was determined by UPC2 using a chiral Chiralpack IA column gradient from 100% CO_2 up to 40%; i-PrOH, 2.5 mL/min; detection wavelength = 245 nm; τ_{major} = 2.15 min, τ_{minor} = 2.51 min, (71:29 er).

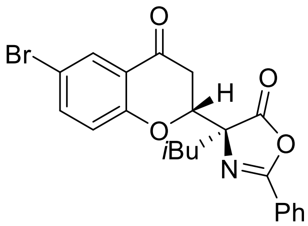

(6-Bromo-(*R*)-4-oxochroman-2-yl)-(*S*)-4-isobutyl-2-phenyl-1,3-oxazol-5(4*H*)-one (3k) pure product was isolated by flash chromatography on silica gel (hexane:ethyl acetate 15:1) as yellow solid (m.p. 142-144 ^{o}C) in 70% yield (30.9 mg), dr = 19:1. Major diastereoisomer:

IR (film): 2958, 1817, 1696, 1651, 1598, 1464, 1415, 1270, 1221, 884, 753, 702 cm^{-1}.

^{1}H NMR (700 MHz, $CDCl_3$) δ 8.01 (d, *J* = 7.7 Hz, 2H), 7.94 (d, *J* = 2.4 Hz, 1H), 7.60 (t, *J* = 7.4 Hz, 1H), 7.49 (t, *J* = 7.7 Hz, 2H), 7.44 (dd, *J* = 8.8, 2.4 Hz, 1H), 6.77 (d, *J* = 8.8 Hz, 1H), 4.74 (dd, *J* = 12.5, 3.0 Hz, 1H), 3.20 (dd, *J* = 17.0, 12.6 Hz, 1H), 2.94 (dd, *J* = 17.0, 3.0 Hz, 1H), 1.91 (dd, *J* = 13.8, 6.2 Hz, 1H), 1.83 (dd, *J* = 13.8, 6.5 Hz, 1H), 1.63 (hept, *J* = 6.5 Hz, 1H), 0.91 (d, *J* = 6.7 Hz, 3H), 0.89 (d, *J* = 6.7 Hz, 3H).

^{13}C NMR (176 MHz, $CDCl_3$) δ 189.7, 178.4, 161.9, 159.4, 138.8, 133.3, 129.4, 129.0 (2C), 128.4 (2C), 125.5, 122.3, 120.0, 114.8, 80.9, 75.9, 41.1, 37.8, 24.8, 24.0, 23.5.

HRMS: calculated for [$C_{22}H_{20}BrNO^4$+H^+]: 442.0648, found: 442.0644.

The er was determined by UPC2 using a chiral Chiralpack IG column gradient from 100% CO_2 up to 40%; i-PrOH, 2.5 mL/min; detection wavelength = 245 nm; τ_{major} = 2.76 min, τ_{minor} = 3.25 min, (77:23 er).

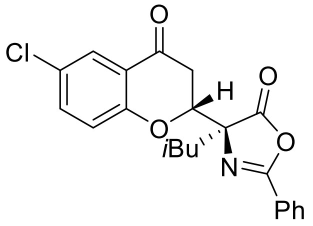

6-Chloro-((*S*)-4-isobutyl-(*R*)-4-oxochroman-2-yl)-2-phenyl-1,3-oxazol-5(4*H*)-one (**3l**) pure product was isolated by flash chromatography on silica gel (hexane:ethyl acetate 15:1) as yellow solid (m.p. 118 – 120 °C) in 73% yield (29.0 mg), dr = 10:1. Major diastereoisomer:

IR (film): 3070, 1816, 1702, 1648, 1478, 1212, 878, 773, 699 cm^{-1}.

^{1}H NMR (700 MHz, $CDCl_3$) δ 8.01 (dd, *J* = 8.4, 1.3 Hz, 2H), 7.94 (d, *J* = 2.5 Hz, 1H), 7.63 – 7.56 (m, 1H), 7.54 – 7.47 (m, 2H), 7.44 (dd, *J* = 8.8, 2.5 Hz, 1H), 6.78 (d, *J* = 8.8 Hz, 1H), 4.74 (dd, *J* = 12.6, 3.1 Hz, 1H), 3.20 (dd, *J* = 17.0, 12.5 Hz, 1H), 2.94 (dd, *J* = 17.0, 3.1 Hz, 1H), 1.91 (dd, *J* = 13.8, 6.3 Hz, 1H), 1.83 (dd, *J* = 13.8, 6.5 Hz, 1H), 1.63 (dt, *J* = 13.1, 6.6 Hz, 1H), 0.90 (dd, *J* = 9.5, 6.7 Hz, 6H).

^{13}C NMR (176 MHz, $CDCl_3$) δ 189.5, 178.2, 161.7, 159.3, 138.7, 133.2, 129.2, 128.8 (2C), 128.2 (2C), 125.4, 122.2, 119.9, 114.7, 80.8, 75.8, 41.0, 37.6, 24.7, 23.8, 23.4.

HRMS: calculated for [$C_{22}H_{20}ClNO_4$+H^+]: 398.1154, found: 398.1163.

The er was determined by UPC2 using a chiral Chiralpack IA column gradient from 100% CO_2 up to 40%; i-PrOH, 2.5 mL/min; detection wavelength = 245 nm; τmajor = 2.81 min, τminor = 3.26 min, (79.5:20.5 er).

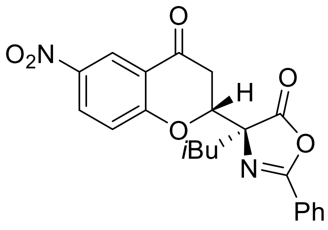

(*S*)-4-Isobutyl-(6-nitro-(*R*)-4-oxochroman-2-yl)-2-phenyl-1,3-oxazol-5(4*H*)-one (**3m**) pure product was isolated by flash chromatography on silica gel (hexane:ethyl acetate 15:1) as yellow solid (m.p. 188-190 °C) in 48% yield (19.6 mg), dr = 19:1. Major diastereoisomer:

IR (film): 2922, 1819, 1710, 1605, 1585, 1469, 1275, 1233, 1183,1043, 906, 778, 665 cm^{-1}.

^{1}H NMR (700 MHz, $CDCl_3$) δ 8.72 (d, *J* = 2.8 Hz, 1H), 8.20 (dd, *J* = 9.2, 2.8 Hz, 1H), 8.09 – 7.94 (m, 2H), 7.59 (d, *J* = 7.5 Hz, 1H), 7.51 – 7.42 (m, 2H), 7.00 (d, *J* = 9.1 Hz, 1H), 4.89 (dd, *J* = 11.5, 3.5 Hz, 1H), 3.28 (dd, *J* = 17.1, 11.5 Hz, 1H), 3.07 (dd, *J* = 17.1, 3.5 Hz, 1H), 1.97 – 1.78 (m, 2H), 1.67 – 1.57 (m, 1H), 0.91 (dd, *J* = 7.8, 6.6 Hz, 6H).

^{13}C NMR (176 MHz, $CDCl_3$) δ 188.4, 177.9, 164.0, 162.0, 133.4, 130.3, 128.9, 128.7, 128.4, 128.2, 125.1, 123.1, 120.6, 119.1, 81.4, 75.8, 41.0, 37.4, 24.7, 23.8, 23.33, 22.4.

HRMS: calculated for [$C_{22}H_{20}N_2O_6$+H^+]: 409.1394, found: 409.1382.

The er was determined by UPC2 using a chiral Chiralpack IG column gradient from 100% CO_2 up to 40%; i-PrOH, 2.5 mL/min; detection wavelength = 245 nm; τ_{major} = 3.07 min, τ_{minor} = 3.34 min, 69:31 er).

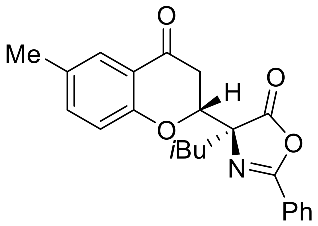

(*S*)-4-Isobutyl-(6-methyl-(*R*)-4-oxochroman-2-yl)-2-phenyl-1,3-oxazol-5(4*H*)-one (**3n**) pure product was isolated by flash chromatography on silica gel (hexane:ethyl acetate 15:1) as yellow solid (m.p. 102-106 °C) in 34% yield (12.8 mg), dr = 19:1. Major diastereoisomer:

IR (film): 3067, 1819, 1725, 1688, 1651,1558, 1450, 1076, 955, 778, 753 cm^{-1}.

^{1}H NMR (700 MHz, $CDCl_3$) δ 8.03 (d, *J* = 7.6 Hz, 2H), 7.63 (d, *J* = 6.0 Hz, 1H), 7.59 (t, *J* = 7.4 Hz, 1H), 7.49 (t, *J* = 7.7 Hz, 2H), 7.20 (dd, *J* = 8.5, 1.9 Hz, 1H), 6.77 (t, *J* = 8.4 Hz, 1H), 4.70 (dd, *J* = 13.0, 2.8 Hz, 1H), 3.19 (dd, *J* = 16.8, 13.0 Hz, 1H), 2.89 (dd, *J* = 16.9, 2.8 Hz, 1H), 2.25 (s, 3H),

1.92 (dd, J = 13.9, 6.2 Hz, 1H), 1.83 (dd, J = 13.9, 6.5 Hz, 1H), 1.63 (hept, J = 6.5 Hz, 1H), 0.91 (d, J = 6.7 Hz, 3H), 0.89 (d, J = 6.6 Hz, 3H).

^{13}C NMR (176 MHz, $CDCl_3$) δ 191.4, 178.7, 161.6, 158.7, 137.3, 133.2, 131.6, 128.9 (2C), 128.4 (2C), 126.5, 125.7, 120.6, 117.8, 80.7, 76.0, 41.1, 38.2, 24.8, 24.0, 23.5, 20.5.

HRMS: calculated for [$C_{23}H_{23}NO_4$+H+]: 378.1700, found: 378.1698.

The er was determined by UPC2 using a chiral Chiralpack IA column gradient from 100% CO_2 up to 40%; i-PrOH, 2.5 mL/min; detection wavelength = 245 nm; τ_{major} = 2.43 min, τ_{minor} = 3.11 min, (80:20 er).

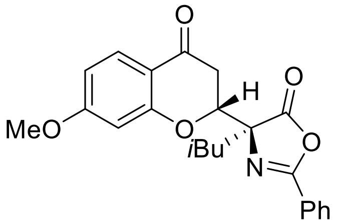

(*S*)-4-Isobutyl-(7-methoxy-(*R*)-4-oxochroman-2-yl)-2-phenyl-1,3-oxazol-5(4*H*)-one (**3o**) pure product was isolated by flash chromatography on silica gel (hexane:ethyl acetate 15:1) as yellow solid (m.p. 160-161 °C) in 65% yield (25.5 mg), dr = 19:1. Major diastereoisomer:

IR (film): 3071, 1819, 1684, 1651,1582, 1486, 1281, 1214, 1099, 883, 700, 561 cm^{-1}.

^{1}H NMR (700 MHz, $CDCl_3$) δ 8.04 (d, J = 7.3 Hz, 2H), 7.59 (t, J = 7.4 Hz, 1H), 7.49 (t, J = 7.8 Hz, 2H), 7.26 (d, J = 3.3 Hz, 1H), 6.99 (dd, J = 9.1, 3.2 Hz, 1H), 6.81 (d, J = 9.1 Hz, 1H), 4.69 (dd, J = 13.1, 2.8 Hz, 1H), 3.75 (s, 3H), 3.19 (dd, J = 16.9, 13.1 Hz, 1H), 2.90 (dd, J = 16.9, 2.9 Hz, 1H), 1.92 (dd, J = 13.9, 6.2 Hz, 1H), 1.83 (dd, J = 13.9, 6.5 Hz, 1H), 1.66 – 1.60 (m, 1H),), 0.91 (d, J = 6.7 Hz, 2H), 0.89 (d, J = 6.6 Hz, 2H).

^{13}C NMR (176 MHz, $CDCl_3$) δ 191.2, 178.6, 161.6, 155.3, 154.6, 133.1, 128.9 (2C), 128.4 (2C), 125.8, 125.3, 120.9, 119.3, 107.4, 80.8, 75.9, 55.9, 41.2, 38.1, 24.8, 24.0, 23.5.

HRMS: calculated for [$C_{23}H_{23}NO_5$+H^+]: 394.1649, found: 394.1645.

The er was determined by UPC2 using a chiral Chiralpack IA column gradient from 100% CO_2 up to 40%; i-PrOH, 2.5 mL/min; detection wavelength = 245 nm; τ_{major} = 2.58 min, τ_{minor} = 3.11 min, (90:10 er).

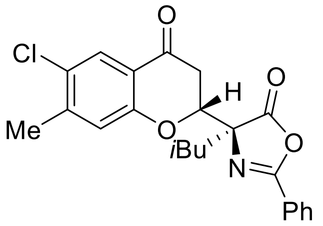

(*S*)-4-Isobutyl-(6-chloro-7-methyl-(*R*)-4-oxochroman-2-yl)-2-phenyl-1,3-oxazol-5(4*H*)-one (**3p**) pure product was isolated by flash chromatography on silica gel (hexane:ethyl acetate 15:1) as yellow oil in 75% yield (30.8 mg), dr = 19:1. Major diastereoisomer:

IR (film): 3065, 1819, 1691, 1652, 1611, 1408, 1319, 1154, 873, 703 cm^{-1}.

^{1}H NMR (700 MHz, $CDCl_3$) δ 8.02 (d, J = 7.6 Hz, 2H), 7.78 (s, 1H), 7.59 (t, J = 7.4 Hz, 1H), 7.49 (t, J = 7.8 Hz, 2H), 6.78 (s, 1H), 4.71 (dd, J = 12.7, 3.0 Hz, 1H), 3.17 (dd, J = 17.0, 12.7 Hz, 1H), 2.90 (dd, J = 17.0, 3.0 Hz, 1H), 2.27 (s, 2H), 1.90 (dd, J = 13.9, 6.2 Hz, 1H), 1.82 (dd, J = 13.9, 6.5 Hz, 1H), 1.63 (hept, J = 6.6 Hz, 1H), 0.91 (d, J = 6.7 Hz, 3H), 0.89 (d, J = 6.6 Hz, 3H).

^{13}C NMR (176 MHz, $CDCl_3$) δ 189.7, 178.5, 161.8, 158.8, 145.4, 133.2, 129.0 (2C), 128.4 (2C), 128.3, 126.6, 125.6, 120.1, 120.0, 81.0, 75.9, 41.1, 37.8, 24.8, 24.0, 23.5, 20.8.

HRMS: calculated for [$C_{23}H_{22}ClNO_4$+H^+]: 412.1310, found: 412.1319.

The er was determined by UPC2 using a chiral Chiralpack IA column gradient from 100% CO_2 up to 40%; i-PrOH, 2.5 mL/min; detection wavelength = 245 nm; τ_{major} = 2.65 min, τ_{mino}r = 2.87 min, (76:24 er).

3. **Synthesis of methyl 2-benzamido-4-methyl-2-(4-oxochroman-2-yl)pentanoate** (**4a**)

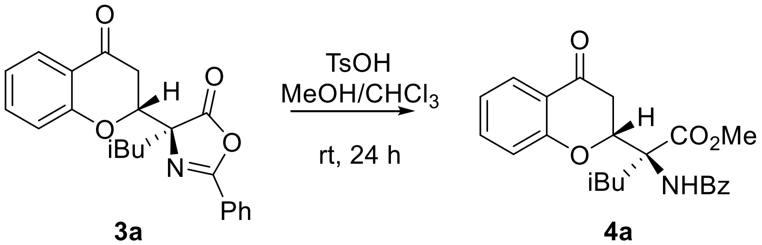

3a
90:10 er
>20:1 dr

4a
51% yield
>20:1 dr

An ordinary screw-cap vial was charged with a magnetic stirring bar, the chromone **3a** (0.05 mmol, 17 mg), MeOH (200 µL) and $CHCl_3$ (100 µL). Then toluenesulphonic acid monohydrate (0.1 mmol, 19 mg) was added and the reaction mixture was stirred for 1.5 h at 40 °C. Product was isolated using flash chromatography in an eluent gradient (starting from hexane:ethyl acetate - 10:1 to hexane:ethyl acetate - 5:1), giving **4a** as a yellow oil in 51% yield (10.0 mg), dr = >20:1 dr. Major diastereoisomer:

IR (film): 3405, 3064, 1819, 1738, 1691, 1669, 1579, 1464, 1442, 1304, 1224, 1030, 765, 710 cm^{-1}.

^{1}H NMR (700 MHz, $CDCl_3$) δ 7.85 (d, *J* = 7.9 Hz, 1H), 7.82 (d, *J* = 7.9 Hz, 2H), 7.53 (t, *J* = 7.5 Hz, 1H), 7.49 (bs, 1H), 7.46 (t, *J* = 7.8 Hz, 3H), 7.01 (t, *J* = 7.5 Hz, 1H), 6.95 (d, *J* = 7.9 Hz, 1H), 5.03 (dd, *J* = 14.0, 2.4 Hz, 1H), 3.87 (s, 3H), 3.05 (dd, *J* = 14.1, 5.0 Hz, 1H), 3.02 (dd, *J* = 16.9, 2.5 Hz, 1H), 2.85 (dd, *J* = 16.9, 14.1 Hz, 1H), 1.95 (dd, *J* = 14.6, 7.3 Hz, 1H), 1.71 – 1.63 (m, 1H), 0.96 (d, *J* = 7.3 Hz, 3H), 0.86 (d, *J* = 7.2 Hz, 3H).

^{13}C NMR (176 MHz, $CDCl_3$) δ 191.5, 173.2, 166.8, 161.0, 135.9, 134.8, 131.8, 128.7 (2C), 127.0 (2C), 127.0, 121.8, 121.0, 117.7, 81.4, 67.2, 53.3, 39.3, 37.6, 24.7, 23.7, 22.3.

HRMS: calculated for $[C_{23}H_{25}NO_5+H^+]$: 396.1805, found: 396.1812.

4. X-ray crystallography

The single crystal was performed in room temperature in mixture of solvents (dichloromethane:hexane 1mL : 4mL). The single crystal X-ray diffraction study at 100 K revealed that compound **3h** ($C_{22}H_{20}ClNO_4$) crystallizes in the non-centrosymmetric orthorhombic space group $P2_12_12_1$ (Z = 4) and the crystal structure consists of one crystallographically independent formula unit in the unit cell.

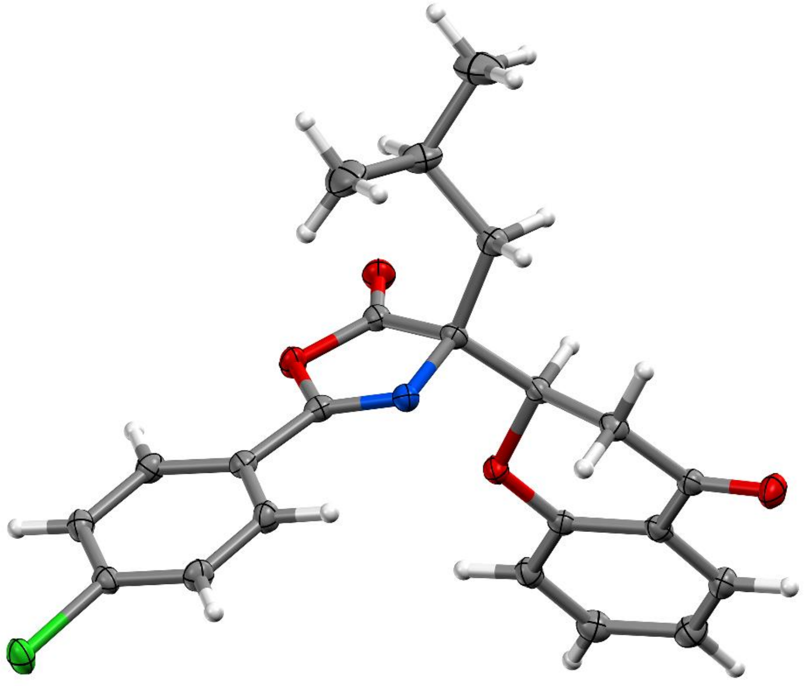

The molecular structure of the compound **3h**, showing 50% probability displacement ellipsoids. Hydrogen atoms are drawn with an arbitrary radius

Single crystal X-ray diffraction data were collected at 100 K by the ω-scan technique using a RIGAKU XtaLAB Synergy, Dualflex, Pilatus 300K diffractometer [3] with PhotonJet micro-focus X-ray Source Cu-Kα (λ = 1.54184 Å). The crystal structure was solved by using direct methods with the SHELXT 2018/2 program [4]. Atomic scattering factors were taken from the International Tables for X-ray Crystallography. Positional parameters of non-H-atoms were refined by a full-matrix least-squares method on F^2 with anisotropic thermal parameters by using the SHELXL 2018/3 program [5]. All hydrogen atoms were placed in calculated positions (C–H = 0.95–1.00 Å) and included as riding contributions with isotropic displacement parameters set to 1.2–1.5 times the U_{eq} of the parent atom.

3h: Formula $C_{22}H_{20}ClNO_4$, orthorhombic, space group $P2_12_12_1$, Z = 4, unit cell constants a = 8.51518(4), b = 11.67771(5), c = 19.88318(9) Å, V = 1977.140(15) Å^3. The integration of the data yielded a total of 24723 reflections with θ angles in the range of 4.39 to 66.60°, of which all 3484 unique (R_{int} = 1.64%) were greater than 2σ(F^2). The final anisotropic full-matrix least-squares refinement on F^2 with 256 parameters

converged at R_1 = 1.98% and wR_2 = 4.99% for all data. The largest peak in the final difference electron density synthesis was 0.174 e Å^{-3} and the largest hole was -0.134 e Å^{-3}. The goodness-of-fit was 1.112. The absolute configuration was unambiguously determined from anomalous scattering, by calculating the x Flack parameter [6] of 0.008(2) using 1466 quotients.

CCDC 1895323 contains the supplementary crystallographic data for this paper. These data can be obtained free of charge from The Cambridge Crystallographic Data Centre via https://www.ccdc.cam.ac.uk/structures/

In order to confirm that the crystal structure of the major enantiomer of **3h** was established, obtained crystal was redissolved in CH_2Cl_2 and subjected to UPC^2 analysis using a chiral Chiralpack IG column gradient from 100% CO_2 up to 40%; i-PrOH, 2.5 mL/min; detection wavelength = 245 nm (previously established separation conditions for **3h** - τ_{major} = 2.57 min, τ_{minor} = 3.40 m). Only a peak corresponding to the major enantiomer was observed:

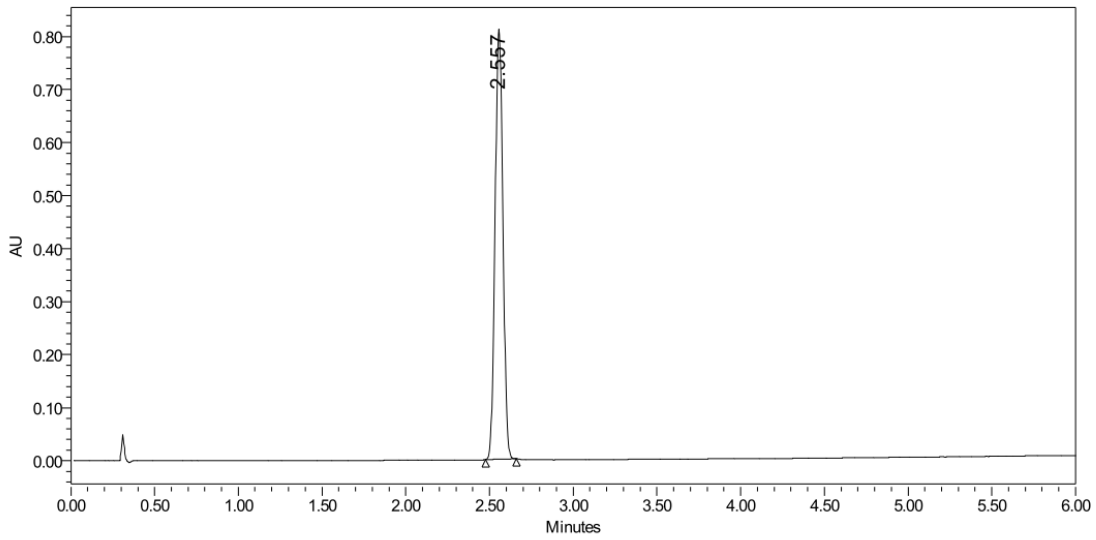

5. References

[1] Liang, J. ; Ruble, J. C.; Fu, G. C. *J. Org. Chem.* **1998**, *63*, 3154.

[2] Ishizuka, N.; Matsunori, K.; Sakai, K.; Fujimoto, M.; Mihara, S.; Yamamori, T. *J. Med. Chem.* **2002**, *45*, 2041

[3] Rigaku OD. CrysAlis PRO. Rigaku Oxford Diffraction Ltd, Yarnton, Oxfordshire, England, **2015**.

[4] Sheldrick, G. M. "SHELXT - integrated space-group and crystal-structure determination", *Acta Cryst.* **2015**, *A71*, 3-8.

[5] Sheldrick, G. M. "Crystal structure refinement with SHELXL", *Acta Cryst.* **2015**, *C71*, 3-8.

[6] Parsons, S.; Flack, H. D.; Wagner, T. "Use of intensity quotients and differences in absolute structure refinement" *Acta Cryst.* **2013**, *B69*, 249-259.

6. NMR Data

(*S*)-4-Isobutyl-((*R*)-4-oxochroman-2-yl)-2-phenyl-1,3-oxazol-5(4*H*)-one (3a)

¹H NMR

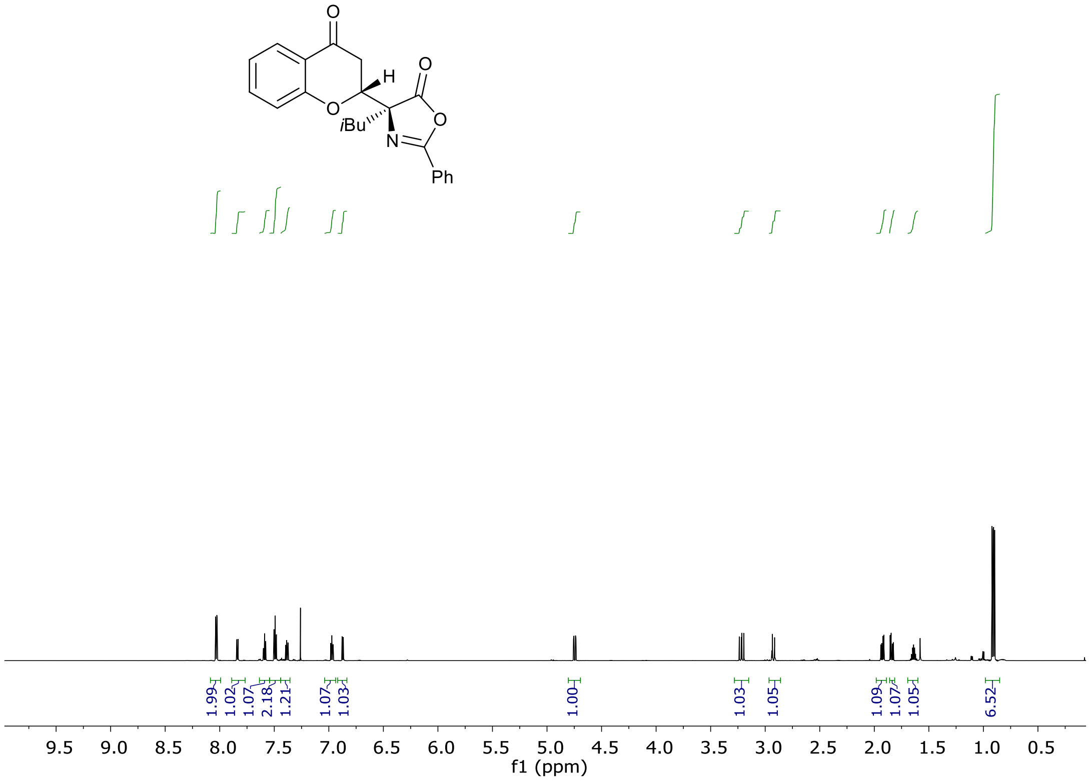

¹³C NMR

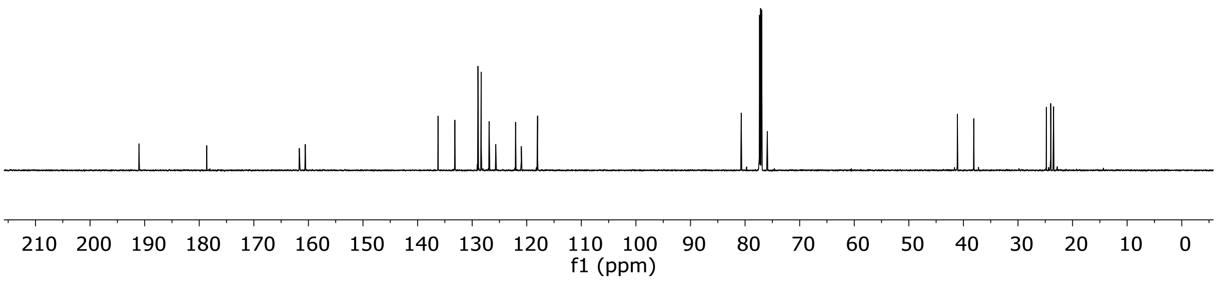

((*R*)-4-Oxochroman-2-yl)-2-phenyl-(*S*)-4-isopropan-2-yl-1,3-oxazol-5(4*H*)-one (3b)

^{1}H NMR

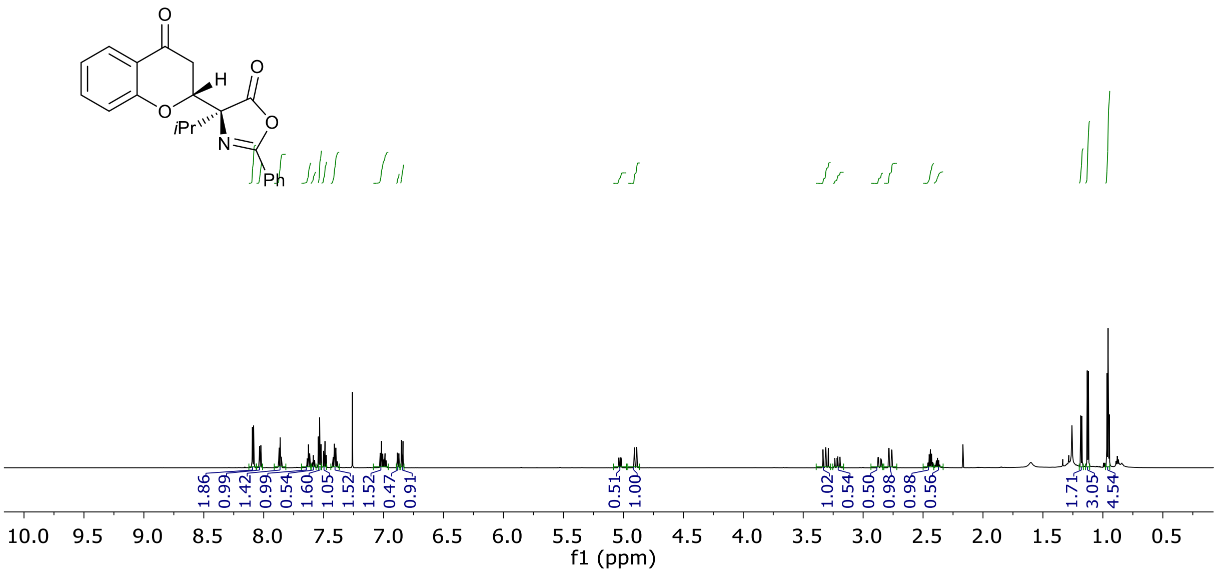

^{13}C NMR

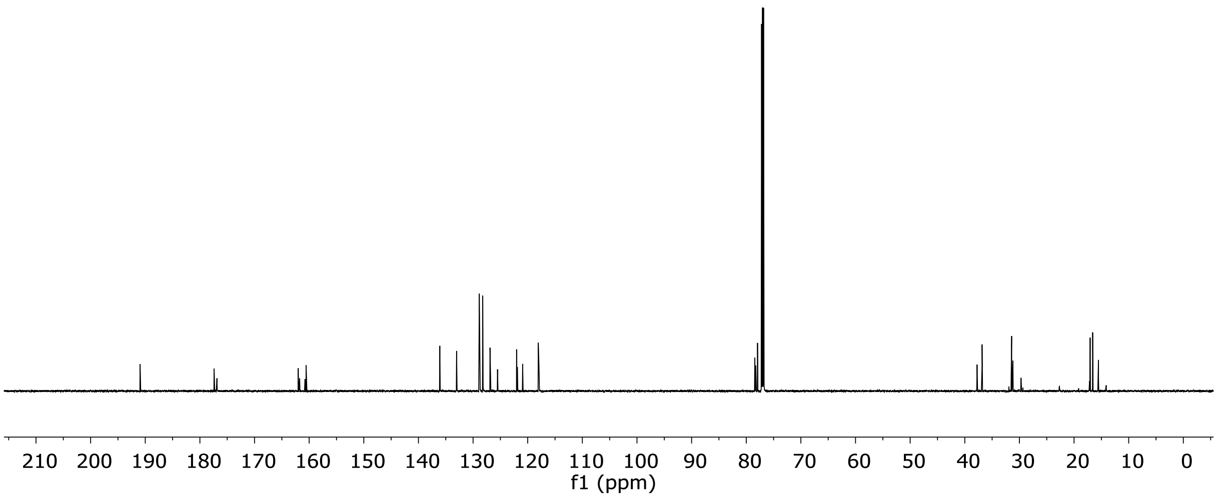

(*S*)-4-Ethyl-(*R*)-4-oxochroman-2-yl-2-phenyl-1,3-oxazol-5(4*H*)-one (3c)

^{1}H NMR

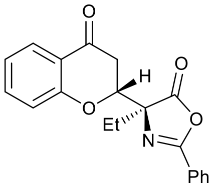

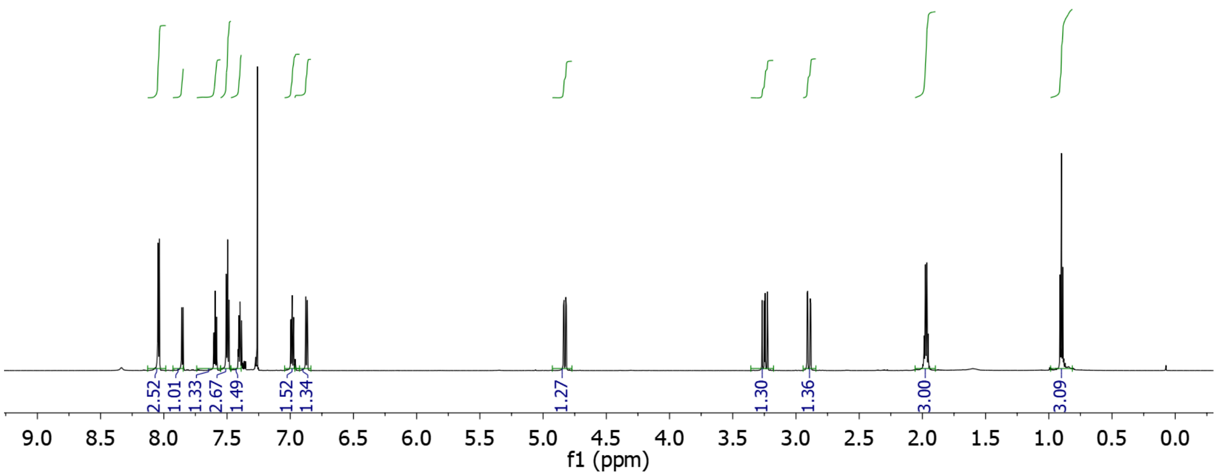

^{13}C NMR

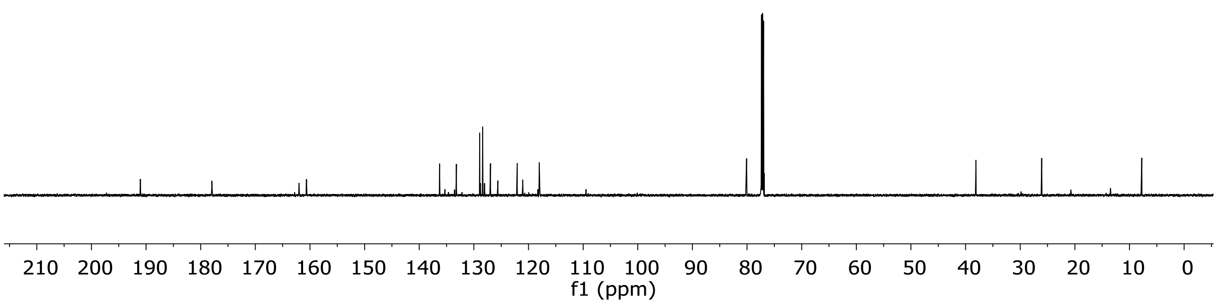

(*S*)-4-Methyl-2-((*R*)-4-oxochroman-2-yl)-2-phenyl-1,3-oxazol-5(4*H*)-one (3d)

^{1}H NMR

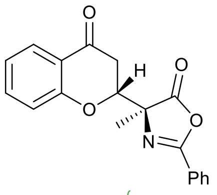

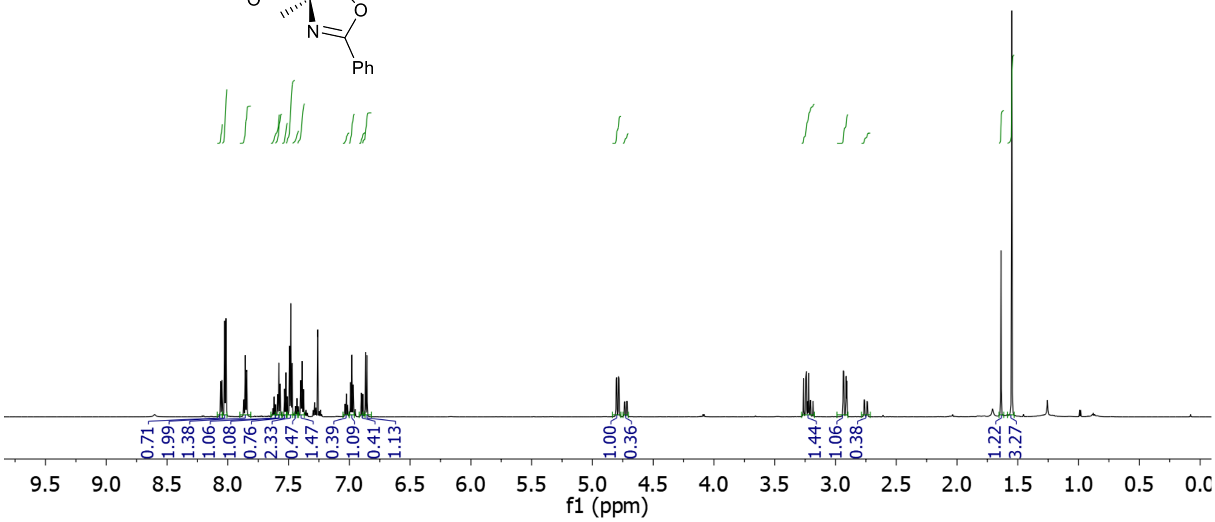

^{13}C NMR

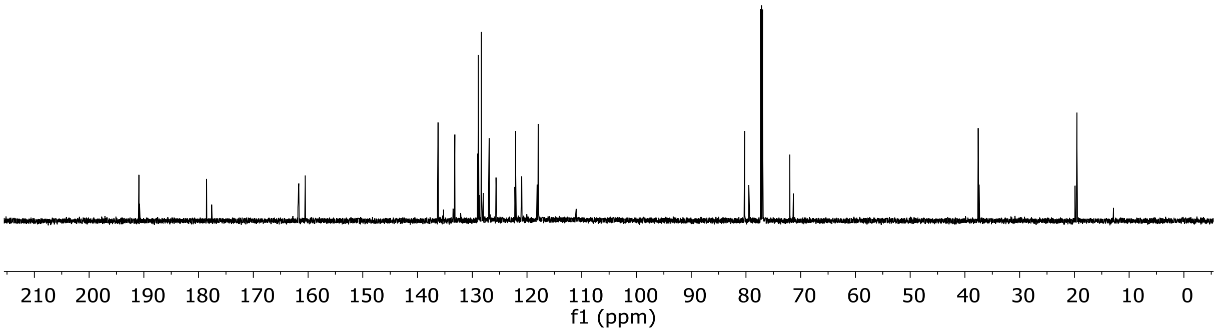

(*S*)-4-Benzyl-(*R*)-4-oxochroman-2-yl-2-phenyl-1,3-oxazol-5(4*H*)-one (3e)

^{1}H NMR

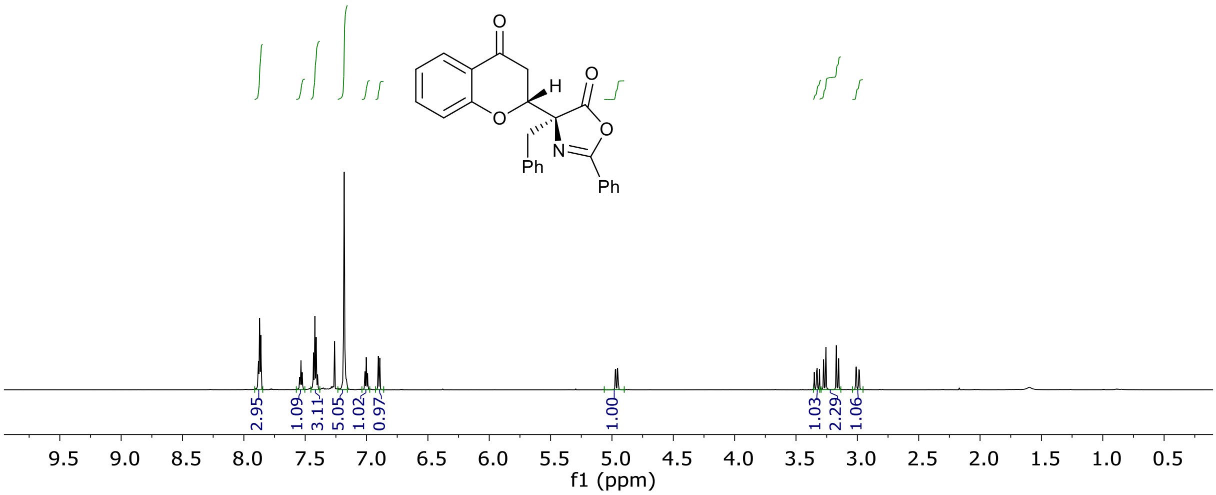

^{13}C NMR

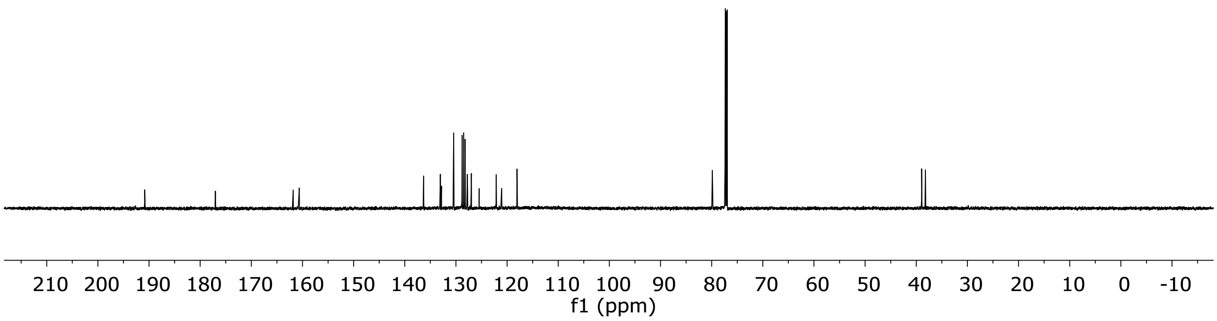

(*S*)-4-(2-(Methylthio)ethyl)-2-((*R*)-4-oxochroman-2-yl)-2-phenyl-1,3-oxazol-5(4*H*)-one (3f)

^{1}H NMR

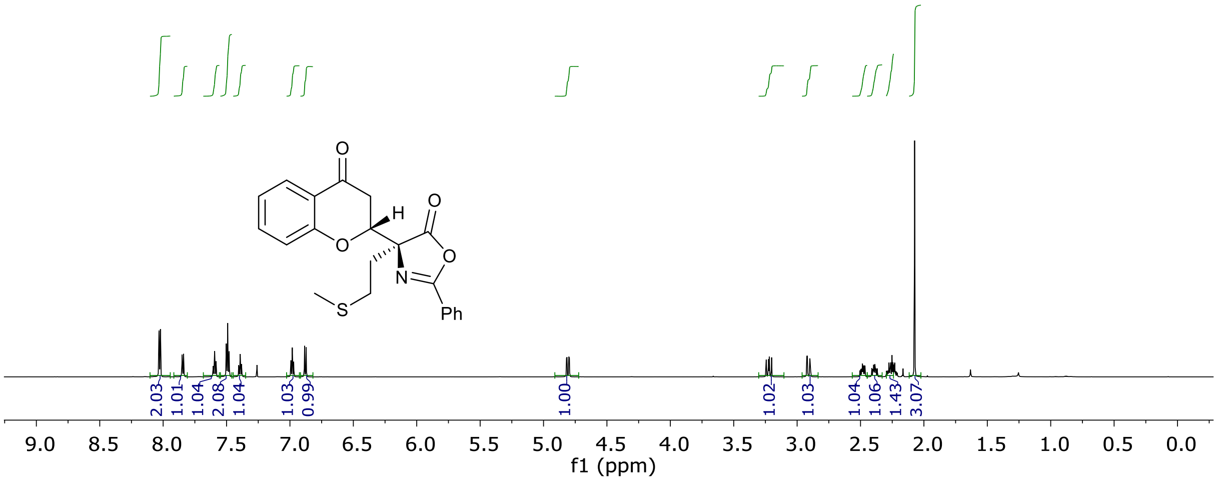

^{13}C NMR

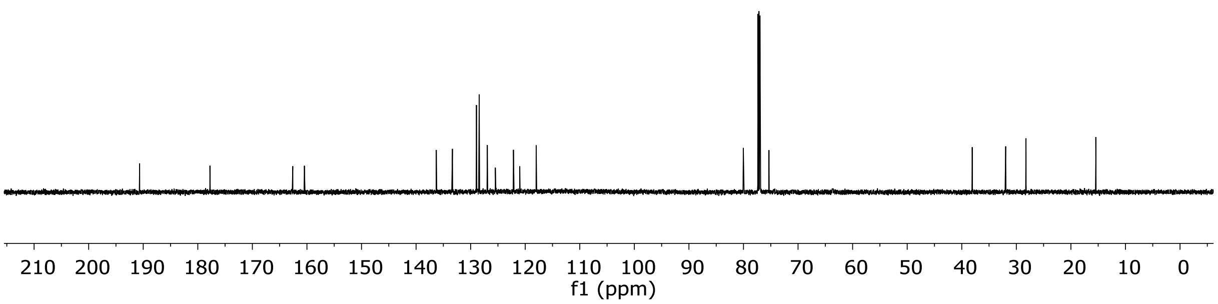

(*S*)-4-Isobutyl-(2-chlorophenyl)-((*R*)-4-oxochroman-2-yl)-2-1,3-oxazol-5(4*H*)-one (3g)

¹H NMR

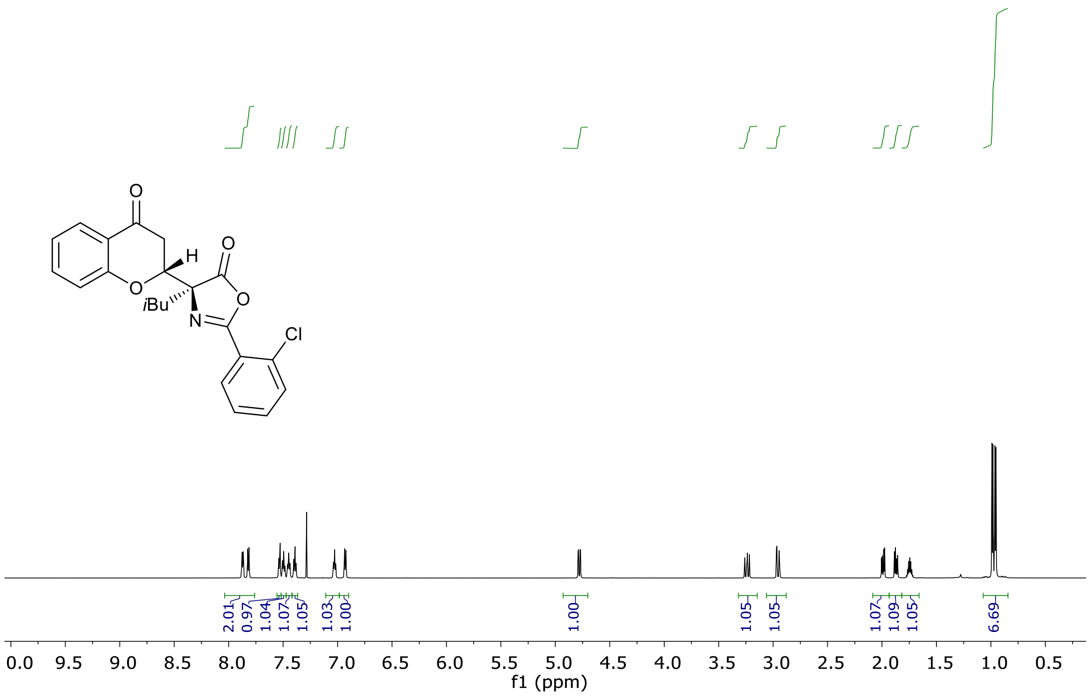

¹³ CNMR

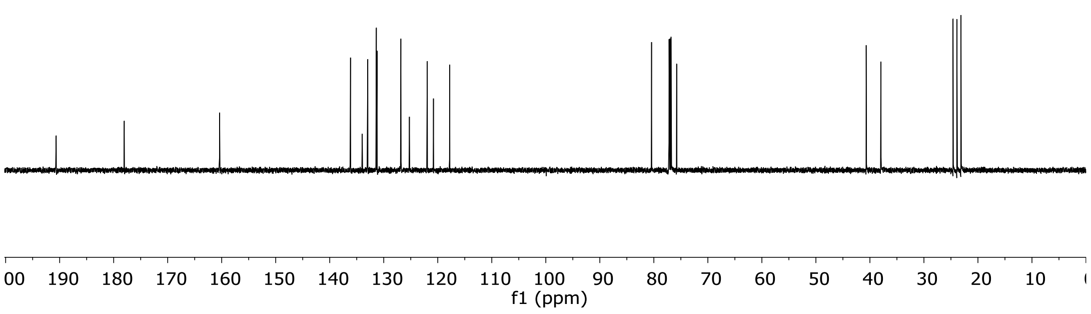

(*S*)-4-Isobutyl-(4-chlorophenyl)-((*R*)-4-oxochroman-2-yl)-2-1,3-oxazol-5(4*H*)-one (3h)

^{1}H NMR

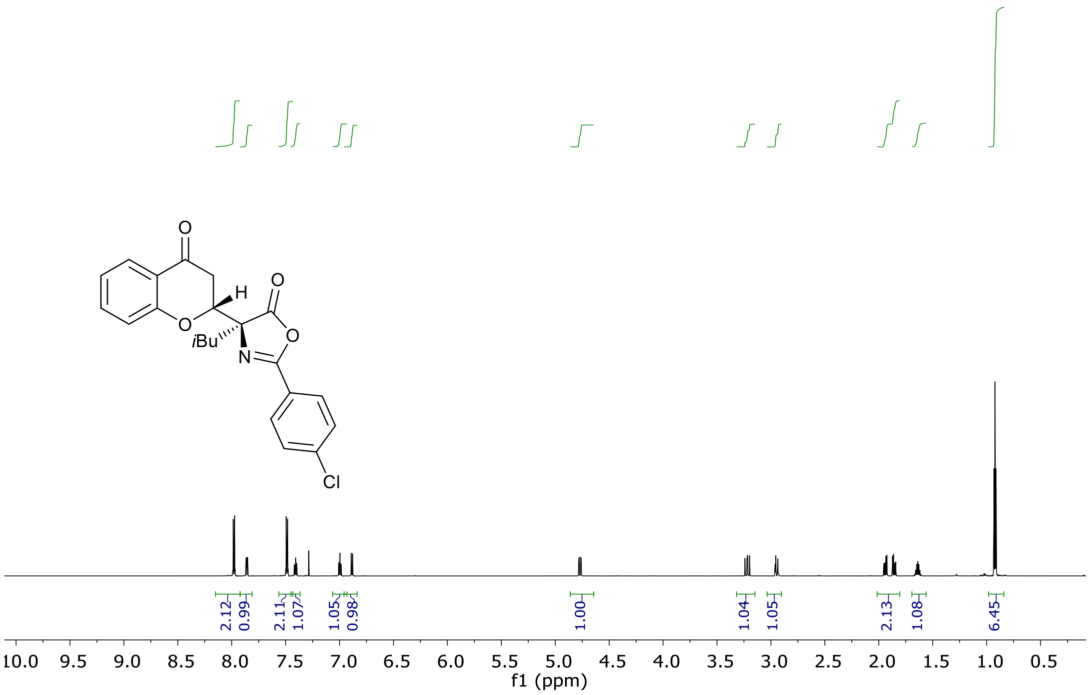

^{13}C NMR

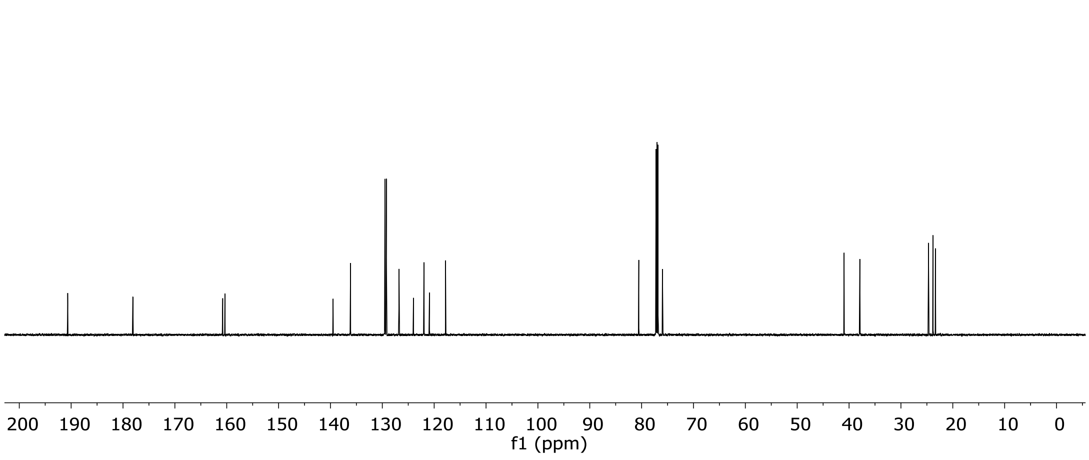

(*S*)-4-Isobutyl-2-(4-nitrophenyl)((*R*)-4-oxochroman-2-yl)-1,3-oxazol-5(4*H*)-one (3i)

^{1}H NMR

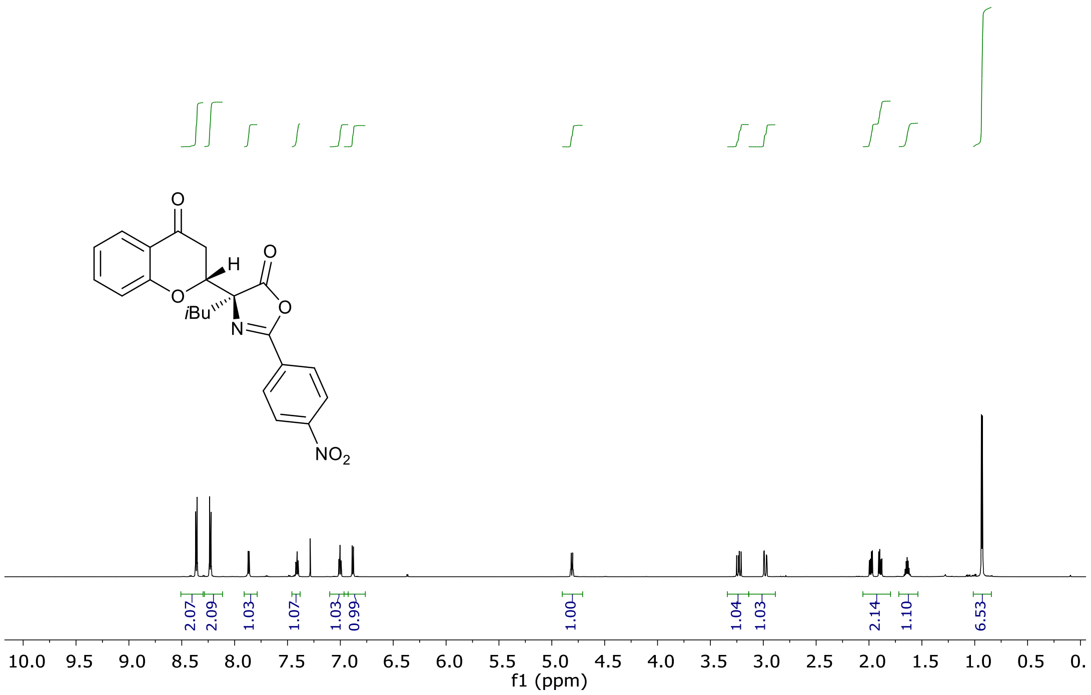

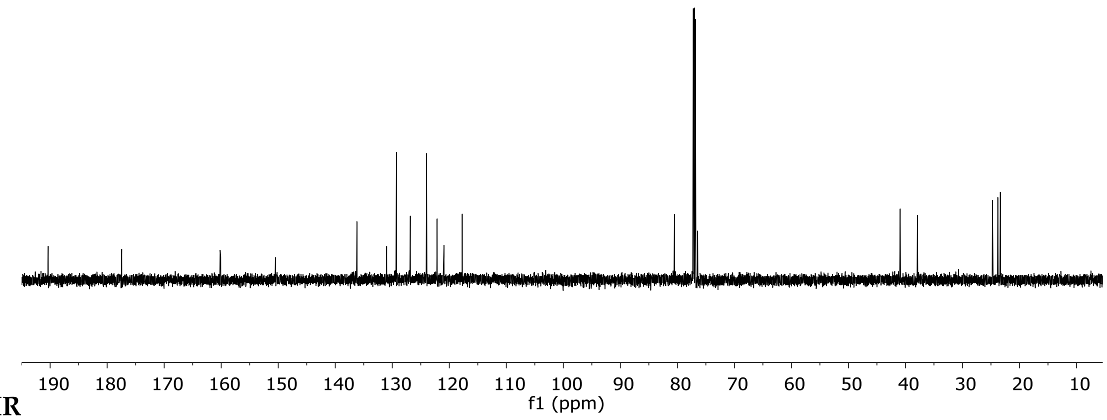

^{13}C NMR

(*S*)-4-Isobutyl-(6-fluoro-(*R*)-4-oxochroman-2-yl)-2-phenyl-1,3-oxazol-5(4*H*)-one (3j)

^{1}H NMR

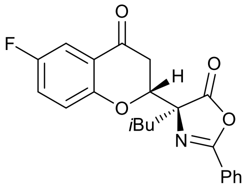

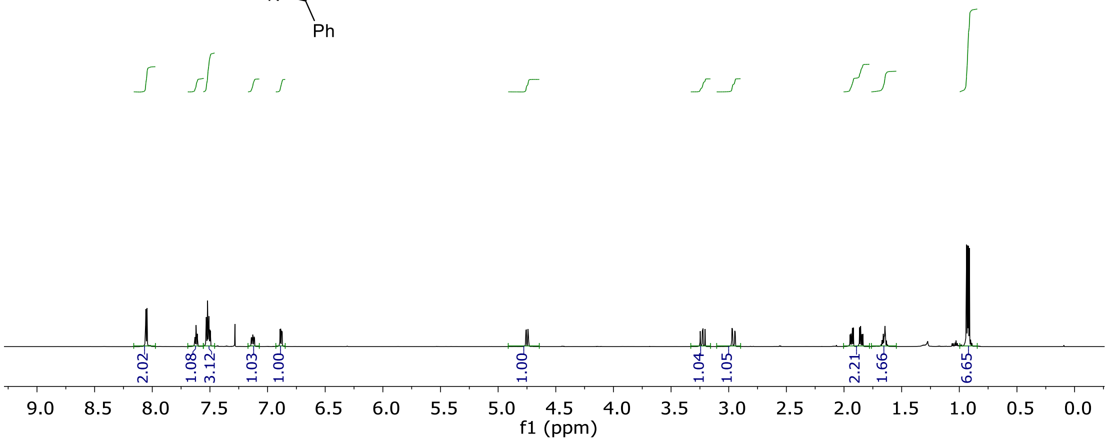

^{13}C NMR

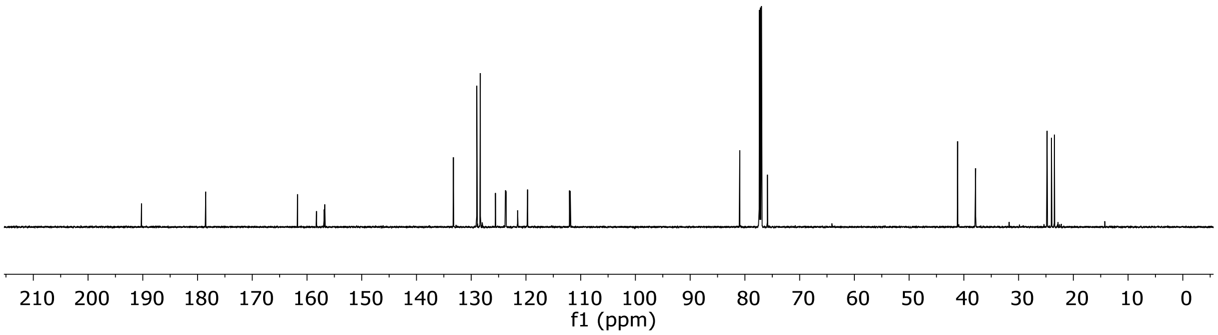

(6-Bromo-(*R*)-4-oxochroman-2-yl)-(*S*)-4-isobutyl-2-phenyl-1,3-oxazol-5(4*H*)-one (3k)

^{1}H NMR

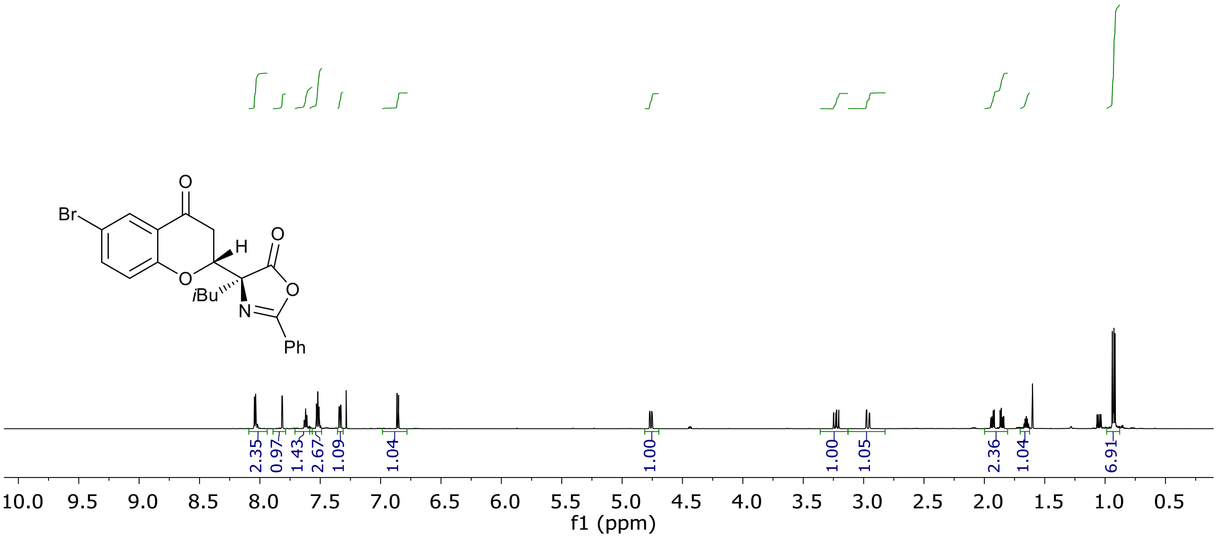

^{13}C NMR

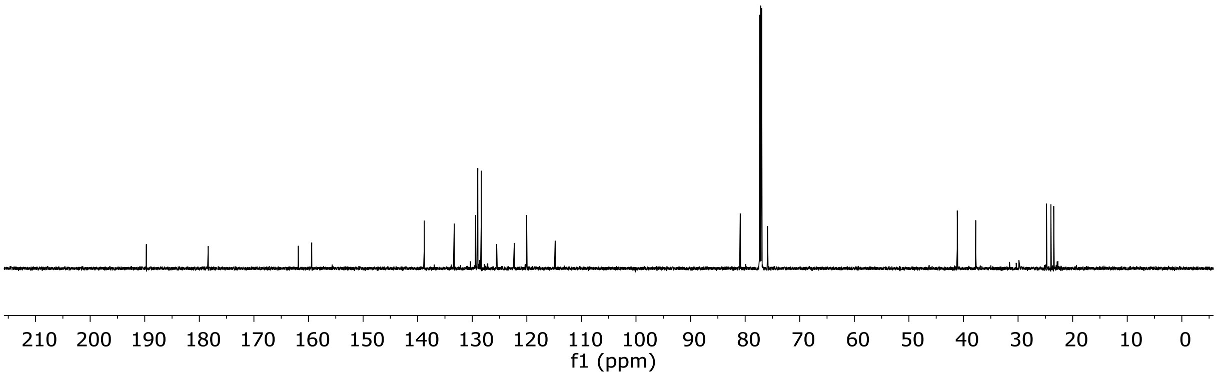

(6-Chloro-(*R*)-4-oxochroman-2-yl)-(*S*)-4-isobutyl-2-phenyl-1,3-oxazol-5(4*H*)-one (3l)

^{1}H NMR

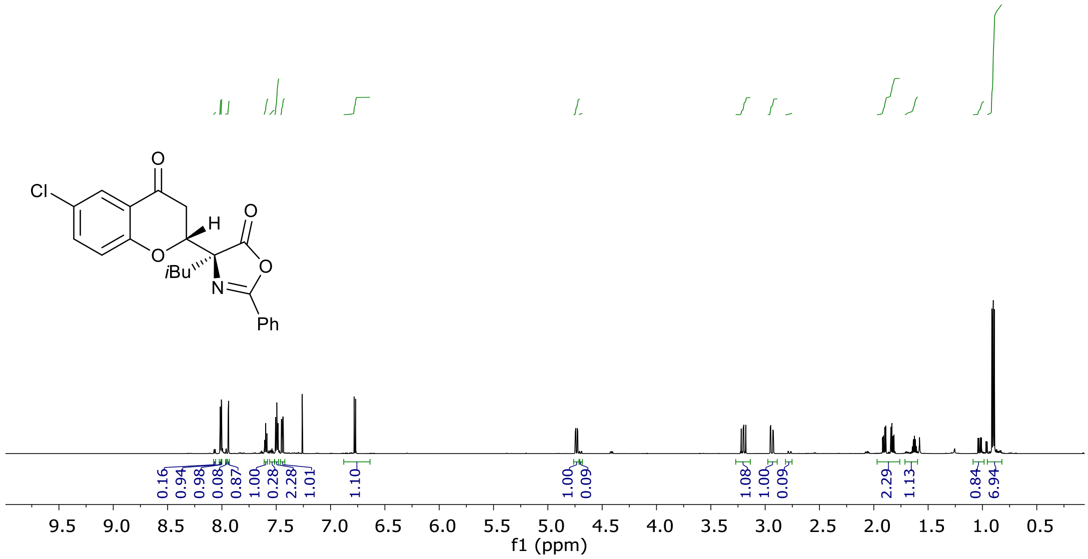

^{13}C NMR

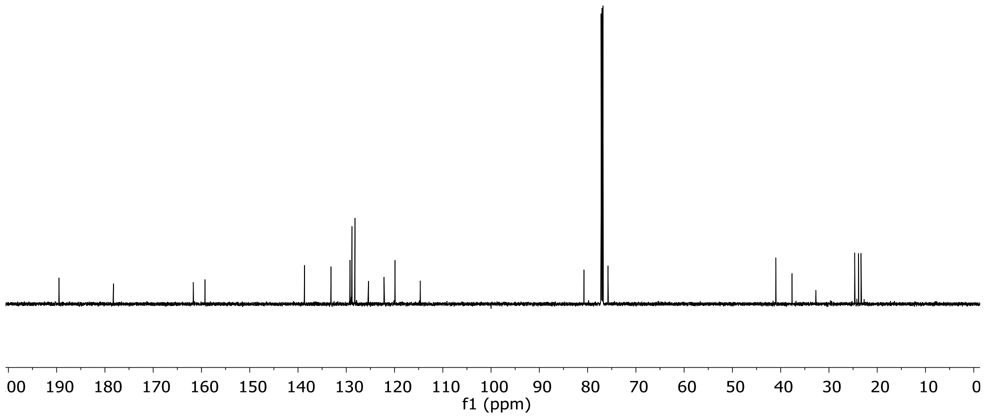

(*S*)-4-Isobutyl-(6-nitro-(*R*)-4-oxochroman-2-yl)-2-phenyl-1,3-oxazol-5(4*H*)-one (3m)

^{1}H NMR

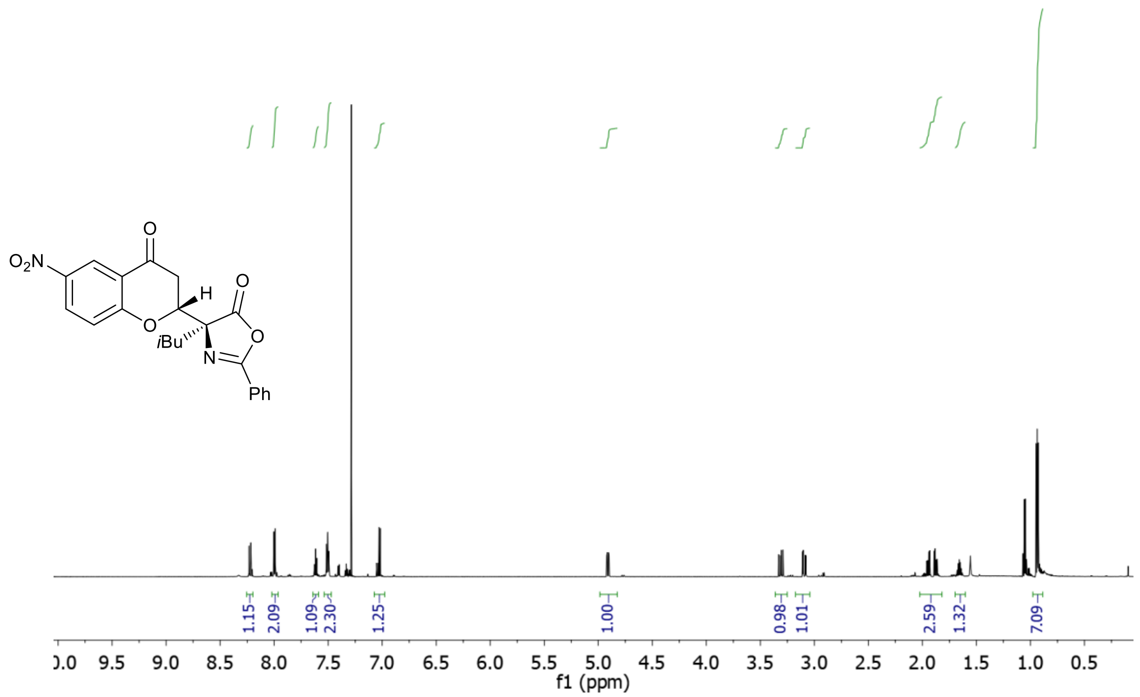

^{13}C NMR

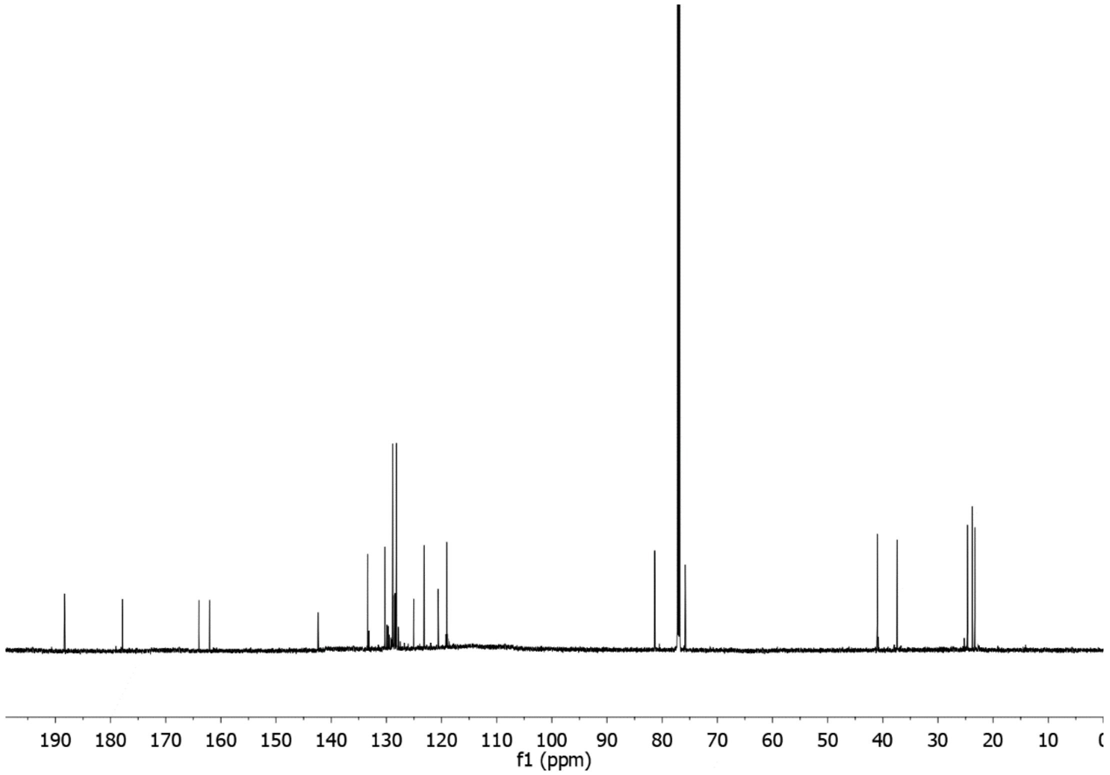

(*S*)-4-Isobutyl-(6-methyl-(*R*)-4-oxochroman-2-yl)-2-phenyl-1,3-oxazol-5(4*H*)-one (3n)

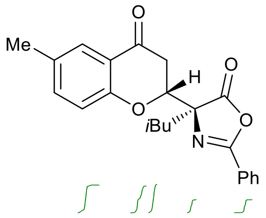

^{1}H NMR

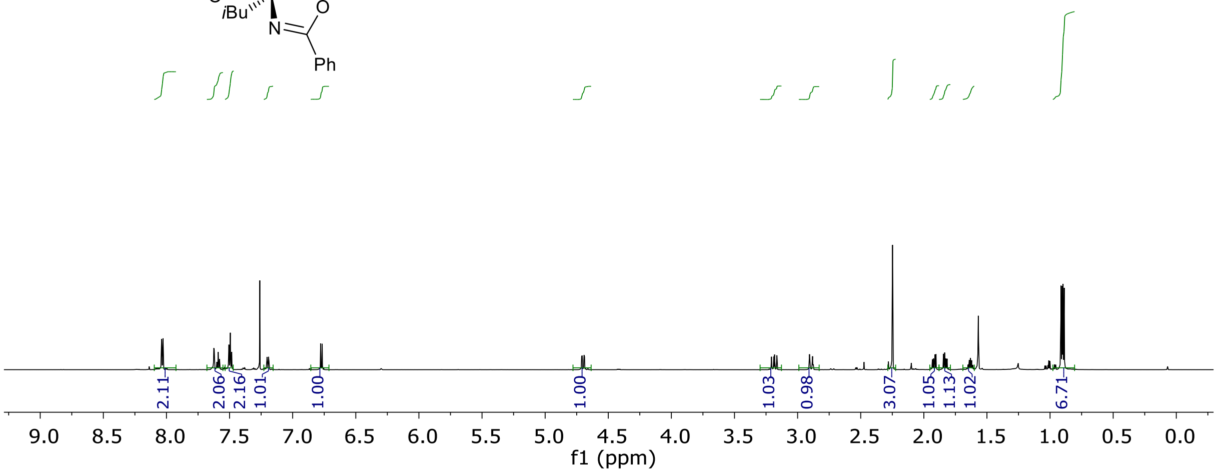

^{13}C NMR

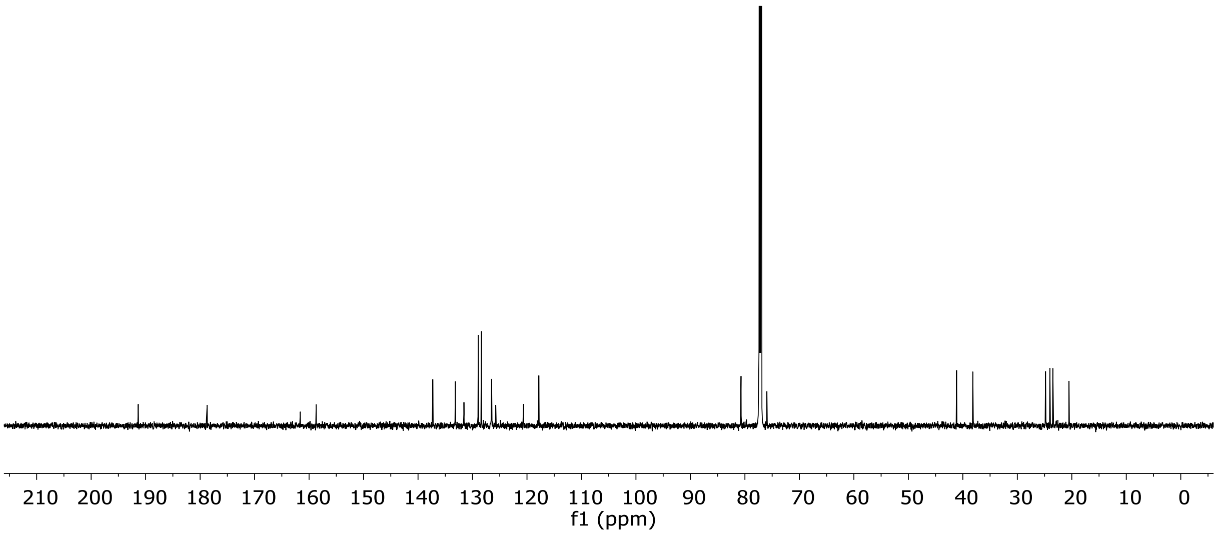

(*S*)-4-Isobutyl-(7-methoxy-(*R*)-4-oxochroman-2-yl)-2-phenyl-1,3-oxazol-5(4*H*)-one (3o)

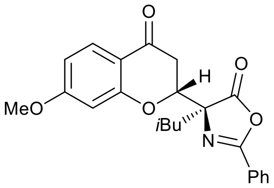

^{1}H NMR

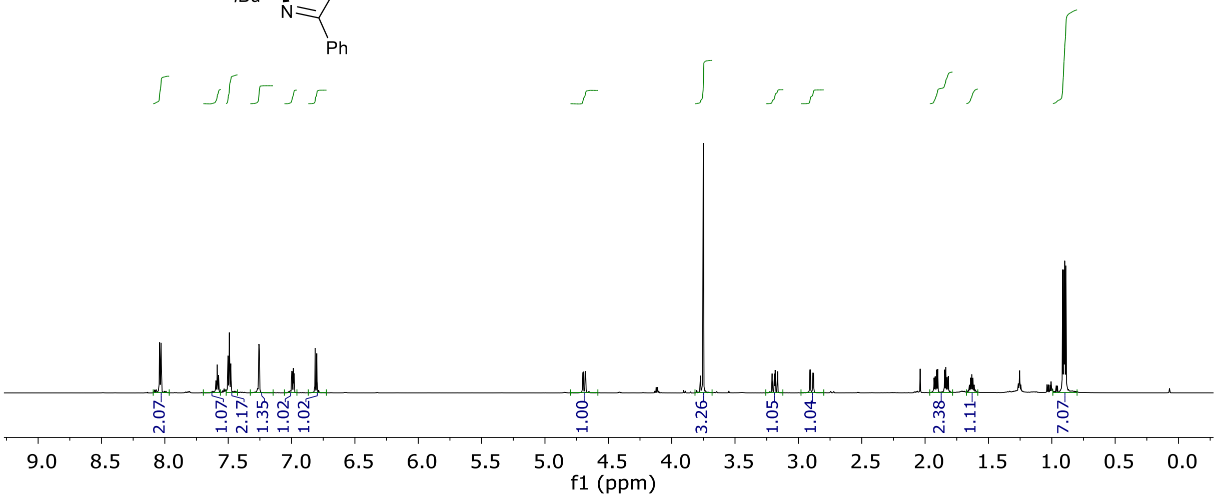

^{13}C NMR

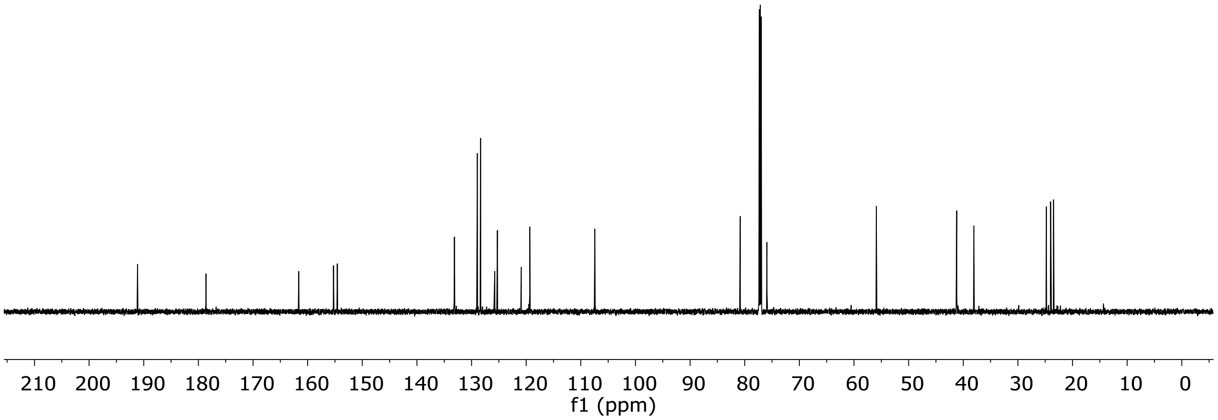

(*S*)-4-Isobutyl-(6-chloro-7-methyl-(*R*)-4-oxochroman-2-yl)-2-phenyl-1,3-oxazol-5(4*H*)-one (3p)

^{1}H NMR

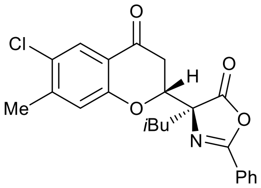

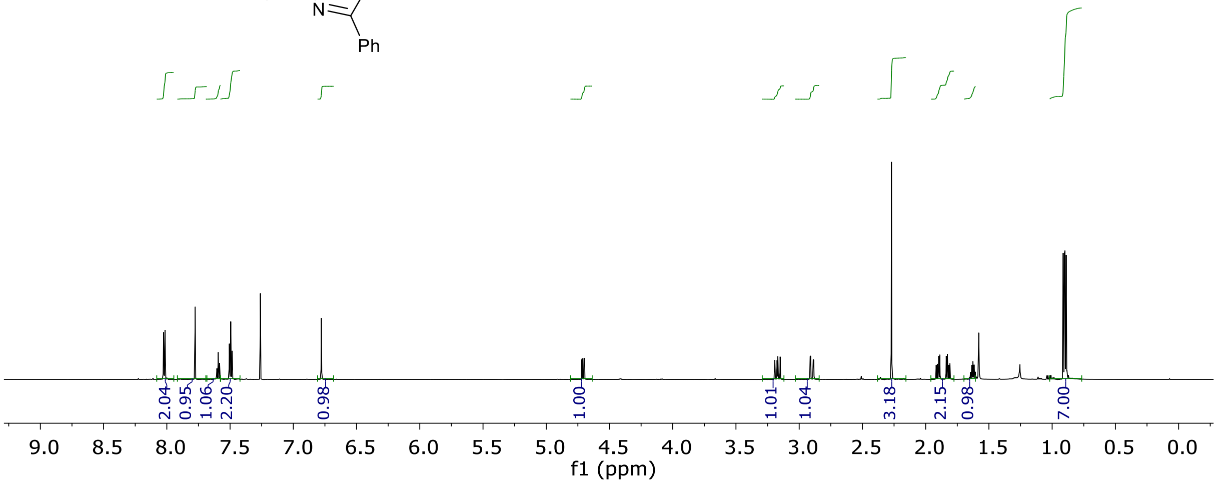

^{13}C NMR

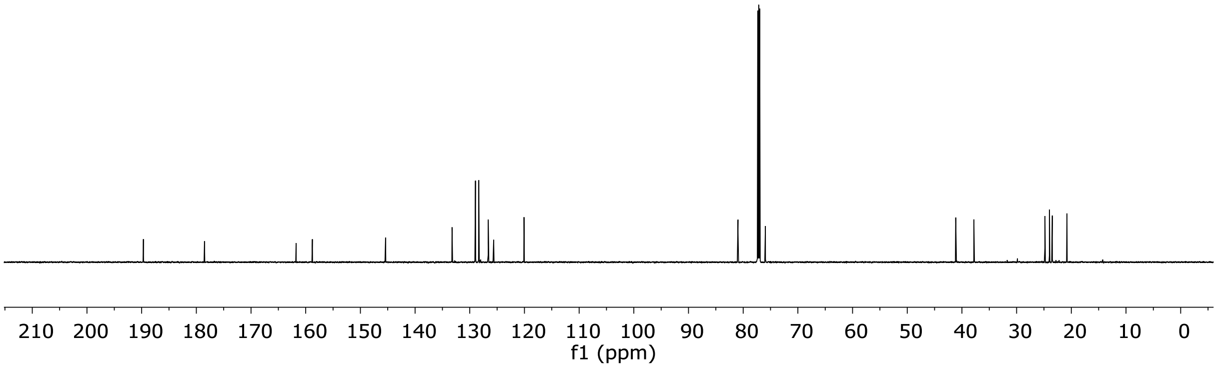

Methyl (*S*)-2-benzamido-4-methyl-2-((*R*)-4-oxochroman-2-yl)pentanoate (4a)

^{1}H NMR

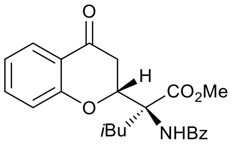

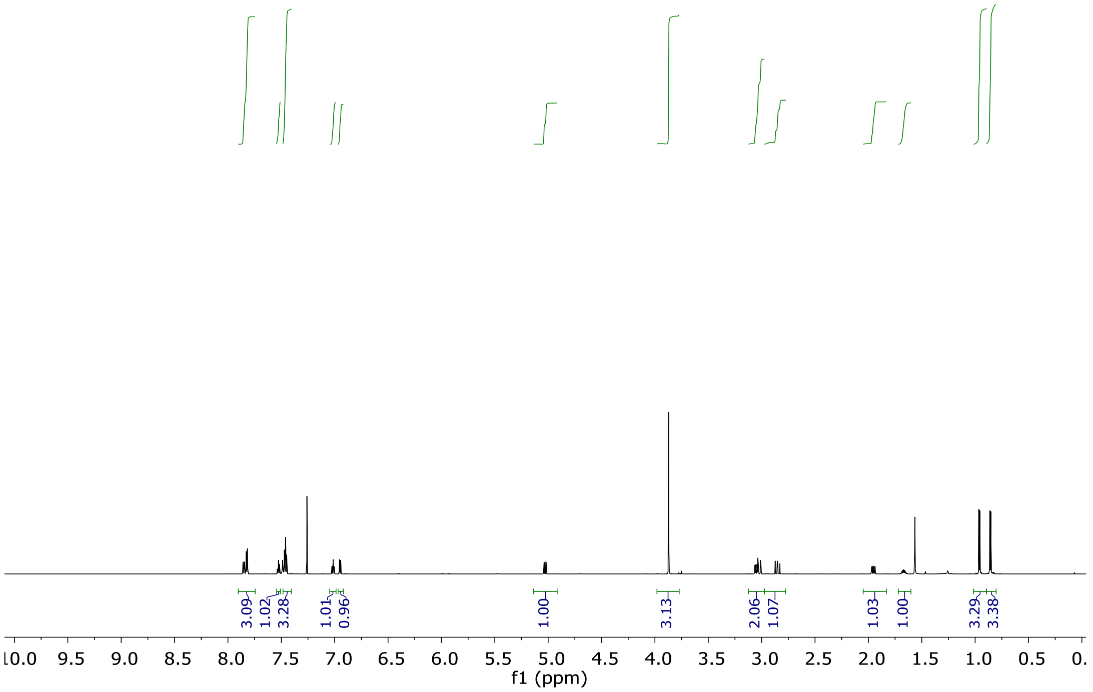

^{13}C NMR

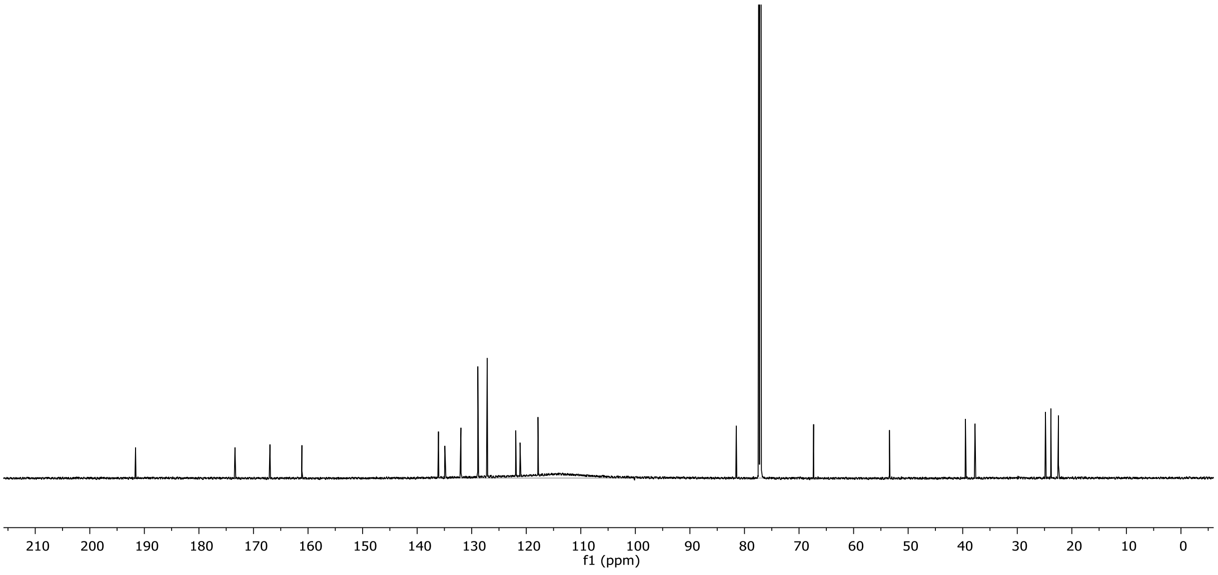

7. HPLC traces

(*S*)-4-Isobutyl-((*R*)-4-oxochroman-2-yl)-2-phenyl-1,3-oxazol-5(4*H*)-one (3a)

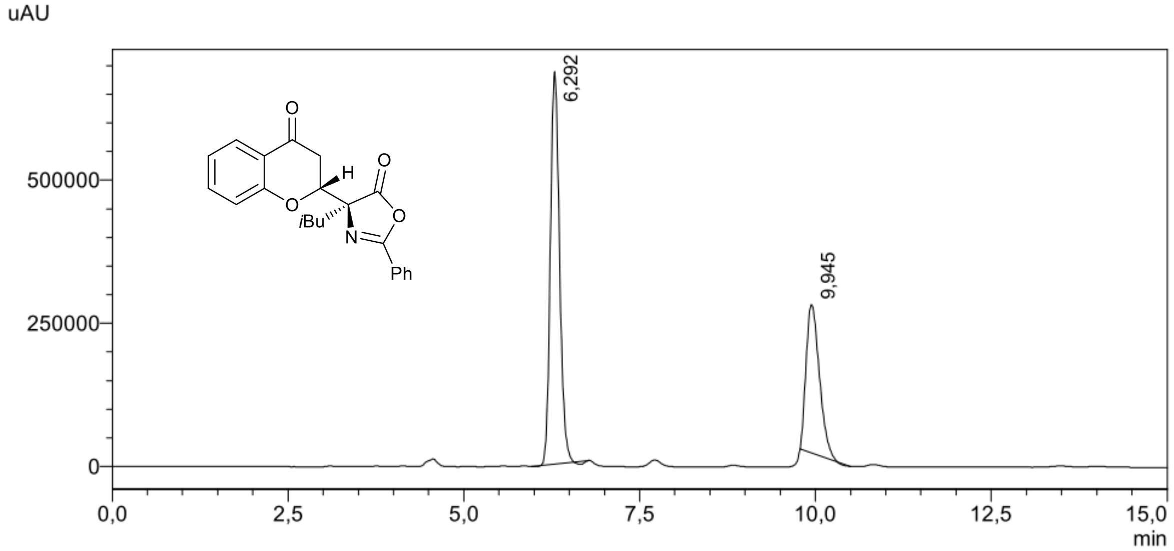

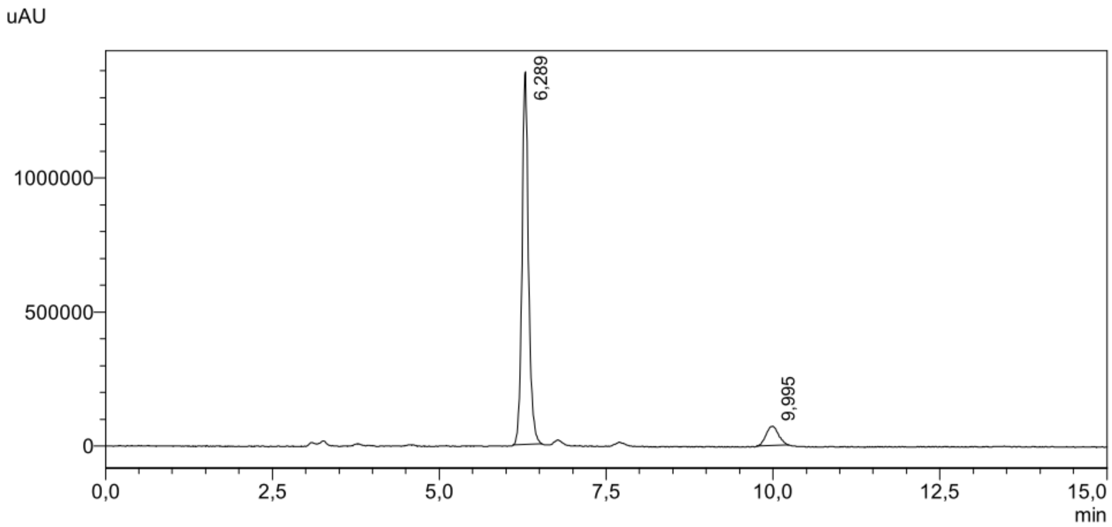

Peak#	Ret. Time	Area%
1	6,289	91,041
2	9,995	8,959
Total		100,000

((*R*)-4-Oxochroman-2-yl)-2-phenyl-(*S*)-4-isopropan-2-yl-1,3-oxazol-5(4*H*)-one (3b)

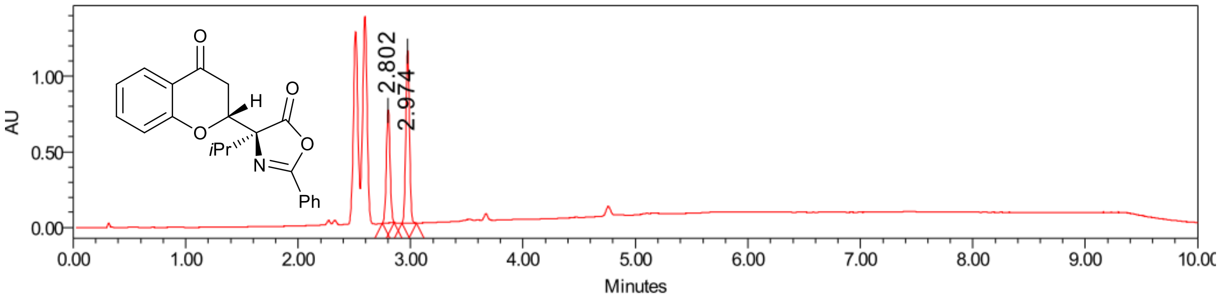

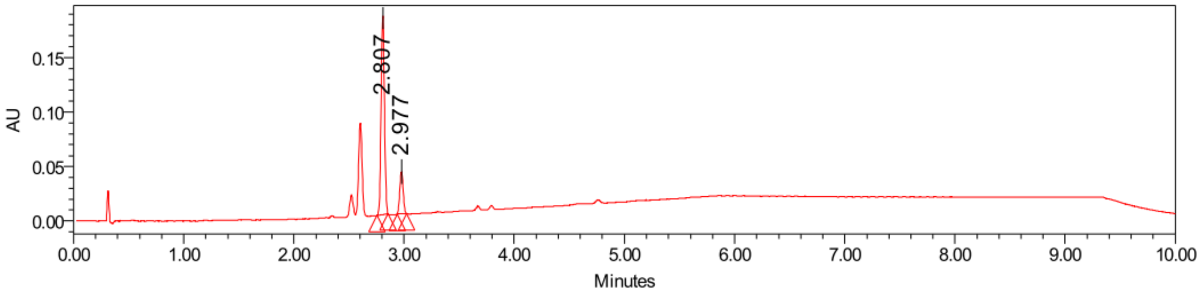

	RT	Area	% Area	Height
1	2.807	389445	82.52	182443
2	2.977	82473	17.48	38742

(*R*)-4-Oxochroman-2-yl-(*S*)-4-ethyl-2-phenyl-1,3-oxazol-5(4*H*)-one (3c)

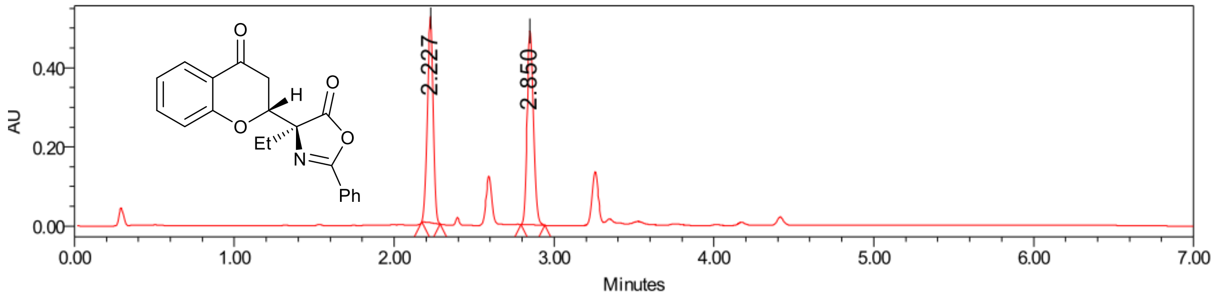

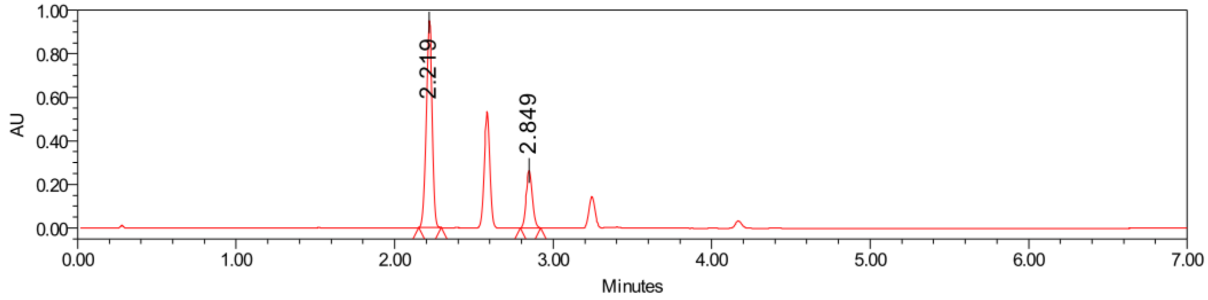

	RT	Area	% Area	Height
1	2.219	2498384	77.51	952057
2	2.849	724739	22.49	265046

(*S*)-4-Methyl-2-((*R*)-4-oxochroman-2-yl)-2-phenyl-1,3-oxazol-5(4*H*)-one (3d)

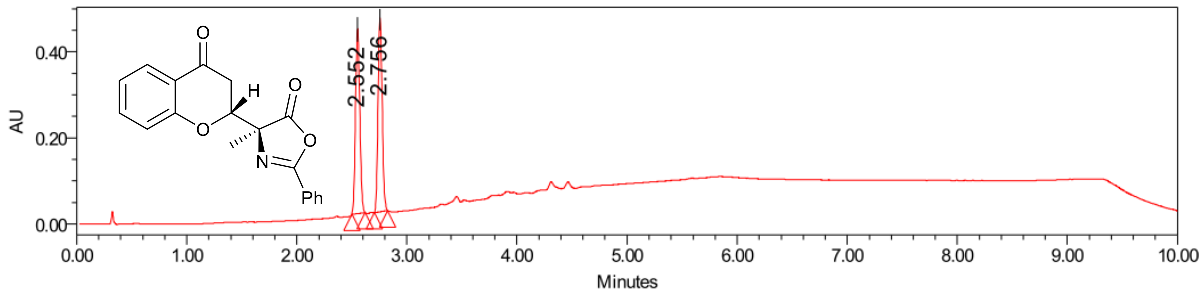

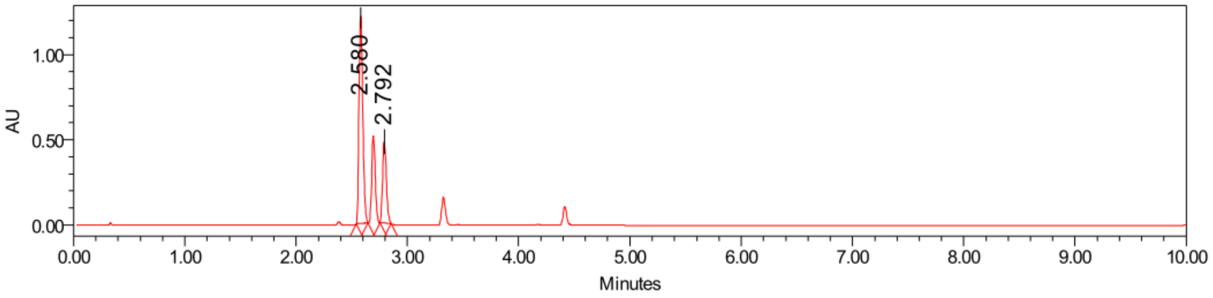

	RT	Area	% Area	Height
1	2.580	2860137	71.56	1216921
2	2.792	1136912	28.44	478610

(*S*)-4-Benzyl-(*R*)-4-oxochroman-2-yl-2-phenyl-1,3-oxazol-5(4*H*)-one (3e)

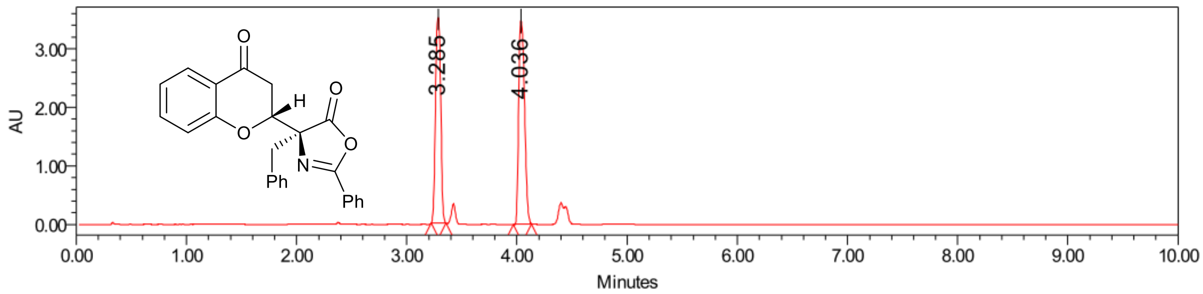

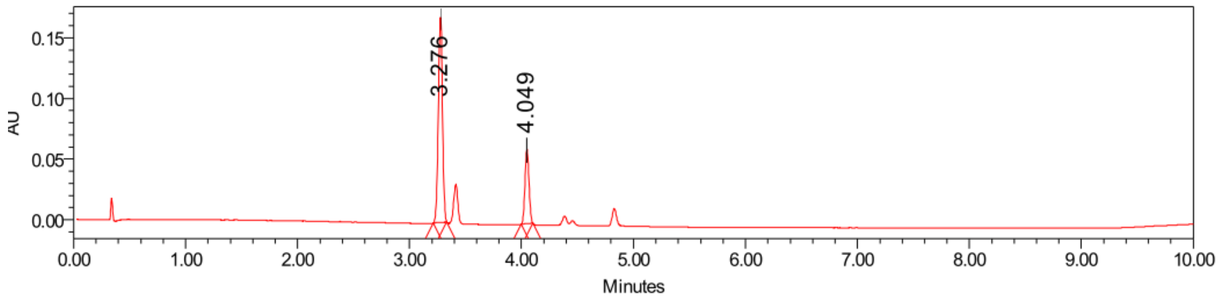

	RT	Area	% Area	Height
1	3.276	432507	73.94	169127
2	4.049	152458	26.06	60768

(*S*)-4-(2-(Methylthio)ethyl)-2-((*R*)-4-oxochroman-2-yl)-2-phenyl-1,3-oxazol-5(4*H*)-one (3f)

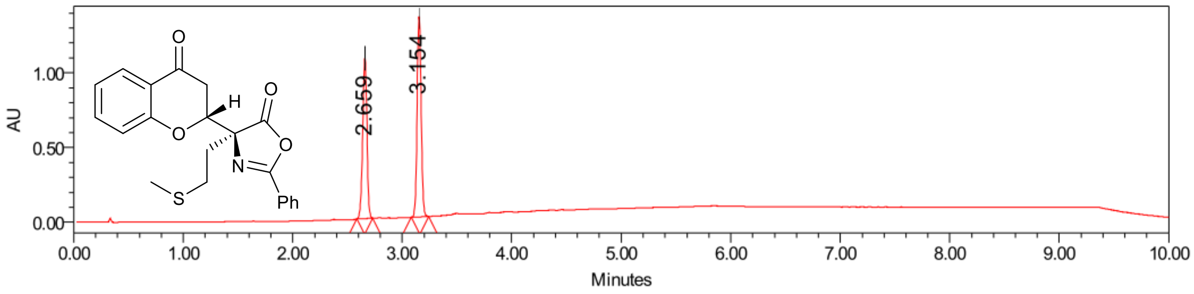

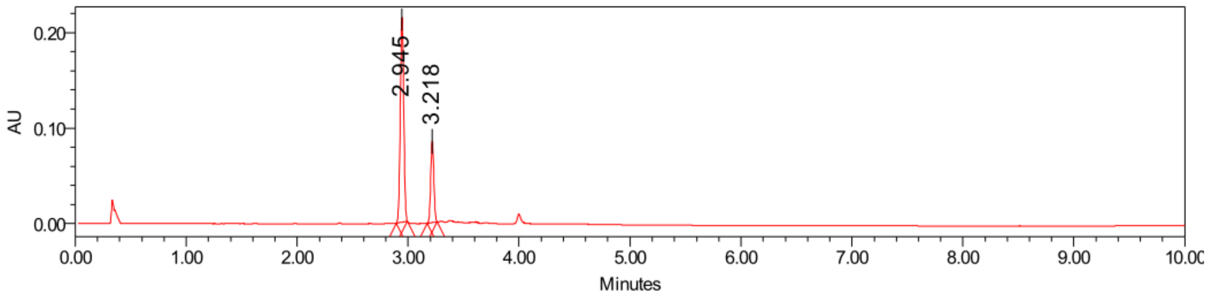

	RT	Area	% Area	Height
1	2.945	465774	73.49	215170
2	3.218	168022	26.51	85605

(*S*)-4-Isobutyl-(2-chlorophenyl)-((*R*)-4-oxochroman-2-yl)-2-1,3-oxazol-5(4*H*)-one (3g)

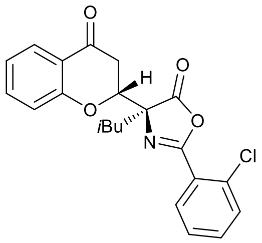

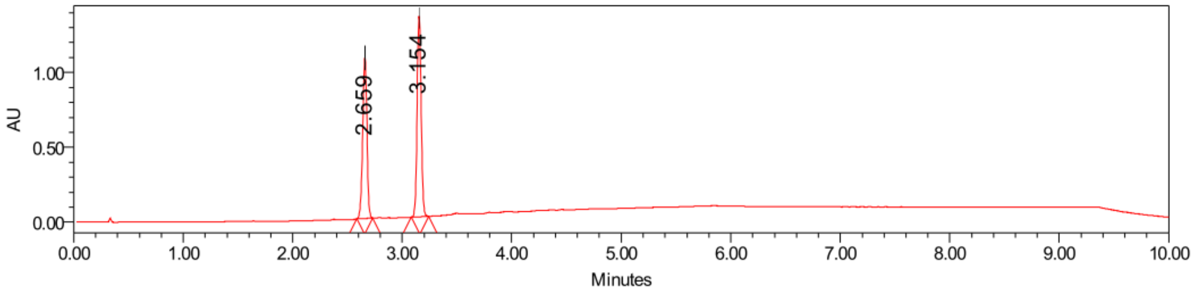

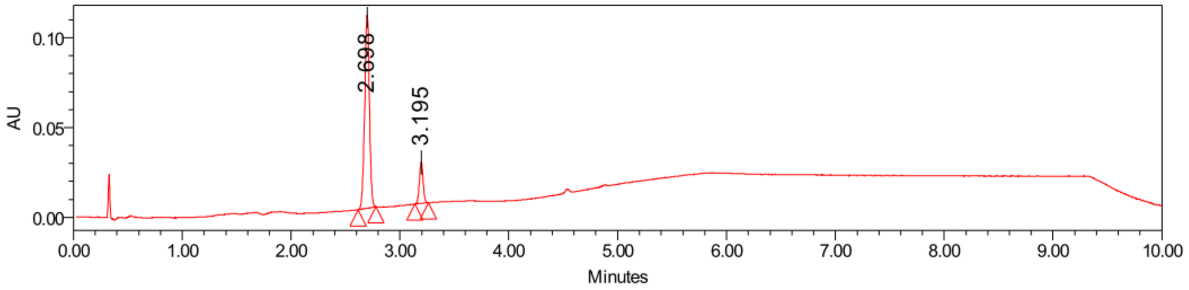

	RT	Area	% Area	Height
1	2.698	331834	85.79	107452
2	3.195	54966	14.21	22919

(*S*)-4-Isobutyl-(4-chlorophenyl)-((*R*)-4-oxochroman-2-yl)-2-1,3-oxazol-5(4*H*)-one (3h)

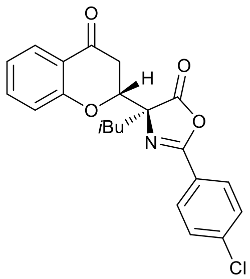

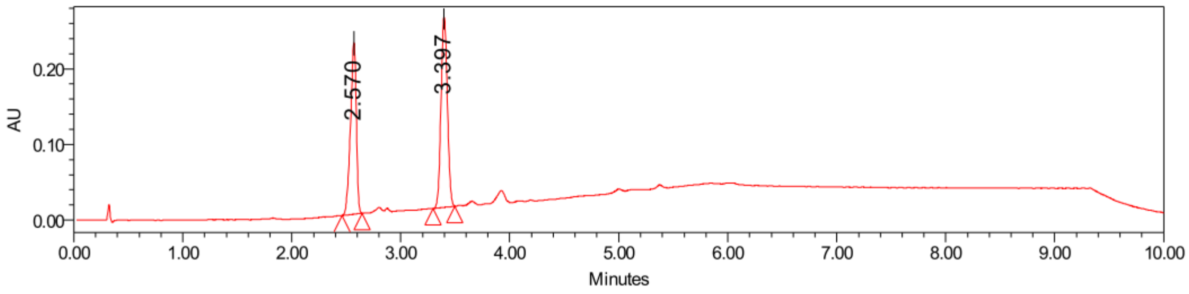

Minutes

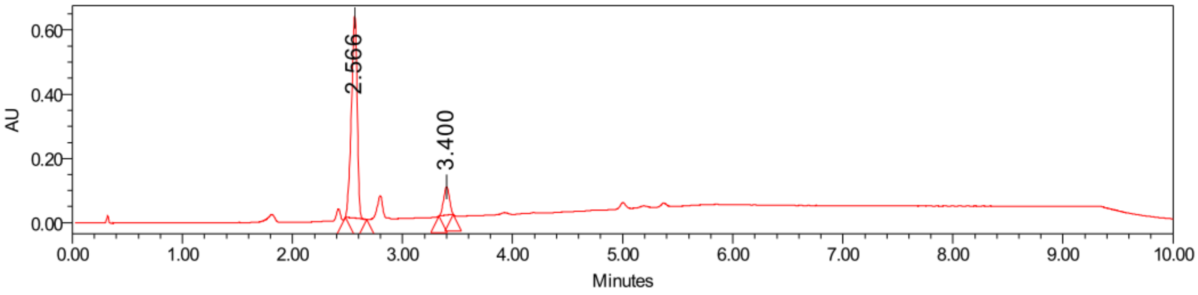

	RT	Area	% Area	Height
1	2.566	2275562	87.65	629463
2	3.400	320670	12.35	87827

(*S*)-4-Isobutyl-2-(4-nitrophenyl)((*R*)-4-oxochroman-2-yl)-1,3-oxazol-5(4*H*)-one (3i)

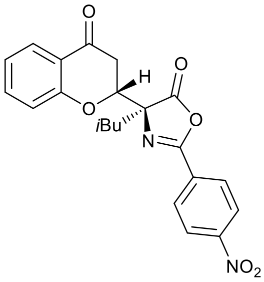

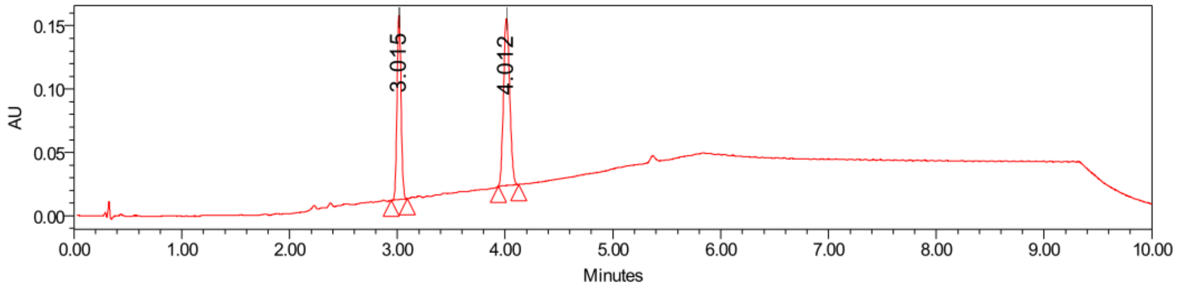

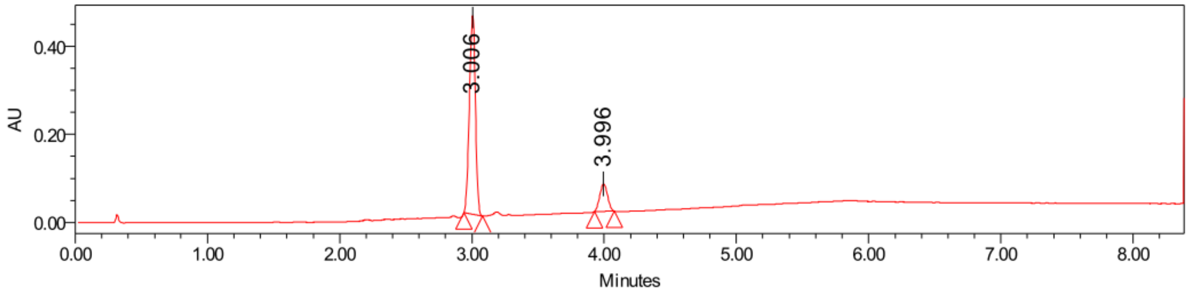

	RT	Area	% Area	Height
1	3.006	1433611	84.79	451630
2	3.996	257165	15.21	62490

(*S*)-4-Isobutyl-(6-fluoro-(*R*)-4-oxochroman-2-yl)-2-phenyl-1,3-oxazol-5(4*H*)-one (3j)

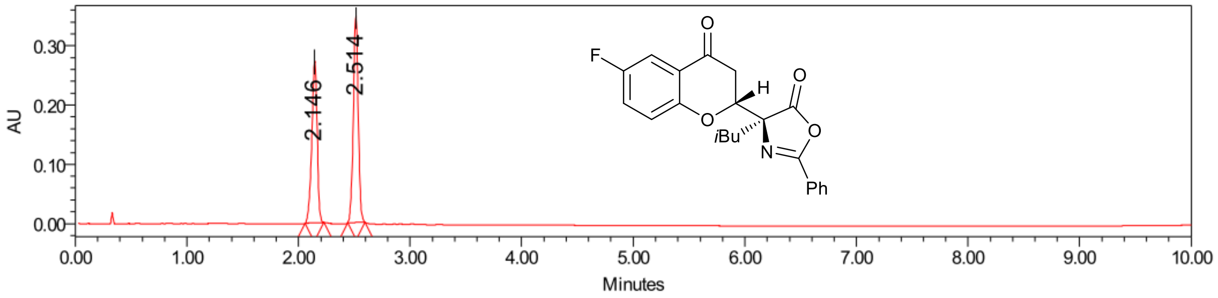

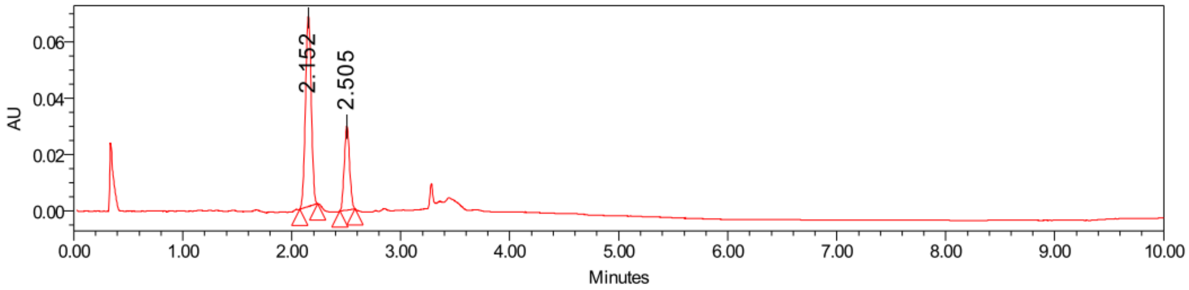

	RT	Area	% Area	Height
1	2.152	247393	71.04	67526
2	2.505	100837	28.96	29692

(6-Bromo-(*R*)-4-oxochroman-2-yl)-(*S*)-4-isobutyl-2-phenyl-1,3-oxazol-5(4*H*)-one (3k)

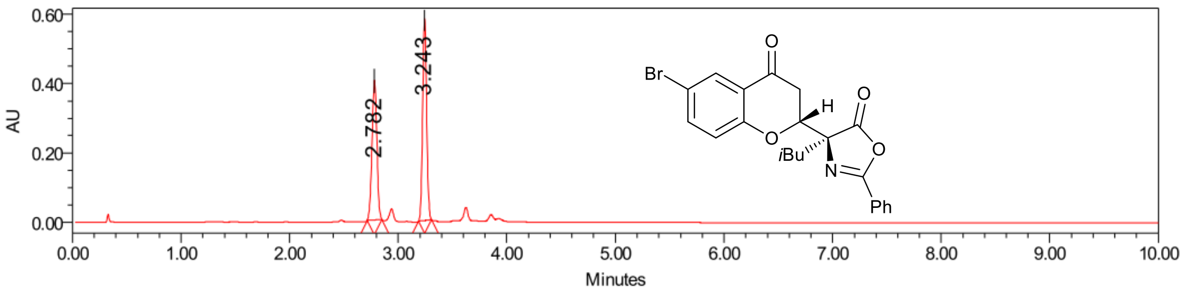

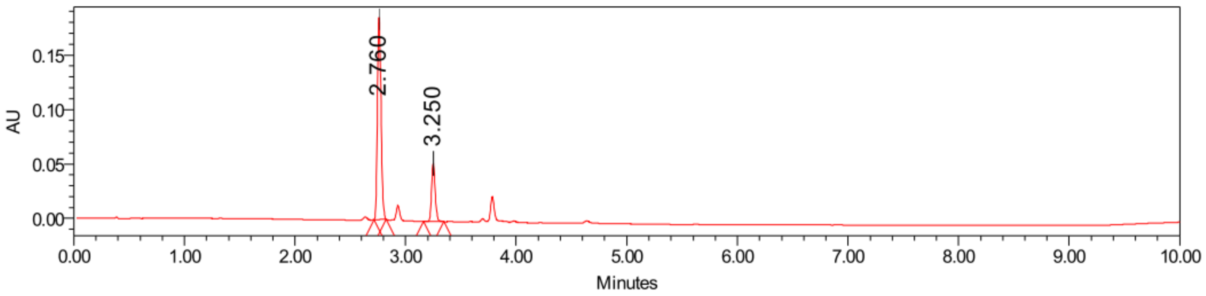

	RT	Area	% Area	Height
1	2.760	419227	77.44	185649
2	3.250	122122	22.56	53584

(6-Chloro-(*R*)-4-oxochroman-2-yl)-(*S*)-4-isobutyl-2-phenyl-1,3-oxazol-5(4*H*)-one (3l)

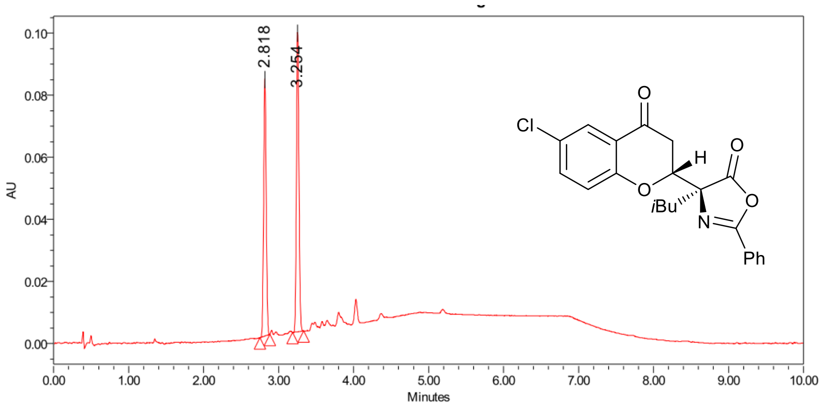

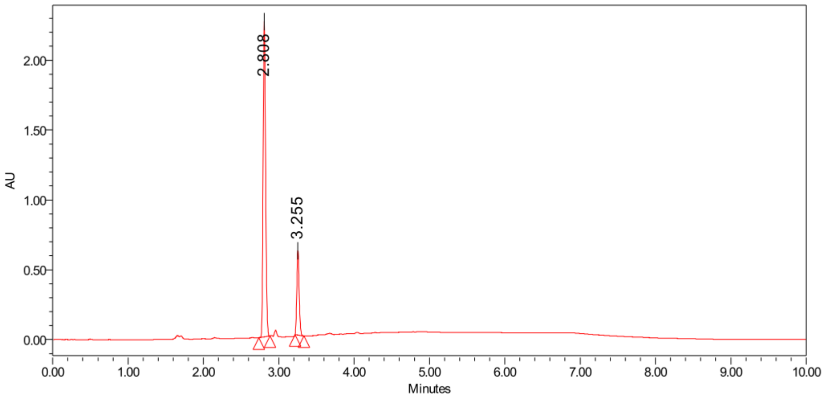

Peak Results

	Name	RT	Area	Height	Amount	Units	% Area
1		2.808	5159625	2269413			79.52
2		3.255	1328498	606304			20.48

(*S*)-4-Isobutyl-(6-nitro-(*R*)-4-oxochroman-2-yl)-2-phenyl-1,3-oxazol-5(4*H*)-one (3m)

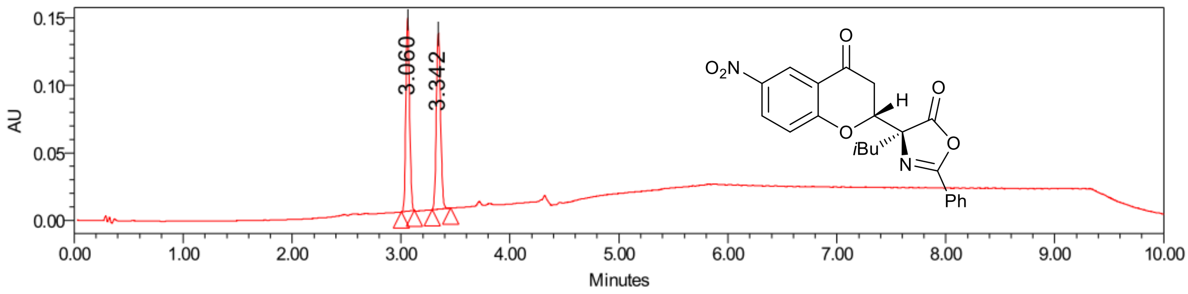

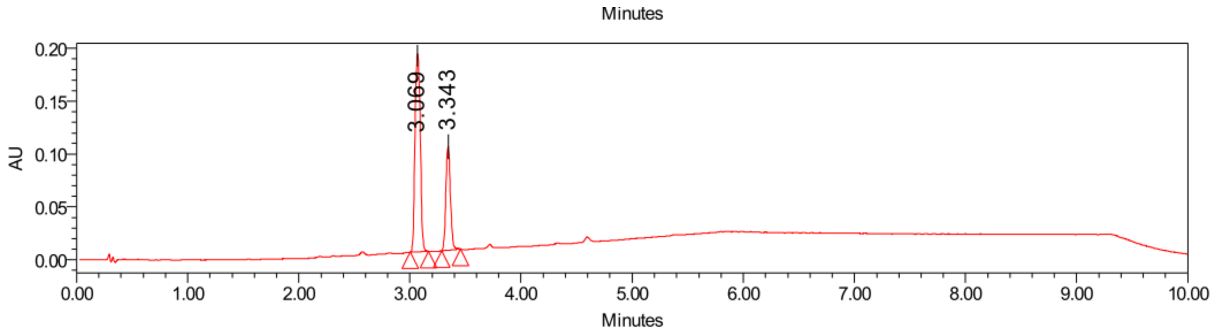

	RT	Area	% Area	Height
1	3.069	611483	68.77	187664
2	3.343	277717	31.23	97896

(*S*)-4-Isobutyl-(6-methyl-(*R*)-4-oxochroman-2-yl)-2-phenyl-1,3-oxazol-5(4*H*)-one (3n)

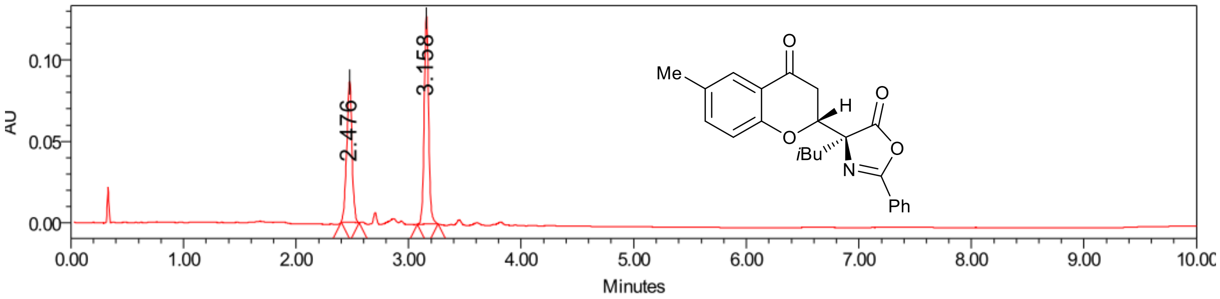

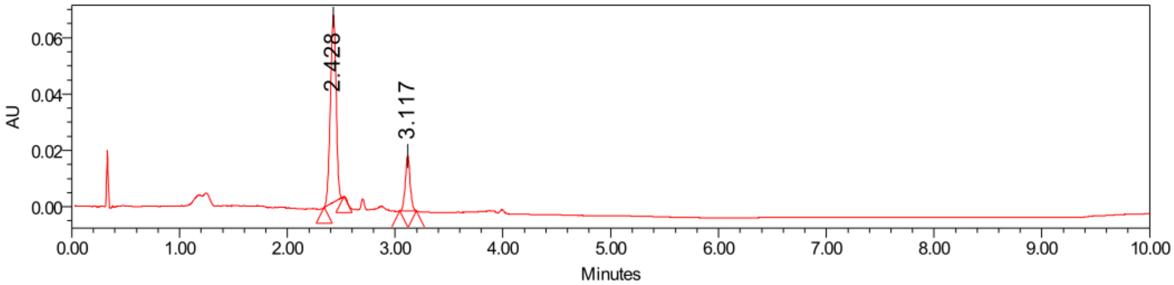

	RT	Area	% Area	Height
1	2.428	244078	79.83	66417
2	3.117	61659	20.17	19597

(*S*)-4-Isobutyl-(7-methoxy-(*R*)-4-oxochroman-2-yl)-2-phenyl-1,3-oxazol-5(4*H*)-one (3o)

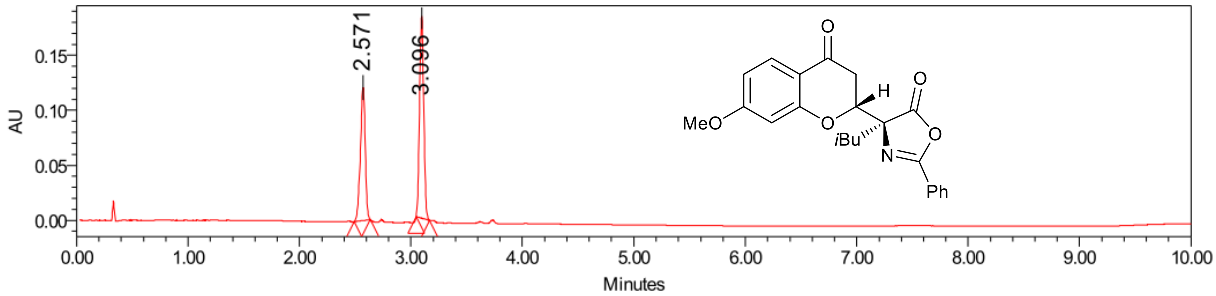

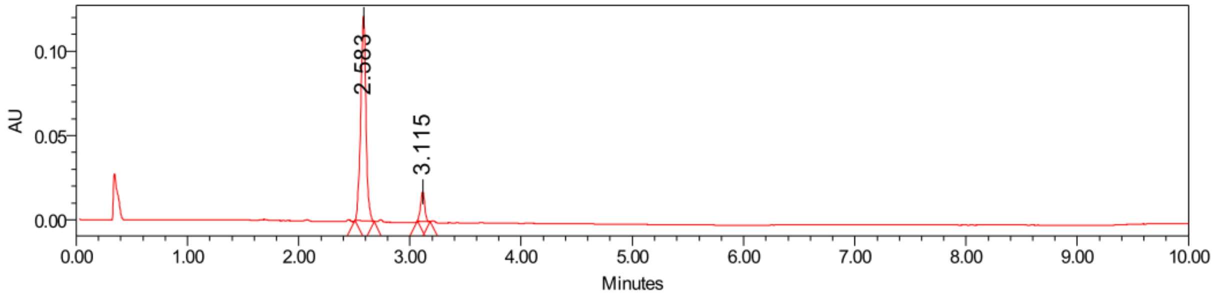

	RT	Area	% Area	Height
1	2.583	396265	89.76	121534
2	3.115	45214	10.24	17544

(*S*)-4-Isobutyl-(6-chloro-7-methyl-(*R*)-4-oxochroman-2-yl)-2-phenyl-1,3-oxazol-5(4*H*)-one (3p)

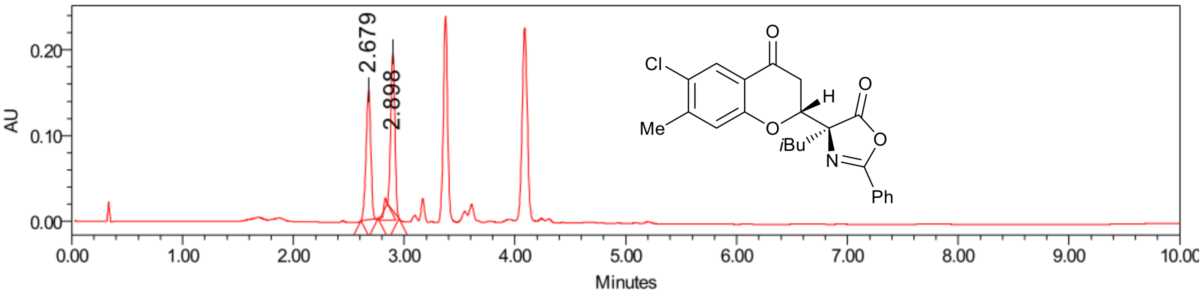

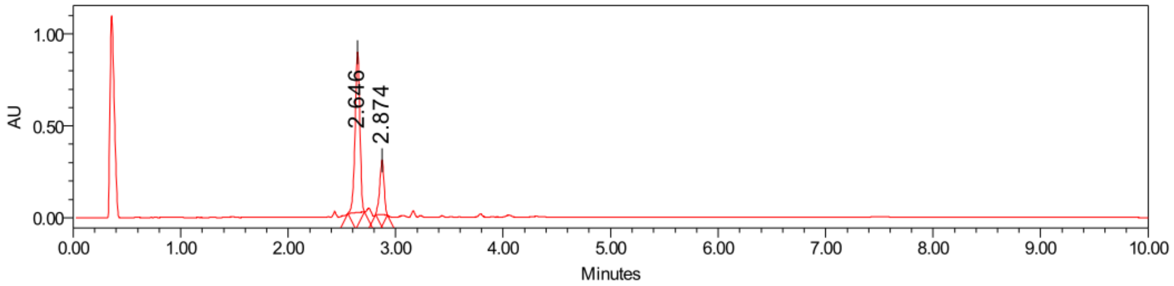

	RT	Area	% Area	Height
1	2.646	2687401	76.27	872873
2	2.874	836009	23.73	298350
